# Exact Ground State of Interacting Electrons in Magic Angle Graphene

**DOI:** 10.1007/s00220-025-05300-x

**Published:** 2025-05-30

**Authors:** Simon Becker, Lin Lin, Kevin D. Stubbs

**Affiliations:** 1 ETH Zurich, Institute for Mathematical Research, Rämistrasse 101, 8092 Zurich, Switzerland; 2 Department of Mathematics, University of California, Berkeley, CA 94720 USA; 3 Applied Mathematics and Computational Research Division, Lawrence Berkeley National Laboratory, Berkeley, CA 94720 USA

## Abstract

One of the most remarkable theoretical findings in magic angle twisted bilayer graphene (TBG) is the emergence of ferromagnetic Slater determinants as exact ground states for the interacting Hamiltonian at the chiral limit. This discovery provides an explanation for the correlated insulating phase which has been experimentally observed at half filling. This work is the first mathematical study of interacting models in magic angle graphene systems. These include not only TBG but also TBG-like systems featuring four flat bands per valley, and twisted trilayer graphene systems with equal twist angles. We identify symmetries of the chiral limit of the Bistritzer-MacDonald Hamiltonian that are responsible for characterizing the Hartree-Fock ground states as zero energy many-body ground states. Furthermore, for a general class of Hamiltonian, we establish criteria that the ferromagnetic Slater determinants are the unique ground states within the class of uniformly half-filled, translation invariant Slater determinants. We then demonstrate that these criteria can be explicitly verified for TBG and TBG-like systems at the chiral limit, using properties of Jacobi-${\theta }$ and Weierstrass-${\wp }$ functions.

## Introduction

Over the past few years, moiré structures, notably “magic angle” twisted bilayer graphene (TBG), have attracted significant attention in the condensed matter physics community. This surge of interest began after numerous experiments [1–5] which demonstrate that TBG has nearly flat energy bands and can host intricate phases, such as the correlated insulator (CI) phases at integer fillings and superconducting (SC) phases at non-integer fillings. Before these exciting experimental results however, the Bistritzer-MacDonald (BM) model [6] successfully predicted these nearly flat energy bands near the magic angle of roughly $1.1^{\circ }$. Yet, the BM model does not directly include electron–electron repulsion (i.e., it is a non-interacting model) and in fact, due to symmetry restrictions, the BM model predicts that twisted bilayer graphene is always in a simple metallic phase. As a result, the CI and SC phases must arise from electron–electron interactions beyond the non-interacting BM model.

At the chiral limit when the effects of the non-flat bands are excluded, we can define a flat-band interacting (FBI) Hamiltonian for TBG that only consists of terms describing electron–electron interactions [7–19]. One of the most remarkable theoretical results on this model is that, at half filling, the FBI Hamiltonian is *frustration-free*, i.e., the Hamiltonian can be written as a sum of terms (in this case, these terms do not commute), and there exists a ground state that minimizes the energy of each term. For the FBI Hamiltonian, one possible ground state is a single Slater determinant, which is the simplest type of fermionic many-body wavefunctions. Moreover, it can be shown that for this ground state, in the thermodynamic limit, a strictly positive amount of energy is needed to either add or remove an electron under certain assumptions. This offers *one* possible explanation for the CI phase in TBG at half-filling.

To our knowledge, mathematical studies so far have focused on understanding the BM model of twisted bilayer and multilayer graphene at the chiral limit [20–27], and systematic derivations of the BM Hamiltonian from more complex models such as the atomistic tight binding models [28–30]. In this paper, we provide the *first* mathematical study of interacting systems, and specifically the FBI Hamiltonian and its ground states. We note that our analysis applies to the FBI Hamiltonian defined on a single valley of the moiré Brillouin zone.

Our analysis is based on the symmetries of the BM Hamiltonian at the chiral limit. These symmetries are preserved as the number of layers and flat bands varies, which allows us to study TBG as well as TBG-like systems, including twisted multilayer graphene structures where the ratio between the relative twist angles are rational numbers. For example, the standard chiral model for TBG has two layers and two flat bands; a system we refer to as TBG-2. By changing the interlayer potential, it is possible for two layers of graphene to host four flat bands while still preserving the relevant symmetries [23]; we refer to this system as TBG-4. We can also consider a twisted trilayer graphene (TTG) system, where the relative twist between the top and middle layers, and the relative twist between the middle and bottom layers are the same. This model can host four flat bands [31], and we refer to this system as the equal angle twisted trilayer graphene (eTTG-4).

Although the FBI Hamiltonian is an idealized and simplified representation of interacting electrons, it still contains many intricate details. Our paper does not discuss the derivation of the FBI Hamiltonian from the BM model, or the derivation of the BM model from the finer level atomistic models. We assume readers are familiar with second quantization and standard treatments of non-interacting periodic systems.

### Notation

In this paper, operators and matrices acting on the Fock space are represented using the hat notation, such as ${\hat{f}}^{\dag }, {\hat{f}}, {\hat{H}}_{\textrm{FBI}}$. Operators and matrices that operate in the single particle space (such as $L^2({\mathbb {R}}^2;{\mathbb {C}}^2\otimes {\mathbb {C}}^2)$ for TBG) are indicated without the hat notation, as seen in *H*, *D*. For a single particle operator *H*, the Bloch-Floquet transformed Hamiltonian is denoted by $H_\textbf{k}$, where $\textbf{k}$ is the Brillouin zone index. With some slight abuse of the hat notation, vectors defined in real space are denoted without the hat, for example, $u(\textbf{r})$. Their corresponding Fourier transforms are indicated with the hat notation, as in ${\hat{u}}(\textbf{q})$. For any given matrix *A*, the operations of entrywise complex conjugation, transpose, and Hermitian conjugation are represented by ${\overline{A}}, A^{\top },$ and $A^{\dag }$, respectively.

Due to the symmetries present in twisted graphene systems, the number of flat bands is always an even number which we will denote by 2*M*. We denote the set of indices of the flat bands by $\mathcal {N}=\{ -M, \cdots , -1, 1, \cdots , M\}$. After a proper discretization of a single valley of the moiré Brillouin zone into a discrete set $\mathcal {K}$ with $N_{\textbf{k}}$ points (see the definition of $\mathcal {K}$ in Eq. (2.13)), we can consider a finite dimensional Hilbert space $\mathcal {F}$ consisting of $2M N_{\textbf{k}}$ fermionic modes. Vectors and linear operators on ${\mathcal {F}}$ can be specified using the fermionic creation and annihilation operators ${\hat{f}}_{n\textbf{k}}^{\dag }, {\hat{f}}_{n\textbf{k}}$ which satisfy the canonical anticommutation relation (CAR), and the vacuum state $\vert \textrm{vac}\rangle$ which satisfies ${\hat{f}}_{n\textbf{k}}\vert \textrm{vac}\rangle =0$ for each $n \in \mathcal {N}$ and $\textbf{k}\in \mathcal {K}$. Taking the convention that $({\hat{f}}_{n \textbf{k}}^{\dagger })^{0} = 1$ and $({\hat{f}}_{n \textbf{k}}^{\dagger })^{1} = {\hat{f}}_{n \textbf{k}}^{\dagger }$, this space is spanned by $2^{2M N_{\textbf{k}}}$ basis vectors of the form

1.1 $$\begin{aligned} \vert s_{(1, \textbf{k}_{1})}, s_{(-1, \textbf{k}_{1})},\ldots ,s_{(M, \textbf{k}_{N_{\textbf{k}}})}, s_{(-M, \textbf{k}_{N_{\textbf{k}}})}\rangle = \prod _{n\in \mathcal {N}}\prod _{\textbf{k}\in {\mathcal {K}}}({\hat{f}}^{\dag }_{n\textbf{k}})^{s_{n \textbf{k}}}\vert \textrm{vac}\rangle , \quad s_{n \textbf{k}}\in \{0,1\}.\nonumber \\ \end{aligned}$$

The FBI Hamiltonian takes the form

1.2 $$\begin{aligned} {\hat{H}} = \sum _{\textbf{q}'} {\hat{O}}_{\textbf{q}'}^{\dag } {\hat{O}}_{\textbf{q}'}, \end{aligned}$$

where each ${\hat{O}}_{\textbf{q}'}$ takes the form

1.3 $$\begin{aligned} {\hat{O}}_{\textbf{q}'} = \sum _{\textbf{k}\in \mathcal {K}}\sum _{m, n \in \mathcal {N}} [\alpha _{\textbf{k},\textbf{k}+\textbf{q}'}]_{m,n} {\hat{f}}^{\dag }_{m\textbf{k}} {\hat{f}}_{n(\textbf{k}+ \textbf{q}')} + [\beta _{\textbf{k},\textbf{k}+\textbf{q}'}]_{m,n}. \end{aligned}$$

Here, the sum over $\textbf{q}' \in \mathbb {R}^{2}$ is taken over a discrete set which will be specified later; $\textbf{q}'$ represents a momentum difference and so the terms in ${\hat{O}}_{\textbf{q}'}$ connect states at momentum $\textbf{k}$ and $\textbf{k}+ \textbf{q}'$. The coefficient matrices $\alpha _{\textbf{k},\textbf{k}+\textbf{q}'}$ and $\beta _{\textbf{k},\textbf{k}+\textbf{q}'}$ are matrices of size $2 M \times 2M$ (defined in Eq. (2.25)), depend on the eigenstates of the non-interacting BM Hamiltonian at momenta $\textbf{k}$ and $\textbf{k}+ \textbf{q}'$, and inherit a number of symmetries from the BM Hamiltonian.

The total number operator ${\hat{N}}=\sum _{n\in \mathcal {N}} \sum _{\textbf{k}} {\hat{f}}^{\dag }_{n\textbf{k}} {\hat{f}}_{n\textbf{k}}$ counts the number of electrons in a state, i.e., if ${\hat{N}}\vert \psi \rangle =\nu N_{\textbf{k}}\vert \psi \rangle$, then the number of electrons in $\vert \psi \rangle$ is $\nu N_{\textbf{k}}$. The number operator ${\hat{N}}$ commutes with ${\hat{H}}$ so we can restrict to functions that are simultaneously eigenfunctions of ${\hat{H}}$ and ${\hat{N}}$. The integer filling regime refers to the case when $\nu$ is an integer ($0\le \nu \le 2M$). One particular integer filling is $\nu =M (= \frac{1}{2} (2M))$, and the corresponding $\vert \psi \rangle$ is also called a *half-filled* state. If we further have $\sum _{n\in \mathcal {N}} {\hat{f}}^{\dag }_{n\textbf{k}} {\hat{f}}_{n\textbf{k}}\vert \psi \rangle =M\vert \psi \rangle$ for all $\textbf{k}\in \mathcal {K}$, then $\vert \psi \rangle$ is a *uniformly half-filled* state. The one-body reduced density matrix (1-RDM) is defined as $[P(\textbf{k},\textbf{k}')]_{nm}=\langle \psi |{\hat{f}}^{\dag }_{m\textbf{k}'} {\hat{f}}_{n\textbf{k}}|\psi \rangle$. If $[P(\textbf{k},\textbf{k}')]_{nm}=[P(\textbf{k})]_{nm} \delta _{\textbf{k},\textbf{k}'}$, then $\vert \psi \rangle$ is called a *translation invariant* state.

This paper considers the uniformly half-filled, translation invariant states, which forms a subspace of $\mathcal {F}$ denoted by $\mathcal {F}_u$. The ground-state energy in $\mathcal {F}_u$ is the solution to the following optimization problem

1.4 $$\begin{aligned} E=\min _{\vert \psi \rangle \in \mathcal {F}_u} \frac{\langle \psi |{\hat{H}}|\psi \rangle }{\langle \psi |\psi \rangle }, \end{aligned}$$

and its minimizer (which may not be unique) is called a ground state. For any $\vert \psi \rangle \in \mathcal {F}$, by construction $\langle \psi |{\hat{H}}|\psi \rangle =\sum _{\textbf{q}'} \langle {\hat{O}}_{\textbf{q}'} \psi |{\hat{O}}_{\textbf{q}'} \psi \rangle \ge 0$. Therefore ${\hat{H}}$ is a positive semidefinite (PSD) Hamiltonian, and a state $\vert \psi \rangle \in \mathcal {F}_u$ satisfying ${\hat{H}}\vert \psi \rangle =0$ must be a ground state.

We further consider a subset of $\mathcal {F}_u$

1.5 $$\begin{aligned} \mathcal {S}=\left\{ \prod _{i=1}^{M} \prod _{\textbf{k}\in \mathcal {K}} {\hat{b}}_{i\textbf{k}}^{\dag }\vert \textrm{vac}\rangle \Big \vert ~{\hat{b}}^{\dag }_{i\textbf{k}}=\sum _{n\in {\mathcal {N}}}{\hat{f}}^{\dag }_{n\textbf{k}}[\Xi (\textbf{k})]_{ni}, \quad \sum _{n \in {\mathcal {N}}} [\overline{\Xi (\textbf{k})}]_{ni} [\Xi (\textbf{k})]_{nj}=\delta _{ij}\right\} .\nonumber \\ \end{aligned}$$

Each element of $\mathcal {S}$ is called a uniformly half-filled, translation invariant *Slater determinant*. The *Hartree-Fock* theory solves a much simpler optimization problem

1.6 $$\begin{aligned} E_{\text {HF}}=\min _{\begin{array}{c} \vert \psi \rangle \in \mathcal {S} \end{array}} \frac{\langle \psi |{\hat{H}}|\psi \rangle }{\langle \psi |\psi \rangle }. \end{aligned}$$

By definition we have $E\le E_{\text {HF}}$ and in general $E< E_{\text {HF}}$.

### Main results

In this section, we summarize the main findings of this paper, emphasizing the algebraic structures, while reserving some of the more technical specifics for later in the paper.

Our first result is that there exist states in $\mathcal {S}$ which are exact ground states of the FBI Hamiltonian. This also implies that the Hartree-Fock theory is exact in this case.

#### Result 1

(Informal version of Proposition 5.2). There exists two states $\vert \Psi _{\pm }\rangle \in {\mathcal {S}}$ satisfying

1.7 $$\begin{aligned} {\hat{O}}_{\textbf{q}'} \vert \Psi _{\pm }\rangle =0 \end{aligned}$$

for all $\textbf{q}'$. Therefore $E=E_{\operatorname {HF}}=0$, and $\vert \Psi _{\pm }\rangle$ are exact ground states of ${\hat{H}}$.

The proof of Result 1 is a generalization of the results in [7, 13] for TBG. The mechanism of constructing these exact ground states is to verify that $\vert \Psi _{\pm }\rangle$ are ground states of *each* ${\hat{H}}_{\textbf{q}'}={\hat{O}}^{\dag }_{\textbf{q}'} {\hat{O}}_{\textbf{q}'}$, even though the terms ${\hat{H}}_{\textbf{q}'}$ do not commute with each other. Therefore the FBI Hamiltonian is an example of a frustration-free Hamiltonian with nonlocal interactions.

However, there may be other ground states. For instance, any non-vanishing linear combination $a_{+}\vert \Psi _{+}\rangle +a_{-}\vert \Psi _{-}\rangle$ is automatically a ground state. It is in general a difficult task to identify the entire ground state manifold. Our main result identifies additional assumptions under which $\vert \Psi _{\pm }\rangle$ are the *unique* states^1^ in $\mathcal {S}$ that are ground states of ${\hat{H}}$.

#### Result 2

(Main result. Informal version of Theorem 5). Assume that the set of matrices $\{ \alpha _{\textbf{k},\textbf{k}+\textbf{q}'}, \beta _{\textbf{k},\textbf{k}+\textbf{q}'}\}$ satisfy additional non-degeneracy assumptions (Theorem 5), then $\vert \Psi _{\pm }\rangle$ in Result 1 are the unique states in $\mathcal {S}$ that are exact ground states ${\hat{H}}$.

Both Results 1 and 2 apply to general FBI Hamiltonians which can be used to describe the interacting electrons in twisted *N*-layer graphene systems. The non-degeneracy assumptions can be explicitly verified for specific systems. We verify these conditions for TBG-2, TBG-4, and eTTG-4 systems using the analytic expression for $\{\alpha _{\textbf{k},\textbf{k}+\textbf{q}'},\beta _{\textbf{k}+\textbf{q}'}\}$. This leads to the following results.

#### Result 3

(Informal version of Theorems 10, 11, 12) For TBG-2, TBG-4, and eTTG-4, $\vert \Psi _{\pm }\rangle$ in Result 1 are the unique states in $\mathcal {S}$ that are exact ground states of ${\hat{H}}$.

While Results 1 and 2 provide a characterization of the ground state, these results alone do not explain why the uniformly half-filled TBG-2 system (and similar systems) are insulators. In Sect. 5.4, we prove that adding or removing an electron to a ferromagnetic Slater determinant always costs a non-zero amount of energy, which remains non-zero in the thermodynamic limit ($N_{\textbf{k}}\rightarrow \infty$). Even though the precise nature of the many-body excited state remains unclear, this shows that the system cannot be in a simple metallic state.

### Discussion and open questions

The states $\vert \Psi _{\pm }\rangle$ are sometimes referred to as “ferromagnetic Slater determinants”. This term is inherited from the physics context where, due to our construction, we can identify $\vert \Psi _{+}\rangle$ with $\vert \uparrow \uparrow \uparrow \cdots \rangle$ and $\vert \Psi _{-}\rangle$ with $\vert \downarrow \downarrow \downarrow \cdots \rangle$, which are ground states of a ferromagnet. The bundles whose sections correspond to states $\vert \Psi _{\pm }\rangle$ have nonvanishing Chern numbers, and are also referred to as (integer) quantum hall states [32], or Chern ferromagnetic states.

The mechanism for the uniqueness of the ferromagnetic Slater determinant ground states in twisted graphene is rather different from previous uniqueness results in many-body systems (for example, on the Hubbard model on a planar graph [33–35]). Unlike these results, the FBI Hamiltonian involves interactions between all momenta and the ground state is not determined by the connectivity of the underlying graph structure. Instead, the selection of ferromagnetic Slater determinant as the ground states seems to be related to the joint invariant subspaces of the collection of matrices $\{\alpha _{\textbf{k},\textbf{k}+\textbf{q}'},\beta _{\textbf{k}+\textbf{q}'}\}$; any state whose one-body reduced density matrix does not lie in an invariant subspace acquires an energy penalty.

These conditions in Result 2 are physically relevant. This paper focuses on the spinless, valleyless (or in physical terms, spin and valley polarized) FBI Hamiltonian. When valley degrees of freedom are taken into account, these assumptions become invalid. This leads to additional ground states in $\mathcal {S}$, such as the inter-valley coherent (IVC) states [7, 13]. On the other hand, the conditions in Result 2 are difficult to verify. They can be relaxed and explicitly verified for $M=1$ or 2, as shown in Result 3. However, for $M>2$, a generally computationally verifiable method for reformulating these conditions remains unknown.

Even when the conditions are satisfied, Result 2 only establishes uniqueness among uniformly half-filled, translation-invariant Slater determinants. Our follow-up work [36] extends the uniqueness to all single Slater determinants for TBG-2 using a different technique, which also takes into account the additional spins and valleys degrees of freedom. However, extension of the uniqueness result for models beyond TBG-2 is currently unknown.

Finally, an important limitation of the current work is that the definition of FBI Hamiltonian only incorporates electron–electron interactions from the flat bands. While the flat bands electrons strongly influence the interacting behavior of twisted graphene systems, the effect from the filled remote bands remains significant [10, 37], and this has motivated the development of more complex models such as the topological heavy fermion model [38]. The study of interacting models with remote bands is an interesting direction for future research.

### Organization

Our article is structured as follows:

- In Sect. 2 we define the flat-band interacting model for twisted graphene systems.
- In Sect. 3 we review the chiral model of twisted graphene sheets.
- In Sect. 4, we outline the symmetries that are relevant for our analysis and fix a gauge of Bloch functions.
- In Sect. 5, we review basics on Hartree-Fock theory for FBI Hamiltonians and prove some of their important many-body properties.
- In Sect. 6, we characterize the ground states of the flat band interacting model and state our main result, Theorem 5.
- In Sect. 7, we give the proof of our main result, Theorem 5.
- In Sect. 8, we verify the assumptions of Theorem 5 for TBG-2, TBG-4, and eTTG-4.
- Our article contains four technical appendices, Sects.  A, B, C and D where we state the proof of Lemma 4.1, derive expressions for the Hartree and Fock energies (Eqs. (5.6) and (5.7)), derive Eq. (7.13), and derive a real space condition for the assumptions of Theorem 5, respectively.

## The Flat-Band Interacting Hamiltonian for Twisted Graphene

### Notational setup for twisted graphene

Before defining the flat-band interacting model for twisted graphene, we introduce a general notation for twisted *N*-layer graphene based on the Bistritzer-MacDonald model. Twisted bilayer and trilayer graphene correspond to choosing $N = 2$ and $N = 3$ respectively.

After a proper choice of units, the lattice vectors for the moiré unit cell in the real space are

2.1 $$\begin{aligned} \textbf{v}_1:= -\begin{bmatrix}\frac{2\pi }{\sqrt{3}},\frac{2\pi }{3}\end{bmatrix}^\top \qquad \textbf{v}_{2}:= \begin{bmatrix}\frac{2\pi }{\sqrt{3}}, - \frac{2\pi }{3}\end{bmatrix}^\top . \end{aligned}$$

These vectors generate the real space moiré lattice $\Gamma$:

2.2 $$\begin{aligned} \Gamma := \textbf{v}_{1} {\mathbb {Z}}+ \textbf{v}_{2} {\mathbb {Z}}= \{ a_{1} \textbf{v}_{1} + a_{2} \textbf{v}_{2}: a_{1}, a_{2} \in {\mathbb {Z}}\}. \end{aligned}$$

The moiré unit cell in the real space is denoted by $\Omega := \mathbb {R}^{2} / \Gamma$ and can be identified with

2.3 $$\begin{aligned} \Omega := \left\{ t_1 \textbf{v}_1 + t_2 \textbf{v}_2; t_1,t_2 \in \left[ -\frac{1}{2},\frac{1}{2} \right) \right\} . \end{aligned}$$

The dual lattice (or the reciprocal lattice) of $\Gamma$ is denoted by $\Gamma ^{*}$, and is generated by

2.4 $$\begin{aligned} \textbf{g}_1:= -\begin{bmatrix}\frac{\sqrt{3}}{2},\frac{3}{2}\end{bmatrix}^\top \qquad \textbf{g}_{2}:= \begin{bmatrix}\frac{\sqrt{3}}{2},- \frac{3}{2}\end{bmatrix}^\top . \end{aligned}$$

Note that $\textbf{v}_{i} \cdot \textbf{g}_{j} = 2 \pi \delta _{ij}$. The unit cell in the reciprocal space (or the Brillouin zone) is denoted by $\Omega ^{*}:= \mathbb {R}^{2} / \Gamma ^{*}$ and can be identified with

2.5 $$\begin{aligned} \Omega ^* =\left\{ t_1 \textbf{g}_1 + t_2 \textbf{g}_2: t_1,t_2 \in \left[ -\frac{1}{2},\frac{1}{2}\right) \right\} . \end{aligned}$$

Throughout the paper, a vector in the reciprocal lattice $\Gamma ^*$ is often denoted by $\textbf{G}$, while a vector in the Brillouin zone $\Omega ^*$ is often denoted by $\textbf{k}$ or $\textbf{q}$. Note that a generic vector $\textbf{q}'\in {\mathbb {R}}^2$ can always be uniquely decomposed as $\textbf{q}'=\textbf{q}+\textbf{G}$ for some $\textbf{q}\in \Omega ^*$ and $\textbf{G}\in \Gamma ^*$.

In addition to the generating vectors $\textbf{g}_{1}$ and $\textbf{g}_{2}$, we also identify three special momentum vectors $\textbf{q}_{1}, \textbf{q}_{2}, \textbf{q}_{3}$ which are related to each other by a $\frac{2 \pi }{3}$-counterclockwise rotation $R_{3}$:

$$\begin{aligned} R_{3}:= \frac{1}{2} \begin{bmatrix} -1 &  -\sqrt{3} \\ \sqrt{3} &  -1 \end{bmatrix}. \end{aligned}$$

In particular,

2.6 $$\begin{aligned} \textbf{q}_{1}:= [0, 1]^{\top } \quad \textbf{q}_{2}:= R_{3} \textbf{q}_{1} = [-\sqrt{3}/2, -1/2]^{\top } \quad \textbf{q}_{3}:= R_{3} \textbf{q}_{2} = [\sqrt{3}/2, -1/2]^{\top }\nonumber \\ \end{aligned}$$

Notice that $\textbf{g}_{1} = \textbf{q}_{2} - \textbf{q}_{1}$ and $\textbf{g}_{2} = \textbf{q}_{3} - \textbf{q}_{1}$.

Each moiré unit cell consists of many atoms located on one of the two sublattices, denoted by $\{ A = 1, B = -1\}$. In the BM model for twisted bilayer and multilayer graphene, the atomistic details within each layer are averaged out, retaining only the sublattice information. The BM Hamiltonian *H* (called a single particle Hamiltonian) acts on a dense subset of the function space $L^2({\mathbb {R}}^2;{\mathbb {C}}^2\otimes {\mathbb {C}}^N)$, where *N* is the number of layers. A function in $L^2({\mathbb {R}}^2;{\mathbb {C}}^2\otimes {\mathbb {C}}^N)$ can be written as $\psi (\textbf{r};\sigma ,\ell )$, where $\textbf{r}=[x_1,x_2]^{\top }\in {\mathbb {R}}^2$ is the real space coordinate, $\sigma \in \{\pm 1\}$ is the sublattice index, and $\ell \in \{1,\ldots ,N\}$ is the layer index. The BM Hamiltonian satisfies a translation symmetry with respect to the moiré lattice $\Gamma$. Therefore an eigenfunction of *H* can be labeled as $\psi _{n\textbf{k}}(\textbf{r};\sigma ,\ell )$. Here *n* is called the band index, $\textbf{k}= (k_{1}, k_{2})\in \Omega ^*$ is the Bloch vector. For example, a wavefunction for the Hamiltonian of TBG can be written as

2.7 $$\begin{aligned} \begin{bmatrix} \psi _{n\textbf{k}}(\textbf{r}; A, 1)&\psi _{n\textbf{k}}(\textbf{r}; A, 2)&\psi _{n\textbf{k}}(\textbf{r}; B, 1)&\psi _{n\textbf{k}}(\textbf{r}; B, 2) \end{bmatrix}^{\top } \end{aligned}$$

and for TTG:

2.8 $$\begin{aligned} \begin{bmatrix} \psi _{n\textbf{k}}(\textbf{r}; A, 1)&\psi _{n\textbf{k}}(\textbf{r}; A, 2)&\psi _{n\textbf{k}}(\textbf{r}; A, 3)&\psi _{n\textbf{k}}(\textbf{r}; B, 1)&\psi _{n\textbf{k}}(\textbf{r}; B, 2)&\psi _{n\textbf{k}}(\textbf{r}; B, 3) \end{bmatrix}^{\top }.\nonumber \\ \end{aligned}$$

### The Flat-Band Interacting (FBI) Hamiltonian

While we adopt first quantization in expressing the BM Hamiltonian (see Sect. 3), the FBI Hamiltonian is most conveniently expressed in second quantization. The FBI Hamiltonian is defined in terms of the flat-band eigenfunctions of the single particle Hamiltonian *H*; therefore, our first step will be to construct such eigenfunctions.

We consider $H=\sum _{\vert \alpha \vert \le s} a_{\alpha }(x)D_{x}^{\alpha }$ with $D_{x}^{\alpha }=\prod _{j} (-i \partial _{x_i})^{\alpha _i}$ with $\alpha \in \mathbb {N}_0^2$ and $s \in \mathbb {N}$ is a self-adjoint elliptic^2^ differential operator with $a_{\alpha } \in C^{\infty }(\mathbb {R}^n/\Gamma ;\mathbb {C}^{2N \times 2N})$ that is periodic with respect to the lattice $\Gamma .$ We then define the Bloch-Floquet transformed Hamiltonian $H_{\textbf{k}}:= e^{-i \textbf{k}\cdot \textbf{r}} H e^{i \textbf{k}\cdot \textbf{r}}$ with domain $H^s(\Omega )$, for some $s>0$, on the Hilbert space $L^2(\Omega )$ for all $\textbf{k}\in \Omega ^*$. This operator $H_\textbf{k}$ has Bloch eigenpairs $\{ (\epsilon _{n\textbf{k}}, u_{n\textbf{k}}): n \in I, \textbf{k}\in \Omega ^{*} \}$, where $I \subset \mathbb {Z}$ is some index set so that the eigenvalues $\epsilon _{n\textbf{k}}$ are ordered in non-decreasing order and

2.9 $$\begin{aligned} \begin{aligned} H_{\textbf{k}} u_{n\textbf{k}}(\textbf{r})&= \epsilon _{n\textbf{k}} u_{n\textbf{k}}(\textbf{r}) \text { and } u_{n\textbf{k}}(\textbf{r}+ \textbf{a}) =u_{n\textbf{k}}(\textbf{r}) \quad \forall \textbf{a}\in \Gamma \text { and }\\ \psi _{n\textbf{k}}(\textbf{r})&:=e^{i \textbf{k}\cdot \textbf{r}} u_{n\textbf{k}}(\textbf{r}). \end{aligned} \end{aligned}$$

From the specific form of the Hamiltonian, it follows that

- If we identify $\textbf{k}\in \Omega ^*$ with the complex number $k_{x_1} + i k_{x_2} \in {\mathbb {C}}$, the operator valued complex map $\textbf{k}\mapsto (H_{\textbf{k}} - i)^{-1}$ depends real-analytically on $(k_{x_1},k_{x_2})$.
- The Bloch functions $u_{n\textbf{k}}(\textbf{r})$ are smooth as a function of $\textbf{r}$ by elliptic regularity.

For any such *H*, we define the set of flat bands as follows:

2.10 $$\begin{aligned} \mathcal {N}:= \{ n \in I: \forall \textbf{k}\in \Omega ^{*}, \,\epsilon _{n\textbf{k}} = 0 \} \end{aligned}$$

When the flat bands ${\mathcal {N}}$ are isolated from the remaining bands, we can define the spectral projector, $\Pi (\textbf{k})$, onto the flat bands. In this case, the form of *H* then implies the map $\textbf{k} \mapsto \Pi (\textbf{k})$ is real analytic:

#### [Style2 Style2]Proposition 2.1

Let $H_{\textbf{k}}$ be the Hamiltonian defined above, and let $(\varepsilon _{n\textbf{k}})_{n \in I}$ be a family of Bloch bands for some finite index set *I*, which are uniformly (in $\textbf{k}$) gapped from the remaining Bloch bands, then the spectral projection $\Pi (\textbf{k}):=\sum _{n \in I} u_{n\textbf{k}}\otimes u_{n\textbf{k}}$ depends real-analytically on $(k_x,k_y)$ where $\textbf{k}=k_x+ik_y.$

#### Proof

This is a direct consequence of the formula

$$\begin{aligned} \Pi (\textbf{k}) = (2\pi i)^{-1} \int _{\gamma } (z-H_{\textbf{k}})^{-1} d\textbf{k},\end{aligned}$$

where $\gamma$ is a contour that encloses $(\varepsilon _{n\textbf{k}})_{n \in I}$ but none of the remaining eigenvalues, and the real-analyticity of the map $(k_x,k_y) \mapsto (H_{\textbf{k}}-i)^{-1}$. $\square$

**Fig. 1:**
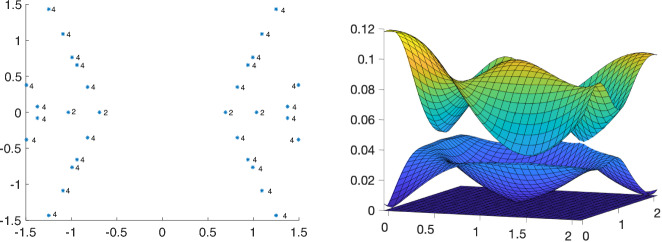
Magic angles with multiplicities in the chiral limit of 7 equally twisted layers of graphene with multiplicities and $U_+=U_0$, see (3.14). Band configuration for largest magic angle $\alpha = 0.6922$ showing the six lowest bands with positive energy including the four flat bands showing three flat band crossings at high symmetry points

In some systems, such as twisted trilayer graphene (TTG), there can be energy bands which intersect with the flat bands but are not flat themselves. This can happen in all *N*-layer systems with $N\ge 3$ (see Fig. 1 for an example). Let us define such the set of momenta where such crossings occur as follows:

2.11 $$\begin{aligned} \mathcal {K}_{\textrm{crossing}} = \{ \textbf{k}\in \Omega ^{*}: \epsilon _{n\textbf{k}} = 0, \text { for some }n \not \in \mathcal {N} \}. \end{aligned}$$

For such systems, we define the flat band space at $\textbf{k}\in \mathcal {K}_{\textrm{crossing}}$ via continuity. That is, for each $\textbf{k}_{*} \in {\mathcal {K}}_{\textrm{crossing}}$ we define

2.12 $$\begin{aligned} \Pi (\textbf{k}_{*}) = \lim _{\begin{array}{c} \textbf{k} \rightarrow \textbf{k}_{*}\\ \textbf{k} \not \in \mathcal {K}_{\textrm{crossing}} \end{array}} \Pi (\textbf{k}). \end{aligned}$$

While the above limit may not exist (see, e.g. [39] for a counter example), for all the examples we consider, we will show explicitly that the flat band projector defined by continuity is smooth in $(k_{x_{1}}, k_{x_{2}})$. In the case of TBG-2 and TBG-4, the projector depends real-analytically on $(k_{x_{1}}, k_{x_{2}})$ this will be a direct consequence of Corollary 2.1, since the remaining bands are gapped from the flat ones by [24, Theo. 2] and [23, Theorem 4], respectively. In the case of eTTG-4, we will prove that a smooth map $\Pi (\textbf{k})$ can be constructed using the structure of the flat band wavefunctions and a partition of unity argument. This is discussed more thoroughly just before Theorems 10, 11, and 12, respectively.

For our proofs, we make the following assumptions on $\mathcal {N}$ and the flat band projector $\Pi (\textbf{k})$:

#### Assumption 1

We assume that *H* takes the form as above and has a non-empty set of flat bands $\mathcal {N}:= \{ n \in I: \forall \textbf{k}\in \Omega ^{*}, \, \epsilon _{n\textbf{k}} = 0 \}.$ We also assume the spectral projector onto the flat bands $\Pi (\textbf{k})$ (potentially defined through a limit) is periodic and Lipschitz continuous with respect to $\textbf{k}$.

In order to write down a many-body wavefunction with a finite number of particles, we discretize the Brillouin zone using a uniform grid $\mathcal {K}$ of size $(n_{k_x}, n_{k_y})$

2.13 $$\begin{aligned} \mathcal {K}:= \left\{ \frac{i}{n_{k_{x}}} \textbf{g}_1 + \frac{j}{n_{k_{y}}} \textbf{g}_2: i \in \{0, 1, \cdots , n_{k_{x}} - 1\}, j \in \{0, 1, \cdots , n_{k_{y}} - 1 \} \right\} \subseteq \Omega ^*.\nonumber \\ \end{aligned}$$

Here $\textbf{g}_1$, $\textbf{g}_2$ are a pair of generating vectors for the moiré dual lattice $\Gamma ^{*}$, see (2.4), and we define $N_{\textbf{k}}:= \# | \mathcal {K} | = n_{k_{x}} n_{k_{y}}$.

The set $\mathcal {K}$ is known as a Monkhorst-Pack grid, and the *thermodynamic limit* (TDL) can be reached by taking $n_{k_x},n_{k_y}\rightarrow \infty$. We also make the following technical assumption on the grid $\mathcal {K}$ in relation to the Hamiltonian *H*:

#### Assumption 2

We assume that $n_{k_{x}} > L$ and $n_{k_{y}} > L$ where *L* is the Lipschitz constant of the map ${\textbf{k}} \mapsto \Pi ({\textbf{k}})$.

#### Remark 1

The choice of Monkhorst-Pack grid is not strictly necessary for the main result. The main results will still apply for any grid which (1) contains $\textbf{0}$, (2) is closed under addition and subtraction modulo $\Gamma ^{*}$, and (3) if $\textbf{k}$ and $\textbf{k}'$ are neighboring points on the grid then $\Vert \Pi (\textbf{k}) - \Pi (\textbf{k}') \Vert < 1$.

Having fixed the grid $\mathcal {K}$ and the flat-band projector, $\Pi (\textbf{k})$, we may now define the FBI Hamiltonian. For each $\textbf{k}\in \mathcal {K}$, let $u_{n\textbf{k}}(\textbf{r})$ for $n \in \mathcal {N}$ be an orthonormal basis for $\Pi (\textbf{k})$ and let $\psi _{n \textbf{k}}(\textbf{r}) = e^{i \textbf{k}\cdot \textbf{r}} u_{n \textbf{k}}(\textbf{r})$. Since $\psi _{n \textbf{k}}$ are Bloch eigenfunctions of the Hamiltonian *H*, we define the band creation and annihilation operators, ${\hat{f}}_{n\textbf{k}}^\dagger$ and ${\hat{f}}_{n\textbf{k}}$, which create or annihilate a particle in state $\psi _{n\textbf{k}}(\textbf{r})$ respectively, which satisfy the canonical anti-commutation relation (CAR):

2.14 $$\begin{aligned} \{{\hat{f}}^{\dag }_{n\textbf{k}},{\hat{f}}_{n'\textbf{k}'}\} = \delta _{nn'} \delta _{\textbf{k}\textbf{k}'}, \quad \{{\hat{f}}^{\dag }_{n\textbf{k}},{\hat{f}}^{\dag }_{n'\textbf{k}'}\} =\{{\hat{f}}_{n\textbf{k}},{\hat{f}}_{n'\textbf{k}'}\}=0. \end{aligned}$$

The definition of the creation and annihilation operators can be periodically extended outside the Brillouin zone as

2.15 $$\begin{aligned} {\hat{f}}^{\dag }_{n(\textbf{k}+\textbf{G})}={\hat{f}}^{\dag }_{n\textbf{k}}, \quad {\hat{f}}_{n(\textbf{k}+\textbf{G})}={\hat{f}}_{n\textbf{k}}, \quad \textbf{G}\in \Gamma ^*. \end{aligned}$$

To define the flat band interacting model, we first consider the *pair product* defined as follows:

2.16 $$\begin{aligned} [\rho _{\textbf{k}, (\textbf{k} + \textbf{q})}(\textbf{r})]_{mn} = \sum _{\sigma , j}  \overline{u_{m \textbf{k}}(\textbf{r}; \sigma , j)} u_{n (\textbf{k} + \textbf{q})}(\textbf{r}; \sigma , j). \end{aligned}$$

For each $\textbf{r} \in \Omega$, the pair product $\rho _{\textbf{k}, \textbf{k} + \textbf{q}}(\textbf{r})$ is a matrix of size $\# | {\mathcal {N}} | \times \# | {\mathcal {N}} |$, whose entries are pointwise products between the periodic Bloch functions at momentum $\textbf{k}$ and $\textbf{k} + \textbf{q}$ summed over sublattice and layer. Note that since periodic Bloch functions are periodic with respect to $\Gamma$, the pair product is also periodic with respect to $\Gamma$. Therefore, we can define the Fourier series coefficients of the pair product as follows:

2.17 $$\begin{aligned} [{\hat{\rho }}_{\textbf{k}, (\textbf{k} + \textbf{q})}(\textbf{G})]_{mn} = \int _{\Omega } e^{-i \textbf{G} \cdot \textbf{r}} [\rho _{\textbf{k}, (\textbf{k} + \textbf{q})}(\textbf{r})]_{mn} \,\textrm{d}\textbf{r}. \end{aligned}$$

The standard convention in the TBG literature is to define interacting models in terms of the *form factor*, denoted $\Lambda _{\textbf{k}}(\textbf{q} + \textbf{G})$ where $\textbf{k}, \textbf{q} \in \Omega ^{*}$ and $\textbf{G} \in \Gamma ^{*}$, which is related to the pair product by the following identification

2.18 $$\begin{aligned} \begin{aligned} \,[\Lambda _{\textbf{k}}(\textbf{q} + \textbf{G})]_{mn}&:= [{\hat{\rho }}_{\textbf{k}, (\textbf{k} + \textbf{q})}(\textbf{G})]_{mn} \\&= \frac{1}{| \Omega |} \sum _{\textbf{G}' \in \Gamma ^*} \sum _{\sigma ,j}\overline{{\hat{u}}_{m\textbf{k}}(\textbf{G}'; \sigma , j)} {\hat{u}}_{n(\textbf{k}+ \textbf{q}+\textbf{G})}(\textbf{G}'; \sigma , j) \end{aligned} \end{aligned}$$

where ${\hat{u}}_{n\textbf{k}}(\textbf{G}; \sigma , j)$ denotes the Fourier coefficients of $u_{n\textbf{k}}(\textbf{r}; \sigma , j)$

2.19 $$\begin{aligned} {\hat{u}}_{n\textbf{k}}(\textbf{G}; \sigma , j):= \int _{\Omega } e^{-i \textbf{G}\cdot \textbf{r}} u_{n\textbf{k}}(\textbf{r}; \sigma , j) d\textbf{r}. \end{aligned}$$

We can write the form factor more suggestively as an inner product over $\Gamma ^{*}$ and $\sigma , j$

2.20 $$\begin{aligned} [\Lambda _{\textbf{k}}(\textbf{q} + \textbf{G})]_{mn} = \frac{1}{| \Omega |} \langle {\hat{u}}_{m\textbf{k}}, {\hat{u}}_{n(\textbf{k}+ \textbf{q}+\textbf{G})}\rangle . \end{aligned}$$

From this expression, we can interpret the form factor as the inner product between periodic Bloch functions at momentum $\textbf{k}$ and $\textbf{k} + \textbf{q} + \textbf{G}$. Hence, the subscript, $\textbf{k}$, in the form factor, $\Lambda _{\textbf{k}}(\textbf{q} + \textbf{G})$, can be thought of as the starting momentum and argument, $\textbf{q} + \textbf{G}$, can be thought of as a momentum offset.

The following identities which will be useful at various points in our proof.

#### Lemma 2.2

The form factor matrix $\Lambda _{\textbf{k}}(\textbf{q}+ \textbf{G})$ satisfies the following identities for all $\textbf{k},\textbf{q}\in \mathcal {K}$ and all $\textbf{G}, \textbf{G}' \in \Gamma ^*$

2.21 $$\begin{aligned} \Lambda _{\textbf{k}}(\textbf{q}+ \textbf{G})^{\dagger }&= \Lambda _{\textbf{k}+\textbf{q}}(-\textbf{q}-\textbf{G}), \end{aligned}$$

2.22 $$\begin{aligned} \Lambda _{\textbf{k}+ \textbf{G}'}(\textbf{q}+ \textbf{G})&= \Lambda _{\textbf{k}}(\textbf{q}+ \textbf{G}). \end{aligned}$$

#### Proof

This follows from the following simple calculation

2.23 $$\begin{aligned} \begin{aligned} \Lambda _{\textbf{k}}(\textbf{q}+ \textbf{G})^{\dagger }&= \frac{1}{| \Omega |} \sum _{\textbf{G}' \in \Gamma ^*} \sum _{\sigma ,j}{\hat{u}}_{n\textbf{k}}(\textbf{G}'; \sigma , j) \overline{{\hat{u}}_{m(\textbf{k}+ \textbf{q}+ \textbf{G})}(\textbf{G}'; \sigma , j)} \\&= \frac{1}{| \Omega |} \sum _{\textbf{G}' \in \Gamma ^*} \sum _{\sigma ,j}\overline{{\hat{u}}_{m(\textbf{k}+ \textbf{q}+ \textbf{G})}(\textbf{G}'; \sigma , j)} {\hat{u}}_{n\textbf{k}}(\textbf{G}'; \sigma , j) \\&= \Lambda _{\textbf{k}+\textbf{q}}(-\textbf{q}- \textbf{G}). \end{aligned} \end{aligned}$$

As for the second identity, one easily checks that ${\hat{u}}_{n(\textbf{k}+ \textbf{G}')}(\textbf{G}) ={\hat{u}}_{n\textbf{k}}(\textbf{G}+ \textbf{G}')$ and so

2.24 $$\begin{aligned} \begin{aligned} \Lambda _{{\textbf {k}}+ {\textbf {G}}'}({\textbf {q}}+ {\textbf {G}})&= \frac{1}{| \Omega |} \sum _{{\textbf {G}}'' \in \Gamma ^*} \sum _{\sigma ,j} \overline{{\hat{u}}_{m({\textbf {k}}+ {\textbf {G}}')}({\textbf {G}}''; \sigma , j)} {\hat{u}}_{n({\textbf {k}}+ {\textbf {q}}+ {\textbf {G}}+ {\textbf {G}}')}({\textbf {G}}''; \sigma , j) \\  &= \frac{1}{| \Omega |} \sum _{{\textbf {G}}'' \in \Gamma ^*} \sum _{\sigma ,j} \overline{{\hat{u}}_{m{\textbf {k}}}({\textbf {G}}'' + {\textbf {G}}'; \sigma , j)} {\hat{u}}_{n({\textbf {k}}+ {\textbf {q}}+ {\textbf {G}})}({\textbf {G}}'' + {\textbf {G}}'; \sigma , j) \\  &= \Lambda _{{\textbf {k}}}({\textbf {q}}+ {\textbf {G}}). \end{aligned} \end{aligned}$$

$$\square $$

We can now define the flat-band interacting (FBI) Hamiltonian for the twisted *N*-layer graphene [7, 13].

#### Definition 2.3

Let ${\hat{f}}_{m\textbf{k}}^{\dagger }, {\hat{f}}_{m\textbf{k}}$ denote the flat-band creation and annihilation operators, and let $\Lambda _{\textbf{k}}(\textbf{q}+ \textbf{G})$ be defined as in Eq. (2.18). The flat-band interacting (FBI) Hamiltonian is:

2.25 $$\begin{aligned} \begin{aligned} {\hat{H}}_{\textrm{FBI}}&:= \frac{1}{N_{\textbf{k}} |\Omega |} \sum _{\textbf{q}' \in \mathcal {K} + \Gamma ^*} {\hat{V}}(\textbf{q}') {\widehat{\rho }}(\textbf{q}') {\widehat{\rho }}(-\textbf{q}') \\ {\widehat{\rho }}(\textbf{q}')&:= \sum _{\textbf{k}\in \mathcal {K}} \sum _{m,n \in \mathcal {N}} [\Lambda _{\textbf{k}}(\textbf{q}')]_{mn} \left( {\hat{f}}_{m\textbf{k}}^\dagger {\hat{f}}_{n(\textbf{k}+\textbf{q}')} - \frac{1}{2} \delta _{mn} \sum _{\textbf{G}}\delta _{\textbf{q}',\textbf{G}} \right) . \end{aligned} \end{aligned}$$

Here, ${\hat{V}}(\textbf{q}') = {\hat{V}}(|\textbf{q}'|)$ is the Fourier transform of a radially symmetric, smooth electron–electron potential which satisfies ${\hat{V}}(\textbf{q}') > 0$ for all $\textbf{q}' \in \mathbb {R}^2$.

When we compare the definition of ${\hat{H}}_{\textrm{FBI}}$ with that in Eqs. 1.2, 1.3, we need to show that ${\hat{\rho }}^{\dag }(\textbf{q}')={\hat{\rho }}(-\textbf{q}')$, and that ${\hat{H}}_{\textrm{FBI}}$ is a positive semi-definite Hamiltonian. These relations will be verified in Sect. 5.2.

For instance, for TBG, the double gate-screened Coulomb interaction in Fourier space reads (Note that $\lim _{\textbf{q}' \rightarrow \varvec{0}} {\hat{V}}(\textbf{q}') = \frac{\pi d}{\varepsilon }$ is well defined)

2.26 $$\begin{aligned} {\hat{V}}(\textbf{q}')=\frac{2\pi }{\epsilon }\frac{\tanh (\left|\textbf{q}'\right|d/2)}{\left|\textbf{q}'\right|}. \end{aligned}$$

Here $\epsilon ,d>0$ parametrize the strength and length of the screened Coulomb interaction, respectively (see e.g., [13, Appendix C]).

## A Review of Twisted Bilayer Graphene and equal Twist Angle Trilayer Graphene

To make the assumptions of Result 2 more concrete, we review the properties of the TBG and eTTG [20, 22, 28, 29, 31, 40] in the Bistritzer-MacDonald Hamiltonian at the chiral limit. The single particle Hamiltonian for both chiral TBG and chiral eTTG depend on a parameter $\alpha$, that is inversely proportional to the twisting angle, and take the form of a matrix-valued differential operator

3.1 $$\begin{aligned} H(\alpha ) = \begin{bmatrix} 0 &  D(\alpha )^\dag \\ D(\alpha ) &  0 \end{bmatrix}, \end{aligned}$$

For TBG, $D(\alpha )$ acts on $H^{1}(\mathbb {R}^{2}; {\mathbb {C}}^{2})$

3.2 $$\begin{aligned} D_{\textrm{TBG}}(\alpha ) = \begin{bmatrix} D_{x_1}+i D_{x_2} &  \alpha U_+(\textbf{r}) \\ \alpha U_-( \textbf{r}) &  D_{x_1}+i D_{x_2} \end{bmatrix}, \end{aligned}$$

and for eTTG, $D(\alpha )$ acts on $H^{1}(\mathbb {R}^{2}; {\mathbb {C}}^{3})$

3.3 $$\begin{aligned} D_{\textrm{eTTG}}(\alpha ) = \begin{bmatrix} D_{x_1}+i D_{x_2} &  \alpha U_+(\textbf{r}) &  0 \\ \alpha U_-( \textbf{r}) &  D_{x_1}+i D_{x_2} &  \alpha U_+(\textbf{r}) \\ 0 & \alpha U_-( \textbf{r}) &  D_{x_1}+i D_{x_2} \end{bmatrix}. \end{aligned}$$

Similarly, single particle Hamiltonians for *N*-layer systems can be defined in terms of operators $D(\alpha )$ acting on $H^{1}(\mathbb {R}^{2}; {\mathbb {C}}^{N})$ (see, for example, [26]).

We remark that the specific form of the single particle Hamiltonian will not be used in our proofs of Results 1, 2 and 3, however we will make use of two important properties which hold for Bistritzer-MacDonald-type Hamiltonians at the chiral limit: (1) the existence of a magic angle with exactly flat bands, and (2) the single particle Hamiltonian commutes with a number of symmetry operations (see Sect. 4).

For small twist angles, due to the lattice structure of graphene, the tunneling potentials $U_{\pm }(\textbf{r})$ satisfy the following symmetry properties independent of the number of layers [6, 40]:

3.4 $$\begin{aligned} U_{\pm }(\textbf{r}+ \textbf{a})&= {\overline{\omega }}^{\pm (a_1+a_2)} U_{\pm }(\textbf{r}), \end{aligned}$$

3.5 $$\begin{aligned} U_{\pm }(R_{3} \textbf{r})&= \omega U_{\pm }(\textbf{r}), \end{aligned}$$

3.6 $$\begin{aligned} U_{\pm }(x_{1}, -x_{2})&= \overline{U_{\pm }(x_{1}, x_{2})}, \end{aligned}$$

where $\textbf{r}= [x_{1}, x_{2}]^{\top }$, $\textbf{a}=a_1 \textbf{v}_1 + a_2 \textbf{v}_2 \in \Gamma$, $\omega := e^{2 \pi i / 3}$, and $R_{3}$ is the $\frac{2 \pi }{3}$-counterclockwise rotation. Potentials $U_{\pm }$ satisfying symmetries Eqs. (3.4), (3.5) and (3.6) are of the general form

3.7 $$\begin{aligned} U_{\pm }(\textbf{r})=\sum _{n,m\in {\mathbb {Z}}} c_{nm} e^{\pm i (m \textbf{g}_{1} - n \textbf{g}_{2} + \textbf{q}_{1}) \cdot \textbf{r}} \end{aligned}$$

where we recall the definitions of the dual lattice vectors $\textbf{g}_1,\textbf{g}_2,$ (Eq. (2.4)) and $\textbf{q}_{1}$ (Eq. (2.6)). The coefficients $c_{nm}$ satisfy, see [22, Prop.2.1],

$$\begin{aligned}\begin{aligned} c_{nm}&=\omega c_{(m-n-1)(-n)}=\omega ^{2} c_{(-m)(n-m+1)}\text { and }\\ \overline{c_{nm}}&=c_{(-m)(-n)}=\omega c_{(-n+m-1)m}=\omega ^{2} c_{n(n-m+1)} \text { for all }n, m \in {\mathbb {Z}}^2.\end{aligned} \end{aligned}$$

It is important to note that due to Eq. (3.4), both TBG and eTTG as defined above are not periodic with respect to $\Gamma$, however they can both be made into periodic Hamiltonian by performing a unitary transformation (see Sect. 3.3). While this non-periodic formulation is more convenient for the analysis of the magic angles; we will use the periodic formulation in our definition of the FBI Hamiltonians (Eq. (2.25)).

### Magic angles in TBG

The TBG Hamiltonian (Eq. (3.1) with Eq. (3.2)) commutes with the following symmetries

3.8 $$\begin{aligned} \mathcal {T}_{\textbf{a}} \textbf{u}(\textbf{r}) = \begin{bmatrix} \omega ^{a_{1} + a_{2}} & & &  \\ &  1 & &  \\ & &  \omega ^{a_{1} + a_{2}} &  \\ & & &  1 \end{bmatrix} \textbf{u}(\textbf{r}+\textbf{a}), \qquad \mathcal {R} \textbf{u}(\textbf{r}) = \begin{bmatrix} 1 & & &  \\ &  1 & &  \\ & &  {\overline{\omega }} &  \\ & & &  {\overline{\omega }} \end{bmatrix} \textbf{u}(R_{3} \textbf{r}). \end{aligned}$$

Therefore, we can define

3.9 $$\begin{aligned} L^2_{\ell ,p}:= \{ \textbf{u}\in L^2_{\text {loc}}({\mathbb {R}}^2;{\mathbb {C}}^4); \mathcal {T}_{\textbf{a}} \textbf{u}= \omega ^{\ell (a_1+a_2)} \textbf{u}, \text { for } \textbf{a}\in \Gamma \text { and } \mathcal {R} \textbf{u}= {\overline{\omega }}^p \textbf{u}\} \end{aligned}$$

where $\ell ,p \in {\mathbb {Z}}_3.$ We may also consider symmetries (3.8) just acting on ${\mathbb {C}}^2$-valued spinors by considering merely the first two components. Thus, by defining $\pi : \mathbb {C}^2 \rightarrow \mathbb {C}^4$ and $\pi (u)=(u_1,u_2,0,0)^{\top },$ we define for $\mathbb {C}^2$ valued functions the analogous spaces

$$\begin{aligned} L^2_{\ell ,p}  &   :=\{ \textbf{u}\in L^2_{\text {loc}}({\mathbb {R}}^2;{\mathbb {C}}^2); \mathcal {T}_{\textbf{a}} \pi (\textbf{u}) = \omega ^{\ell (a_1+a_2)} \pi (\textbf{u}), \text { for } \textbf{a}\in \Gamma \text { and } \mathcal {R} \pi (\textbf{u}) \\    &   = {\overline{\omega }}^p \pi (\textbf{u})\}. \end{aligned}$$

Whether we are working with the $\mathbb {C}^2$ or $\mathbb {C}^4$ valued spaces will be clear from context.

The $\mathcal {T}_{\textbf{a}}$-periodicity of the TBG Hamiltonian in Eq. (3.1) allows us to define the Bloch-Floquet transformed TBG Hamiltonian

3.10 $$\begin{aligned} H_{\textbf{k}}(\alpha ) = \begin{bmatrix} 0 &  D_{\textrm{TBG}}(\alpha )^\dag +( k_1-ik_2) \operatorname {id}_{\mathbb {C}^2} \\ D_{\textrm{TBG}}(\alpha ) + (k_1+ik_2 )\operatorname {id}_{\mathbb {C}^2}&  0 \end{bmatrix} \end{aligned}$$

acting on $$L^2_{\ell }:=\bigoplus _{p \in {\mathbb {Z}}_3} L^2_{\ell ,p}$$.

The TBG Hamiltonian additionally has a layer symmetry $\mathcal {L}$ which acts as follows

3.11 $$\begin{aligned} \mathcal {L} \textbf{v}(\textbf{r}):= \begin{bmatrix} & & &  -1 \\ & &  1 &  \\ &  -1 & &  \\ 1 & & &  \\ \end{bmatrix} \textbf{v}(-\textbf{r}). \end{aligned}$$

One furthermore can check that $\mathcal {L}: L^{2}_{\ell ,p} \rightarrow L^{2}_{-\ell +1,p}$. In particular, for $\ell =2$ the map $\mathcal {L}$ leaves spaces $L^2_{2,p}$ invariant. Thus, we shall without loss of generality, consider $H_{{\textbf{k}}}(\alpha )$ on $L^2_{\ell =2}$ in the sequel, since the family of $H_{{\textbf{k}}}(\alpha )$ on $L^2_{\ell }$ are all equivalent. To see this observe that multiplying by ${\mathscr {U}}(\textbf{r}){:}{=}e^{i/3(\langle {\textbf{r}},(\textbf{g}_1 + \textbf{g}_2)\rangle ) }$ transforms $L^2_{\ell } \rightarrow L^2_{\ell +1}$. Thus, by conjugating $H_{{\textbf{k}}}(\alpha )$ by ${\mathscr {U}}$ we obtain unitarily equivalent operators $H_{{\textbf{k}}'}(\alpha )$ for some different ${\textbf{k}}'$ on other spaces $L^2_{\ell '}.$ In this sense, the choice of $L^2_{\ell }$ is arbitrary, up to a shift in $\textbf{k}$.

One then defines a magic angle of the chiral Hamiltonian as a parameter $\alpha \in {\mathbb {C}}$ such that

3.12 $$\begin{aligned} 0\in \bigcap _{\textbf{k}\in \mathbb {R}^2} {{\,\textrm{Spec}\,}}(H_{\textbf{k}}(\alpha )), \end{aligned}$$

where $H_{\textbf{k}}(\alpha )$ is defined as in (3.10).

For the Hamiltonian (3.10) the first 2 components correspond to lattice sites *A* and the remaining ones to lattice sites *B*. Since $D(\alpha )+(k_1+ik_2)\operatorname {id}_{\mathbb {C}^2}$ is a Fredholm operator of index 0, at a magic angle $\alpha$, the null space of the Hamiltonian decomposes into

3.13 $$\begin{aligned} \ker (H_{\textbf{k}}(\alpha )) = \ker (D(\alpha )+(k_1+ik_2)\operatorname {id}_{\mathbb {C}^2}) \oplus \ker (D(\alpha )^*+(k_1-ik_2)\operatorname {id}_{\mathbb {C}^2})\nonumber \\ \end{aligned}$$

where $\ker (D(\alpha )+(k_1+ik_2)\operatorname {id}_{\mathbb {C}^2})$ and $\ker (D(\alpha )^*+(k_1-ik_2)\operatorname {id}_{\mathbb {C}^2})$ are of equal dimension. Elements of $\ker (D(\alpha )+(k_1+ik_2)\operatorname {id}_{\mathbb {C}^2})$ therefore correspond to states at zero energy that are *A*-lattice polarized. This leads us to make the following definition

#### Definition 3.1

*(Multiplicity)*. A magic angle $\alpha$ is *n*-fold degenerate if the number of zero energy flat bands of the Hamiltonian is 2*n*-fold degenerate. A 1-fold degenerate magic angle is also called *simple*.

In the case of TBG and eTTG we indicate the number 2*M* of flat bands at a magic angle by appending a number 2*M*, e.g. TBG$-2$ for simple magic angles and TBG$-4$ for 2-fold degenerates ones.

The simplest one-parameter family of potentials satisfying (3.7) is, for $\varphi \in \mathbb {R}/\mathbb {Z}$, given by

$$\begin{aligned} U_{\varphi }(\textbf{r}) = \cos (2\pi \varphi ) \sum _{i=0}^2 \omega ^i e^{-i \textbf{q}_i\cdot \textbf{r}} + \sin (2\pi \varphi ) \sum _{i=0}^2\omega ^i e^{2i \textbf{q}_i \cdot \textbf{r}}.\end{aligned}$$

In twisted bilayer graphene, higher Fourier modes in the tunnelling potential are necessary to describe lattice relaxation effects, see e.g. [28, Remark 2.4] and references therein. We then consider special cases

3.14 $$\begin{aligned} U_0(\textbf{r}) = \sum _{i=0}^2 \omega ^i e^{-i \textbf{q}_i\cdot \textbf{r}} \text { and }U_{7/8}(\textbf{r}) =\frac{1}{\sqrt{2}}\Bigg ( U_0(\textbf{r}) - \sum _{i=0}^2\omega ^i e^{2i \textbf{q}_i \cdot \textbf{r}}\Bigg ). \end{aligned}$$

**Fig. 2:**
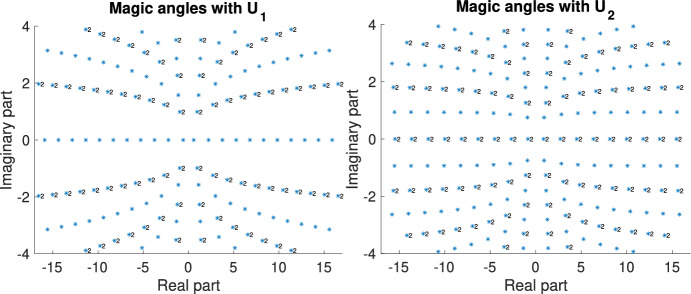
Magic angles $$\alpha $$ derived from potentials $$U=U_0$$ (left) and $$U = U_{7/8} $$ (right). The dimension of $$\operatorname {ker}(D(\alpha )+(k_1+ik_2)\operatorname {id}_{\mathbb {C}^2})$$ is indicated by the numbers in the figure. No number indicates a one-dimensional subspace. The number of flat bands of the Hamiltonian is twice the dimension of $$\operatorname {ker}(D(\alpha )+(k_1+ik_2)\operatorname {id}_{\mathbb {C}^2}).$$

While both $U_0$ and $U_{7/8}$ satisfy the above symmetries (3.4), (3.5), and (3.6), they give rise to a different number of flat bands for *magic* $\alpha \in \mathbb {R}$. Numerical experiments suggest, see Fig. 2, that in case of $U=U_0$ and $\alpha \in {\mathbb {R}}$ magic, precisely two bands of the chiral Hamiltonian become flat, in the case of $U=U_{7/8}$ and $\alpha \in \mathbb {R}$ the Hamiltonian exhibits four flat bands at *magic angles*. Thus, the chiral model with the two potentials $U=U_0$ and $U=U_{7/8}$ serve as models for TBG$-2$ and TBG$-4$. While simple and two-fold degenerate magic angles are the only types of magic angles that appear for generic choices of tunnelling potentials in chiral limit TBG, see [23, Theo. 3], they exhibit an almost equidistant spacing for the potentials $U_0$ and $U_{7/8}$, as shown in Table 1.

**Table 1:** First 11 real magic angles, rounded to 6 digits, for $U=U_0$ (left) and $U=U_{7/8}$ (right)

*k*	$\alpha _k$	$\alpha _{k}-\alpha _{k-1}$	*k*	$\alpha _k$	$\alpha _{k}-\alpha _{k-1}$
1	0.58566		1	0.853799	
2	2.22118	1.6355	2	2.691433	1.8376
3	3.75140	1.5302	3	4.507960	1.8165
4	5.27649	1.5251	4	6.332311	1.8244
5	6.79478	1.5183	5	8.157130	1.8248
6	8.31299	1.5182	6	9.983510	1.8264
7	9.82906	1.5161	7	11.809376	1.8259
8	11.34534	1.5163	8	13.635446	1.8261
9	12.86061	1.5153	9	15.460894	1.8255
10	14.37607	1.5155	10	17.286231	1.8253
11	15.89096	1.5149	11	19.111041	1.8248

### Magic angles in eTTG

When studying twisted trilayer graphene (TTG), the case of equal twisting angles (eTTG) is of particular interest.

In a recent paper by Popov and Tarnopolsky [31], the authors demonstrated that the collection of magic parameters ${\mathcal {A}}_{\textrm{eTTG}}$ exhibits a particularly interesting relation, which connects the set of magic angles of chiral limit TBG to the magic angles of equal twisting angle TTG (eTTG)

**Fig. 3:**
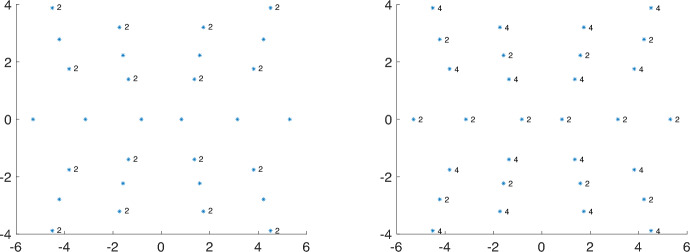
Magic angles of TBG multiplied by $\sqrt{2}$, $\sqrt{2}{\mathcal {A}}_{\text {TBG}}$ (left) and eTTG ${\mathcal {A}}_{\text {eTTG}}$ (right) with $U_+=U_0$, see (3.14). The multiplicity of the flat bands of the Hamiltonian is twice the multiplicity indicated in the figures

3.15 $$\begin{aligned} \sqrt{2}{\mathcal {A}}_{\textrm{TBG}-M}= {\mathcal {A}}_{\textrm{eTTG}-2M}, \end{aligned}$$

see also the top right figure in Fig. 3. Here ${\mathcal {A}}_{\textrm{eTTG}-2M}$ is the set of magic parameters for eTTG-2 M. Moreover, the multiplicity on the right is at least twice the one on the left. The argument provided in [31] constructs protected flat bands for eTTG-2 M from flat bands of TBG-M. This construction works for eTTG-2 M, however it does not extend to more than three layers nor does it seem to extend beyond the equal twisting angle case, as well.

At the center of this connection, is the map with obvious indices corresponding to $D(\alpha ) = D_{\text {TBG}}(\alpha )$ as in (3.2) and $D(\alpha ) = D_{\text {eTTG}}(\alpha )$ as in (3.3)

3.16 $$\begin{aligned} \times : \ker _{L^2_{k,j}}( D_{\text {TBG}}(\alpha _0))  &   \oplus \ker _{L^2_{k',j'}}(D_{\text {TBG}}(\alpha _0))\rightarrow \ker _{ L^2_{k+k',j+j'}}(D_{\text {eTTG}}(\sqrt{2}\alpha _0)) \nonumber \\ (v,w)  &   \mapsto (v_1w_1,2^{-1/2}(v_1w_2 + v_2 w_1), v_2w_2). \end{aligned}$$

This construction allows us to construct from any two elements of the nullspaces of $D_{\text {TBG}}$, for some magic $\alpha _0$, with respect to different representations, an element of the nullspace of $D_{\text {eTTG}},$ but for a rescaled magic parameter $\sqrt{2}\alpha _0.$ We observe that the number of zeros doubles by applying the map above. This means that the number of flat bands for eTTG, with $\sqrt{2}\alpha _0$ is (at least) twice the number of flat bands for TBG with $\alpha _0.$

### Periodicity of TBG and eTTG

As discussed previously, the Hamiltonians for TBG and eTTG as defined in the beginning of this section are not periodic with respect to $\Gamma$. However, by using the general form of the potentials $U_{\pm }(\textbf{r})$ (Eq. (3.7)), we see that we can turn TBG into a $\Gamma$-periodic Hamiltonian by conjugating it by $V(\textbf{r}):=\operatorname {diag}(1,e^{i \textbf{q}_1 \cdot \textbf{r}},1,e^{i \textbf{q}_1 \cdot \textbf{r}})$ to obtain the equivalent periodic Hamiltonian

$$\begin{aligned} {\mathscr {H}}(\alpha ) = \begin{bmatrix} 0 &  \mathscr {D}(\alpha )^{\dagger } \\ {\mathscr {D}}(\alpha ) &  0\end{bmatrix} \text { with }{\mathscr {D}}(\alpha ) = \begin{bmatrix}D_{x_1}+i D_{x_2} &  \alpha e^{i \textbf{q}_1 \cdot \textbf{r}} U_+(\textbf{r}) \\ \alpha e^{-i\textbf{q}_1 \cdot \textbf{r}} U_-(\textbf{r}) & D_{x_1}+i D_{x_2} + i \end{bmatrix}. \end{aligned}$$

Similarly, eTTG can be made periodic by conjugating by

$$\begin{aligned} V(\textbf{r}) = \operatorname {diag}(1,e^{i \textbf{q}_1 \cdot \textbf{r}}, e^{-i \textbf{q}_{1} \cdot \textbf{r}},1,e^{i \textbf{q}_1 \cdot \textbf{r}}, e^{-i \textbf{q}_{1} \cdot \textbf{r}}) \end{aligned}$$

and using the fact that $3 \textbf{q}_{1} = (0, 3)^{\top } \in \Gamma ^{*}$.

## Symmetries and Gauge Fixing

We now discuss the symmetry assumptions required for our main theorem which mirror the symmetries present in TBG and eTTG. Due to the form of the chiral Hamiltonian Eq. (3.1) and the symmetries of the $U_{\pm }(\textbf{r})$ (Eqs. (3.4), (3.5), (3.6)), the Hamiltonians for TBG and eTTG satisfy a number of symmetries in addition to the translation $\mathcal {T}_{\textbf{a}}$ and rotation $\mathcal {R}$. In particular, they satisfy a sublattice symmetry $\mathcal {Z}$, a layer symmetry $\mathcal {L}$, and a composite symmetry $\mathcal {Q}$, whose actions are defined as follows:

4.1 $$\begin{aligned} \mathcal {Z} \psi (\textbf{r}; \sigma , j)&= \sigma \psi (\textbf{r}; \sigma ,j) \end{aligned}$$

4.2 $$\begin{aligned} \mathcal {L} \psi (\textbf{r}; \sigma , j)&= (-1)^{j} \psi (-\textbf{r}; -\sigma , N-j). \end{aligned}$$

4.3 $$\begin{aligned} \mathcal {Q} \psi (\textbf{r}; \sigma , j)&= \overline{\psi (-\textbf{r}; -\sigma ,j)} \end{aligned}$$

In words, the sublattice symmetry multiplies the *A* sublattice by $+1$ and states supported on the *B* sublattice by $-1$. The layer symmetry $\mathcal {L}$, reverses the order of the layers, swaps the *A* and *B* sublattices, multiplies by an alternating minus sign, and maps $\textbf{r}\mapsto -\textbf{r}$. The symmetry $\mathcal {Q}$ is a composition of a $C_{2z}$ rotation and time reversal $\mathcal {T}$ and acts by swapping the *A* and *B* sublattices, taking a complex conjugate, and mapping $\textbf{r}\mapsto -\textbf{r}$. The composite symmetry $\mathcal {Q}$ is often referred to as the $C_{2z}\mathcal {T}$ symmetry. It is easily checked that $\{ \mathcal {Z}, \mathcal {Q} \} = 0$, $[ \mathcal {Z}, \mathcal {L} ] = 0$, and $[ \mathcal {Q}, \mathcal {L} ] = 0$.

We will use these symmetries to fix a specific choice of Bloch eigenbasis for the flat bands; a procedure we refer to as “gauge fixing” following the physics terminology.

### Assumption 3

*(Symmetry Assumptions)*. We assume that the single particle Hamiltonian *H* commutes with ${\mathcal {Q}}$ and anticommutes with ${\mathcal {Z}}$ and ${\mathcal {L}}$.

### Remark 2

Since we study the nullspace of *H*, we can consider both symmetries that commute or anticommute with *H* as both types of symmetries fix the nullspace.

If $\mathcal {Z}$, $\mathcal {Q}$, and $\mathcal {L}$ are symmetries of a periodic Hamiltonian, it is easily checked that $\mathcal {Z}$ and $\mathcal {Q}$ map $\textbf{k}$ to $\textbf{k}$ and $\mathcal {L}$ maps $\textbf{k}$ to $-\textbf{k}$.

Since the zero energy eigenstates are degenerate within the flat band, once we fix a basis of orthogonal flat-band eigenfunctions we may perform any *U*(2*M*) transformation to this basis and get an alternative choice of eigenbasis which spans the same space. Any such transformation takes the form of the mapping

$$\begin{aligned} \psi _{n\textbf{k}}(\textbf{r}) \mapsto \sum _{m \in \mathcal {N}} \psi _{m\textbf{k}}(\textbf{r}) [U(\textbf{k})]_{mn} \end{aligned}$$

where $U(\textbf{k})$ is a unitary matrix. Under this basis transformation, the creation and annihilation operators likewise transform as

$$\begin{aligned} {\hat{f}}^{\dagger }_{n{\textbf {k}}} \mapsto \sum _{m \in \mathcal {N}} {\hat{f}}^{\dagger }_{m{\textbf {k}}} [U({\textbf {k}})]_{mn} \qquad \qquad {\hat{f}}_{n{\textbf {k}}} \mapsto \sum _{m \in \mathcal {N}} {\hat{f}}_{m{\textbf {k}}} [\overline{U({\textbf {k}})}]_{mn} \end{aligned}$$

and similarly the form factor transforms as

$$\begin{aligned} \Lambda _{\textbf{k}}(\textbf{q}+ \textbf{G}) \mapsto U(\textbf{k})^{\dagger } \Lambda _{\textbf{k}}(\textbf{q}+ \textbf{G}) U(\textbf{k}+ \textbf{q}). \end{aligned}$$

By fixing a specific choice of eigenbasis, which we refer to as “fixing a gauge” following physics terminology, we can force the form factor to satisfy certain properties which will be a key part of our characterization of the Hartree-Fock ground state. While the matrix representation of the form factor can change under different gauge choices, certain properties (such as the singular values) do not change under the choice of gauge. In particular, we have the following non-degeneracy result.

### Lemma 4.1

For all $\textbf{k} \in {\mathcal {K}}$, $\Lambda _{\textbf{k}}(\textbf{0}) = I$ independent of the choice of gauge. Additionally, if the flat band spectral projector $\Pi (\textbf{k})$ is Lipschitz with Lipschitz constant *L* (Assumption 1), then for all $\textbf{k} \in \Omega ^{*}$

4.4 $$\begin{aligned} \Lambda _{\textbf{k}}(\textbf{q}') \Lambda _{\textbf{k}}(\textbf{q}')^{\dagger } \succeq \left( 1 - L |\textbf{q}'|\right) I. \end{aligned}$$

where *I* is the identity matrix and we recall that $A \succeq B$ means that $A - B$ is positive semidefinite.

### Proof

Proven in Sect. A. $\square$

This lemma also implies $\Lambda _{\textbf{k}}(\textbf{q}')^{\dagger } \Lambda _{\textbf{k}}(\textbf{q}') \succeq (1 - L | \textbf{q}' |) I$ since for any square matrix *A*, $A A^{\dagger }$ and $A^{\dagger } A$ are unitarily equivalent. Note that this lemma is only a non-trivial statement if $|\textbf{q}'| < L^{-1}$ since $\Lambda _{\textbf{k}}(\textbf{q}') \Lambda _{\textbf{k}}(\textbf{q}')^{\dagger }$ is always a positive semidefinite matrix.

Note that the FBI Hamiltonian only depends on the form factor $\Lambda _{\textbf{k}}(\textbf{q}+ \textbf{G})$ which in turn only depends on the periodic Bloch functions, $u_{n\textbf{k}}(\textbf{r}; \sigma , j)$. As such we will define our gauge fixing in terms of the periodic Bloch functions, $u_{n\textbf{k}}(\textbf{r}; \sigma , j)$, instead of the Bloch functions.

### Remark 3

When the Hamiltonian *H* exhibits band crossings at zero energy, the set $\{ u_{n\textbf{k}}(\textbf{r}): n \in \mathcal {N}, \textbf{k}\in \Omega ^{*} \}$ is still closed under the action of symmetry operations for $\textbf{k}\in \mathcal {K}_{\textrm{crossing}}$ since the basis at the crossing points is defined through continuity.

The fact the symmetries in Assumption 3 either commute or anti-commute implies that we can fix the gauge on the periodic Bloch functions so that they transform in a simple way under the action of these symmetries.

### [Style2 Style2]Proposition 4.2

Under Assumption 3, there exists a choice of gauge so that the periodic Bloch functions $u_{n \textbf{k}}(\textbf{r}; \sigma , j)$ satisfy the following relationships

4.5 $$\begin{aligned} {\mathcal {Z}} u_{n \textbf{k}}(\textbf{r}; \sigma , j)&= {\left\{ \begin{array}{ll} (+1) u_{n \textbf{k}}(\textbf{r}; \sigma ,j ) &  n > 0 \\ (-1) u_{n \textbf{k}}(\textbf{r}; \sigma ,j ) &  n < 0 \\ \end{array}\right. } \end{aligned}$$

4.6 $$\begin{aligned} {\mathcal {Q}} u_{n \textbf{k}}(\textbf{r}; \sigma , j)&= u_{(-n) \textbf{k}}(\textbf{r}; \sigma , j) \end{aligned}$$

4.7 $$\begin{aligned} {\mathcal {L}} u_{n \textbf{k}}(\textbf{r}; \sigma , j)&= u_{n (-\textbf{k})}(\textbf{r}; \sigma , j) \end{aligned}$$

Before proving Proposition 4.2, we first properly define the uniformly half-filled, translation invariant, ferromagnetic Slater determinant states. These states are defined based on the ${\mathcal {Z}}$ symmetry:

### Definition 4.3

*(Ferromagnetic Slater Determinants).* Suppose that the Bloch eigenbasis has been chosen so that it satisfies Eq. (4.5). We define two uniformly half-filled, translation invariant ferromagnetic Slater determinants, or ferromagnetic Slater determinants for short, to be many-body states of the form:

$$\begin{aligned} \vert \Psi _{+}\rangle = \prod _{\textbf{k}\in \mathcal {K}} \prod _{n > 0, n\in \mathcal {N}} {\hat{f}}_{n\textbf{k}}^{\dagger } \vert \textrm{vac}\rangle \qquad \vert \Psi _{-}\rangle = \prod _{\textbf{k}\in \mathcal {K}} \prod _{n < 0, n\in \mathcal {N}} {\hat{f}}_{n\textbf{k}}^{\dagger } \vert \textrm{vac}\rangle . \end{aligned}$$

That is, $\vert \Psi _{\pm }\rangle$ fully fills one of the two eigenspaces of $\mathcal {Z}$.

### Proof of Proposition 4.2

Since the zero energy eigenstates $\psi _{n\textbf{k}}(\textbf{r})$ are indexed by $n \in \mathcal {N}$ and $\mathcal {Z}$ is a symmetry of the Hamiltonian, we may change the gauge and relabel the eigenfunctions so that:

4.8 $$\begin{aligned} \begin{aligned} \mathcal {Z} u_{n\textbf{k}}(\textbf{r}; \sigma , j)&= (+1) u_{n\textbf{k}}(\textbf{r}; \sigma , j) \qquad n > 0, \\ \mathcal {Z} u_{n\textbf{k}}(\textbf{r}; \sigma , j)&= (-1) u_{n\textbf{k}}(\textbf{r}; \sigma , j) \qquad n < 0. \end{aligned} \end{aligned}$$

Since $\mathcal {Q}$ is a symmetry of the Hamiltonian and relates the two different sublattices, after fixing the Bloch eigenfunctions to satisfy Eq. (4.5), we may fix the span $\{ u_{n\textbf{k}}(\textbf{r}): n > 0\}$, and determine $n < 0$ by the sublattice symmetry $\mathcal {Q}$. That is, for all $n > 0$ we define

4.9 $$\begin{aligned} u_{(-n)\textbf{k}}(\textbf{r}; \sigma , j):= \mathcal {Q} u_{n\textbf{k}}(\textbf{r}; \sigma , j) = \overline{u_{n\textbf{k}}(-\textbf{r}; -\sigma , j)}. \end{aligned}$$

Additionally since $\mathcal {L}$ is also a symmetry of the Hamiltonian and relates $\textbf{k}$ and $-\textbf{k}$, after fixing the periodic Bloch functions to satisfy Eqs. (4.5), (4.6) we may fix the space $\{ u_{n\textbf{k}}(\textbf{r}): k_{1} \ge 0 \}$ and determine $k_{1} < 0$ by the layer symmetry $\mathcal {L}$. That is,

4.10 $$\begin{aligned} u_{n(-k_{1},k_{2})}(\textbf{r}; \sigma , j):= \mathcal {L} u_{n(k_{1},-k_{2})}(\textbf{r}; \sigma , j) = (-1)^{j} u_{n(k_{1}, -k_{2})}(-\textbf{r}; \sigma , N-j).\nonumber \\ \end{aligned}$$

This proves the result.

### Remark 4

We note that after fixing the gauge choice in Proposition 4.2, the map $\textbf{k} \mapsto u_{n \textbf{k}}$ may not be continuous and periodic in $\textbf{k}$. In fact, for TBG-2 it is known there is a topological obstruction which prevents construction of a continuous and periodic gauge satisfying ${\mathcal {Q}}$ [41]. Despite this issue, our proofs do not use any of smoothness properties of the gauge choice.

With this gauge fixing, we can now state the matrix representation of the form factor:

### Lemma 4.4

Suppose that the gauge has been chosen so that periodic Bloch functions $\{ u_{n\textbf{k}}(\textbf{r}): n \in \mathcal {N}, \textbf{k}\in \Omega ^{*} \}$ satisfy Eqs. (4.5), (4.6), (4.7). Then the form factor $\Lambda _{\textbf{k}}(\textbf{q}+ \textbf{G})$ can be written as

4.11 $$\begin{aligned} \Lambda _{\textbf{k}}(\textbf{q}+ \textbf{G}) = \begin{bmatrix} A_{\textbf{k}}(\textbf{q}+ \textbf{G}) &  \\ &  \overline{A_{\textbf{k}}(\textbf{q}+ \textbf{G})} \end{bmatrix} \end{aligned}$$

where

4.12 $$\begin{aligned} [A_{\textbf{k}}(\textbf{q}+ \textbf{G})]_{mn} = \frac{1}{| \Omega |} \sum _{\textbf{G}'} \sum _{\sigma ,j} \overline{{\hat{u}}_{m,\textbf{k}}(\textbf{G}'; \sigma , j)} {\hat{u}}_{n(\textbf{k}+\textbf{q})}(\textbf{G}+ \textbf{G}'; \sigma , j) \qquad m, n > 0.\nonumber \\ \end{aligned}$$

Let $\mathcal {K}$ be as in Eq. (2.13), then $A_{\textbf{k}}(\textbf{q}+ \textbf{G})$ additionally satisfies the following *sum rule*:

4.13 $$\begin{aligned} \sum _{\textbf{k}\in \mathcal {K}} \operatorname {Im}{{{\,\textrm{tr}\,}}{(A_{\textbf{k}}(\textbf{G}) )}} = 0, \quad \textbf{G}\in \Gamma ^{*}. \end{aligned}$$

### Proof

Recall the definition of the form factor

4.14 $$\begin{aligned} [\Lambda _{\textbf{k}}(\textbf{q}+ \textbf{G})]_{mn}:= \frac{1}{| \Omega |} \sum _{\textbf{G}' \in \Gamma ^*} \sum _{\sigma ,j}\overline{{\hat{u}}_{m\textbf{k}}(\textbf{G}'; \sigma , j)} {\hat{u}}_{n(\textbf{k}+ \textbf{q}+ \textbf{G})}(\textbf{G}'; \sigma , j). \end{aligned}$$

Since the definition of the form factor involves a sum over the sublattice $\sigma$ and $u_{n\textbf{k}}(\textbf{r})$ have disjoint sublattice support for $n > 0$ and $n < 0$ we immediately see that $[\Lambda _{\textbf{k}}(\textbf{q}+ \textbf{G})]_{mn} = 0$ if $m n < 0$ and hence $\Lambda _{\textbf{k}}(\textbf{q}+ \textbf{G})$ can be written as a block diagonal matrix.

Due to $\mathcal {Q}$ symmetry (Eq. (4.6)) we have that

4.15 $$\begin{aligned} \begin{aligned} {\hat{u}}_{(-n)\textbf{k}}(\textbf{G}; \sigma , j)&= \int _{\Omega } e^{-i \textbf{G}\cdot \textbf{r}} u_{(-n)\textbf{k}}(\textbf{r}; \sigma , j) \,\textrm{d}\textbf{r}\\&= \int _{\Omega } e^{-i \textbf{G}\cdot \textbf{r}} \overline{u_{n\textbf{k}}(-\textbf{r}; \sigma , j)} \,\textrm{d}\textbf{r}\\&= \overline{\int _{\Omega } e^{-i \textbf{G}\cdot \textbf{r}} u_{n\textbf{k}}(\textbf{r}; \sigma , j) \,\textrm{d}\textbf{r}} \\&= \overline{{\hat{u}}_{n\textbf{k}}(\textbf{G}; \sigma , j)}. \end{aligned} \end{aligned}$$

From this relation we easily see that

4.16 $$\begin{aligned} [\Lambda _{\textbf{k}}(\textbf{q}+ \textbf{G})]_{(-m)(-n)} = [\overline{\Lambda _{\textbf{k}}(\textbf{q}+ \textbf{G})}]_{mn} \end{aligned}$$

which together with the block diagonal structure implies Eq. (4.11). Now we turn to prove the sum rule Eq. (4.13).

Due to the layer symmetry (Eq. (4.7)) we have that

4.17 $$\begin{aligned} \begin{aligned} {\hat{u}}_{n(-\textbf{k})}(\textbf{G}; \sigma , j)&= \int _{\Omega } e^{-i \textbf{G}\cdot \textbf{r}} (-1)^{j} u_{n\textbf{k}}(-\textbf{r}; -\sigma , N - j) \,\textrm{d}\textbf{r}\\&= (-1)^{j} {\hat{u}}_{n\textbf{k}}(-\textbf{G}; -\sigma , N - j). \end{aligned} \end{aligned}$$

Recall that $\Gamma ^{*}$ is closed under the map $\textbf{G}\mapsto -\textbf{G}$ and therefore

4.18 $$\begin{aligned} \Lambda _{\textbf{k}}(\textbf{q}+ \textbf{G})&= \frac{1}{| \Omega |} \sum _{\textbf{G}' \in \Gamma ^*} \sum _{\sigma ,j}\overline{{\hat{u}}_{m\textbf{k}}(\textbf{G}'; \sigma , j)} {\hat{u}}_{n(\textbf{k}+ \textbf{q}+ \textbf{G})}(\textbf{G}'; \sigma , j) \nonumber \\&= \frac{1}{| \Omega |} \sum _{\textbf{G}' \in \Gamma ^*} \sum _{\sigma ,j}\overline{{\hat{u}}_{m(-\textbf{k})}(-\textbf{G}'; -\sigma , N - j)} {\hat{u}}_{n(-\textbf{k}- \textbf{q}- \textbf{G})}(-\textbf{G}'; -\sigma , N - j) \nonumber \\&= \Lambda _{-\textbf{k}}(-\textbf{q}- \textbf{G}) \end{aligned}$$

which for $\textbf{q}= \varvec{0}$ reduces to $\Lambda _{\textbf{k}}(\textbf{G}) = \Lambda _{-\textbf{k}}(-\textbf{G}) = \Lambda _{-\textbf{k}}(\textbf{G})^{\dagger }$ where the last equality is due to Eq. (2.21). Note that this immediately implies that $\Lambda _{\varvec{0}}(\textbf{G})$ is Hermitian.

We recall that elements of $\mathcal {K}$ can be written as $\textbf{k}=\frac{i}{n_{k_{x}}} \textbf{g}_{1} + \frac{j}{n_{k_{y}}} \textbf{g}_{2}$ for some $i \in [n_{k_{x}}]$ and $j \in [n_{k_{y}}]$. For any $\textbf{k}$ of this form, we can find a $\textbf{k}' \in \mathcal {K}$ and $\textbf{G}' \in \Gamma ^{*}$ so that $-\textbf{k}= \textbf{k}' + \textbf{G}'$. Therefore, we may partition the momentum grid $\mathcal {K}$ into three disjoint sets $\{ \varvec{0}\}$, $\mathcal {K}_{1}$ and $\mathcal {K}_{2}$. The point $\varvec{0}$ is the unique point so that $\textbf{k}= -\textbf{k}$ and $\mathcal {K}_{1}$ and $\mathcal {K}_{2}$ are defined so that for each $\textbf{k}\in \mathcal {K}_{1}$ there exists a $\textbf{k}' \in \mathcal {K}_{2}$ so that $\textbf{k}+ \textbf{k}' \in \Gamma ^{*}$. Since $A_{\textbf{k}}(\textbf{G}) = A_{\textbf{k}+ \textbf{G}'}(\textbf{G})$ for all $\textbf{G}' \in \Gamma ^{*}$ (Eq. (2.22)), we have

4.19 $$\begin{aligned} \begin{aligned} \sum _{\textbf{k}\in \mathcal {K}} A_{\textbf{k}}(\textbf{G})&= A_{\varvec{0}}(\textbf{G}) + \sum _{\textbf{k}\in \mathcal {K}_{1}} A_{\textbf{k}}(\textbf{G}) + \sum _{\textbf{k}\in \mathcal {K}_{2}} A_{\textbf{k}}(\textbf{G}) \\&= A_{\varvec{0}}(\textbf{G}) + \sum _{\textbf{k}\in \mathcal {K}_{1}} A_{\textbf{k}}(\textbf{G}) + \sum _{\textbf{k}\in \mathcal {K}_{1}} A_{-\textbf{k}}(\textbf{G}) \\&= A_{\varvec{0}}(\textbf{G}) + \sum _{\textbf{k}\in \mathcal {K}_{1}} (A_{\textbf{k}}(\textbf{G}) + A_{\textbf{k}}(\textbf{G})^{\dagger }) \\ \end{aligned} \end{aligned}$$

and hence $\sum _{\textbf{k}\in \mathcal {K}} \operatorname {Im}{ {{\,\textrm{tr}\,}}{( A_{\textbf{k}}(\textbf{G}))}} = 0$. Here we have used $A_{\varvec{0}}(\textbf{G})$ is Hermitian. $\square$

## Properties of the Flat-Band Interacting Hamiltonian

In this section, we start by reviewing the basics on Hartree-Fock theory for FBI Hamiltonians (Sect. 5.1) and then prove some of their important many-body properties. In particular, we prove that the FBI Hamiltonian (Eq. (2.25)) is positive semidefinite (Sect. 5.2) and therefore any state which has zero energy must be a ground state. Using this fact, along with the sum rule (Eq. (4.13)), we prove that the ferromagnetic Slater determiant state are ground states (Sect. 5.3). Finally, we show that the ferromagnetic Slater determinants are insulating in the sense of a charge gap, i.e., both adding and removing an electron costs a finite amount of energy (Sect. 5.4).

### A review of Hartree-Fock theory

Recall that Slater determinants defined by the set $\mathcal {S}$ in Eq. 1.5 take the form:

5.1 $$\begin{aligned} \vert \Psi _S\rangle = \prod _{i=1}^{M} \prod _{\textbf{k}\in \mathcal {K}} {\hat{b}}_{i\textbf{k}}^{\dag }\vert \textrm{vac}\rangle , \end{aligned}$$

where $\vert \textrm{vac}\rangle$ is the vacuum state, *M* is the half the number of bands, and

5.2 $$\begin{aligned} {\hat{b}}_{i\textbf{k}}^{\dag }=\sum _{n \in \mathcal {N}}{\hat{f}}_{n\textbf{k}}^{\dag } \Xi _{ni}(\textbf{k}) \qquad \sum _{n \in \mathcal {N}} \Xi _{ni}^*(\textbf{k}) \Xi _{nj}(\textbf{k}) = \delta _{ij} \end{aligned}$$

defines the creation operator for the Hartree-Fock orbitals for each $\textbf{k}\in \mathcal {K}$.

The Hartree-Fock equations can be expressed in terms of the one-body reduced density matrix (1-RDM). The 1-RDM associated with a given Slater determinant $\vert \Psi _S\rangle$ can be written as

5.3 $$\begin{aligned} [P(\textbf{k})]_{nm}=\langle \Psi _S|{\hat{f}}_{m\textbf{k}}^{\dagger }{\hat{f}}_{n\textbf{k}}|\Psi _S\rangle = \sum _{i=1}^{M} \Xi _{ni}(\textbf{k})\Xi _{mi}^*(\textbf{k}), \end{aligned}$$

Due to the orthogonality relation on $\Xi (\textbf{k})$ given in Eq. (5.2), we may verify that for each $\textbf{k}$ the 1-RDM $P(\textbf{k})$ is an orthogonal projection onto an *M*-dimensional vector space.

#### Remark 5

The specific matrix representation of a 1-RDM depends on the choice of basis used to define the creation and annihilation operators ${\hat{f}}_{m\textbf{k}}^\dagger$ and ${\hat{f}}_{m\textbf{k}}$. Using the gauge fixing scheme from Sect. 4, the two ferromagnetic Slater determinant states have 1-RDMs as follows (where $\eta \in \{ \pm 1 \}$):

5.4 $$\begin{aligned} \langle \Psi _{\eta } | {\hat{f}}_{m\textbf{k}}^{\dagger }{\hat{f}}_{n\textbf{k}} | \Psi _{\eta }\rangle = {\left\{ \begin{array}{ll} \delta _{mn} &  \eta m, \eta n > 0 \\ 0 &  \eta m< 0 \text { or } \eta n < 0 \end{array}\right. } \end{aligned}$$

That is, the 1-RDM for $\vert \Psi _{+}\rangle$ is identity for all $\textbf{k}$ and all for $m, n > 0$ and zero otherwise (and similarly for $\vert \Psi _{-}\rangle$).

Let us define the manifold of all admissible uniformly-filled 1-RDMs:

$$\begin{aligned} \mathcal {M}:= \{ P \in {\mathbb {C}}^{(2 M) \times (2M) }: P^2 = P,~ P^\dagger = P,~ {{\,\textrm{tr}\,}}{(P)} = M \}. \end{aligned}$$

Following the standard derivation of Hartree-Fock theory (see e.g., [42, 43]), the Hartree-Fock energy is given by finding the Slater determinant $\vert \Psi _{S}\rangle$ which minimizes the energy of the interacting Hamiltonian:

$$\begin{aligned} {\mathcal {E}}^{\mathrm{(HF)}} = \min _{\vert \Psi _S\rangle } \langle \Psi _S | {\hat{H}}_{\textrm{FBI}} | \Psi _S\rangle . \end{aligned}$$

Since ${\hat{H}}_{\textrm{FBI}}$ only involves the electron–electron interaction term, the Hartree-Fock energy can be written as the sum of two functionals acting on the 1-RDM $P \in \mathcal {M}^{\mathcal {K}}$. In particular,

5.5 $$\begin{aligned} {\mathcal {E}}^{\mathrm{(HF)}} = \min _{P \in \mathcal {M}^{\mathcal {K}}} \Big (J[P] + K[P] \Big ) \end{aligned}$$

where $J[\cdot ]$ and $K[\cdot ]$ are non-linear functionals in *P*, referred to as the Hartree and Fock energy functionals, respectively.

Due to the specific form of ${\hat{H}}_{\textrm{FBI}}$, using Wick’s theorem, the Hartree and Fock energies (up to a physically irrelevant constant) can be concisely written in terms the matrix $Q(\textbf{k}):= 2 P(\textbf{k}) - I$ as follows (see Sect. B):

5.6 $$\begin{aligned} J[P]&= \frac{1}{4|\Omega | N_{\textbf{k}}} \sum _{\textbf{G}\in \Gamma ^{*}} {\hat{V}}(\textbf{G}) \left| \sum _{\textbf{k}\in \mathcal {K}} {{\,\textrm{tr}\,}}{(\Lambda _{\textbf{k}}(\textbf{G}) Q(\textbf{k}))} \right| ^{2} \end{aligned}$$

5.7 $$\begin{aligned} K[P]&= -\frac{1}{4 | \Omega | N_{\textbf{k}}} \sum _{\textbf{k},\textbf{q}\in \mathcal {K}} \sum _{\textbf{G}\in \Gamma ^*} {\hat{V}}(\textbf{q}+ \textbf{G}) {{\,\textrm{tr}\,}}{\Big ( \Lambda _{\textbf{k}}(\textbf{q}+ \textbf{G}) Q(\textbf{k}+ \textbf{q}) \Lambda _{\textbf{k}}(\textbf{q}+ \textbf{G})^\dagger Q(\textbf{k})\Big ) }. \end{aligned}$$

### Positive semidefiniteness

In this section, we prove that the many-body Hamiltonian ${\hat{H}}_{\textrm{FBI}}$ is positive semidefinite. We start by proving the following

#### Lemma 5.1

For all $\textbf{q}' \in \mathbb {R}^{2}$, the operator ${\hat{\rho }}(\textbf{q}')$ satisfies ${\widehat{\rho }}(\textbf{q}')^{\dagger } = {\widehat{\rho }}(-\textbf{q}')$.

#### Proof

For any fixed $\textbf{q}\in \mathcal {K}$ and $\textbf{G}\in \Gamma ^{*}$, we calculate

5.8 $$\begin{aligned} \begin{aligned} {\widehat{\rho }}(\textbf{q}+ \textbf{G})^{\dagger }&= \sum _{\textbf{k}\in \mathcal {K}} \sum _{m,n \in \mathcal {N}} [\overline{\Lambda _{\textbf{k}}(\textbf{q}+ \textbf{G})}]_{mn} \left( {\hat{f}}_{n(\textbf{k}+\textbf{q})}^{\dagger } {\hat{f}}_{m\textbf{k}} - \frac{1}{2} \delta _{\textbf{q},0} \delta _{mn} \right) \\&= \sum _{\textbf{k}\in \mathcal {K}} \sum _{m,n \in \mathcal {N}} [\overline{\Lambda _{\textbf{k}}(\textbf{q}+ \textbf{G})}]_{nm} \left( {\hat{f}}_{m(\textbf{k}+\textbf{q})}^{\dagger } {\hat{f}}_{n\textbf{k}} - \frac{1}{2} \delta _{\textbf{q},0} \delta _{mn} \right) . \end{aligned} \end{aligned}$$

Now we would like to make the change of variables $\textbf{k}\mapsto \textbf{k}- \textbf{q}$. Unfortunately, in general $\textbf{k}, \textbf{q}\in \mathcal {K}$ does not imply that $\textbf{k}- \textbf{q}\in \mathcal {K}$. However, due to the definition of $\mathcal {K}$, we may always find a $\textbf{G}_{\textbf{k},\textbf{q}} \in \Gamma ^{*}$ so that

5.9 $$\begin{aligned} \textbf{k}- \textbf{q}= \widetilde{\textbf{k}- \textbf{q}} + \textbf{G}_{\textbf{k},\textbf{q}} \quad \text {where} \quad \widetilde{\textbf{k}- \textbf{q}} \in \mathcal {K}. \end{aligned}$$

Under the change of variables $\textbf{k}\mapsto \widetilde{\textbf{k}- \textbf{q}}$ we have that

5.10 $$\begin{aligned} \begin{aligned} {\widehat{\rho }}(\textbf{q}+ \textbf{G})^{\dagger }&= \sum _{\textbf{k}\in \mathcal {K}} \sum _{m,n \in \mathcal {N}} [\overline{\Lambda _{\textbf{k}- \textbf{q}- \textbf{G}_{\textbf{k},\textbf{q}}}(\textbf{q}+ \textbf{G})}]_{nm} \left( {\hat{f}}_{m(\textbf{k}+\textbf{G}_{\textbf{k},\textbf{q}})}^{\dagger } {\hat{f}}_{n(\textbf{k}- \textbf{q}+ \textbf{G}_{\textbf{k},\textbf{q}})} - \frac{1}{2} \delta _{\textbf{q},0} \delta _{mn} \right) \\&= \sum _{\textbf{k}\in \mathcal {K}} \sum _{m,n \in \mathcal {N}} [\overline{\Lambda _{\textbf{k}- \textbf{q}}(\textbf{q}+ \textbf{G})}]_{nm} \left( {\hat{f}}_{m\textbf{k}}^{\dagger } {\hat{f}}_{n(\textbf{k}- \textbf{q})} - \frac{1}{2} \delta _{\textbf{q},0} \delta _{mn} \right) \\&= \sum _{\textbf{k}\in \mathcal {K}} \sum _{m,n \in \mathcal {N}} [\Lambda _{\textbf{k}}(-\textbf{q}- \textbf{G})]_{mn} \left( {\hat{f}}_{m\textbf{k}}^{\dagger } {\hat{f}}_{n(\textbf{k}- \textbf{q})} - \frac{1}{2} \delta _{\textbf{q},0} \delta _{mn} \right) = {\widehat{\rho }}(- \textbf{q}- \textbf{G}) \end{aligned} \end{aligned}$$

where in the second line we have used Eqs. (2.15) and (2.22). $\square$

Since ${\hat{H}}_{\textrm{FBI}}$ takes the form

5.11 $$\begin{aligned} {\hat{H}}_{\mathrm {{FBI}}} = \frac{1}{N_{{\textbf {k}}} |\Omega |} \sum _{{\textbf {q}}'} {\hat{V}}({\textbf {q}}') {\widehat{\rho }}({\textbf {q}}') {\widehat{\rho }}(-{\textbf {q}}') = \frac{1}{N_{{\textbf {k}}} |\Omega |} \sum _{{\textbf {q}}'} {\hat{V}}({\textbf {q}}') {\widehat{\rho }}({\textbf {q}}') {\widehat{\rho }}({\textbf {q}}')^{\dag } \end{aligned}$$

and ${\hat{V}}({\textbf {q}}') > 0$, an immediate corollary is that *any* many-body state $\vert \Psi \rangle$ so that ${\hat{H}}_{\textrm{FBI}} \vert \Psi \rangle = 0$ must be a ground state.

### Ferromagnetic slater determinants are ground states

We can now check that the ferromagnetic Slater determinant states are ground states by verifying they are zero energy eigenstates.

#### [Style2 Style2]Proposition 5.2

(Proof of Result 1). Suppose that the single particle Hamiltonian *H* satisfies the symmetry assumption (Assumption 3). Then the two ferromagnetic Slater determinant states (Definition 4.3) are exact many-body ground states of the interacting model Eq. (2.25).

#### Proof

We show that for all $\textbf{q}' = \textbf{q}+ \textbf{G}$, ${\widehat{\rho }}(\textbf{q}') \vert \Psi _{\pm }\rangle = 0$ where $\vert \Psi _{\pm }\rangle$ is a ferromagnetic Slater determinant. While we only consider $\vert \Psi _{+}\rangle$, the calculation for $\vert \Psi _{-}\rangle$ follows similar steps.

5.12 $$\begin{aligned} \begin{aligned} {\widehat{\rho }}(\textbf{q}+ \textbf{G}) \vert \Psi _{+}\rangle&= \sum _{\textbf{k}\in \mathcal {K}} \left[ \sum _{m,n} [\Lambda _{\textbf{k}}(\textbf{q}+ \textbf{G})]_{mn} \left( {\hat{f}}_{m\textbf{k}}^\dagger {\hat{f}}_{n(\textbf{k}+\textbf{q})} - \frac{1}{2} \delta _{mn} \delta _{\textbf{q},\varvec{0}} \right) \right] \vert \Psi _{+}\rangle \\&= \left( \sum _{\textbf{k}\in \mathcal {K}} \sum _{m,n} [\Lambda _{\textbf{k}}(\textbf{q} + \textbf{G})]_{mn} {\hat{f}}_{m\textbf{k}}^\dagger {\hat{f}}_{n (\textbf{k}+ \textbf{q})} \right) \vert \Psi _{+}\rangle - \left( \frac{1}{2} \delta _{\textbf{q}, \varvec{0}} \sum _{\textbf{k} \in {\mathcal {K}}}  {{\,\textrm{tr}\,}}{(\Lambda _{\textbf{k}}(\textbf{G}))} \right) \vert \Psi _{+}\rangle \\&= \left( \sum _{\textbf{k}\in \mathcal {K}} \sum _{n > 0} [\Lambda _{\textbf{k}}(\textbf{q} + \textbf{G})]_{mn} \delta _{mn} \delta _{\textbf{q}, \textbf{0}} \right) \vert \Psi _{+}\rangle - \left( \frac{1}{2} \delta _{\textbf{q}, \varvec{0}} \sum _{\textbf{k} \in {\mathcal {K}}}  {{\,\textrm{tr}\,}}{(\Lambda _{\textbf{k}}(\textbf{G}))} \right) \vert \Psi _{+}\rangle \\&= \left( \delta _{\textbf{q}, \textbf{0}} \sum _{\textbf{k}\in \mathcal {K}} {{\,\textrm{tr}\,}}{(A_{\textbf{k}}(\textbf{G}))} \right) \vert \Psi _{+}\rangle - \left( \delta _{\textbf{q}, \varvec{0}} \sum _{\textbf{k} \in {\mathcal {K}}}  \operatorname {Re}({{\,\textrm{tr}\,}}{(A_{\textbf{k}}(\textbf{G}))}) \right) \vert \Psi _{+}\rangle \\&= \delta _{\textbf{q}, \varvec{0}} \left( \sum _{\textbf{k}\in \mathcal {K}} \operatorname {Im}{{\,\textrm{tr}\,}}(A_{\textbf{k}}(\textbf{G})) \right) \vert \Psi _{+}\rangle = 0. \end{aligned} \end{aligned}$$

To go from the second line to the third line, we used the following argument: Since $\vert \Psi _{+}\rangle$ only fills the $n > 0$ states, ${\hat{f}}_{m \textbf{k}}^{\dagger } {\hat{f}}_{n (\textbf{k} + \textbf{q})} \vert \Psi _{+}\rangle = 0$ unless $n > 0$. Due to the choice of gauge, $[\Lambda _{\textbf{k}}(\textbf{q} + \textbf{G})]_{mn} = 0$ if $m n < 0$, so

5.13 $$\begin{aligned} [\Lambda _{\textbf{k}}(\textbf{q} + \textbf{G})]_{mn} {\hat{f}}_{m\textbf{k}}^{\dagger } {\hat{f}}_{n(\textbf{k} + \textbf{q})} \vert \Psi _{+}\rangle = 0 \quad \text {unless n> 0 and m > 0.} \end{aligned}$$

Since all of $n > 0$ states are filled in $\vert \Psi _{+}\rangle$, the only way this term does not vanish is if $\textbf{q} = \textbf{0}$ and $m = n$. The last expression vanishes due to the sum rule in Eq. (4.13). $\square$

### Charge gap at the thermodynamic limit

As we will see, adding or removing an electron to a ferromagnetic Slater determinant always costs a non-zero amount of energy.

#### [Style2 Style2]Proposition 5.3

(Charge Gap of Ferromagnetic Slater Determinants). Fix $\eta \in \{ \pm 1 \}$ and consider an arbitrary single electron excitation of the ferromagnetic Slater determinant $\vert \Psi _{\eta }\rangle$. All such excitations can be written in terms of creation and annihilation operators of the following form:

5.14 $$\begin{aligned} {\hat{c}}^\dagger = \sum _{\textbf{k}\in \mathcal {K}} \sum _{\eta \ell < 0} {\hat{f}}^\dagger _{\ell \textbf{k}} c_{\ell \textbf{k}} \qquad {\hat{c}} = \sum _{\textbf{k}\in \mathcal {K}} \sum _{\eta \ell> 0} {\hat{f}}_{\ell \textbf{k}} \overline{c_{\ell \textbf{k}}} \qquad \sum _{\textbf{k}\in \mathcal {K}} \sum _{\eta \ell > 0} | c_{\ell ,\textbf{k}} |^2 = 1. \end{aligned}$$

where $\eta \ell < 0$ denotes summation over the set $\{ \ell : \eta \ell < 0 \}$ and similarly for $\eta \ell > 0$.

For such excitations, we have

5.15 $$\begin{aligned} \langle \Psi _{\eta } | {\hat{c}}^\dagger {\hat{H}}_{\text {FBI}} {\hat{c}} | \Psi _{\eta }\rangle&= \frac{1}{N_{{\textbf {k}}} | \Omega |} \sum _{{\textbf {q}}'} {\hat{V}}({\textbf {q}}') \sum _{{\textbf {k}}\in \mathcal {K}} \sum _{\eta \ell> 0} \sum _{\eta \ell ' > 0} c_{\ell '  {\textbf {k}}} [ \Lambda _{{\textbf {k}}}(-{\textbf {q}}') \Lambda _{{\textbf {k}}}(-{\textbf {q}}')^\dagger ]_{\ell , \ell ' } \overline{c_{\ell {\textbf {k}}}}, \nonumber \\ \langle \Psi _{\eta } | {\hat{c}} {\hat{H}}_{\text {FBI}} {\hat{c}}^\dagger | \Psi _{\eta }\rangle&= \frac{1}{N_{{\textbf {k}}} | \Omega |} \sum _{{\textbf {q}}'} {\hat{V}}({\textbf {q}}') \sum _{{\textbf {k}}\in \mathcal {K}} \sum _{\eta \ell< 0} \sum _{\eta \ell ' < 0} \overline{c_{\ell ' {\textbf {k}}}} [ \Lambda _{{\textbf {k}} + {\textbf {q}}'}(-{\textbf {q}}')^\dagger \Lambda _{{\textbf {k}} + {\textbf {q}}'}(-{\textbf {q}}')]_{\ell ' ,\ell } c_{\ell {\textbf {k}}}.\nonumber \\ \end{aligned}$$

An immediate corollary of Propositions 5.3, 4.1 is that the energy of these single excitation is strictly positive for any $N_{\textbf{k}}$:

#### Corollary 4

Let ${\mathcal {K}}$ be the Monkhorst-Pack grid Eq. (2.13) and suppose ${\hat{H}}_{\textrm{FBI}}$ satisfies Assumption 1. In the same setting as Proposition 5.3, there exists a constant $\alpha > 0$ independent of ${\mathcal {K}}$ so that

5.16 $$\begin{aligned} \langle \Psi _{\eta } | {\hat{c}}^\dagger {\hat{H}}_{\textrm{FBI}} {\hat{c}} | \Psi _{\eta }\rangle \ge \alpha \qquad \langle \Psi _{\eta } | {\hat{c}} {\hat{H}}_{\textrm{FBI}} {\hat{c}}^{\dagger } | \Psi _{\eta }\rangle \ge \alpha . \end{aligned}$$

#### Proof

We only prove the lower bound for $\langle \Psi _{\eta } | {\hat{c}}^\dagger {\hat{H}}_{\textrm{FBI}} {\hat{c}} | \Psi _{\eta }\rangle$, the second bound follows by essentially the same argument. Since $\Lambda _{\textbf{k}}(\textbf{0}) = I$ and $\sum _{\textbf{k}\in \mathcal {K}} \sum _{\eta \ell > 0} | c_{\ell ,\textbf{k}} |^2 = 1$, we always have the lower bound

5.17 $$\begin{aligned} \langle \Psi _{\eta } | {\hat{c}}^\dagger {\hat{H}}_{\textrm{FBI}} {\hat{c}} | \Psi _{\eta }\rangle \ge \frac{{\hat{V}}(\textbf{0})}{N_{\textbf{k}} | \Omega |} \end{aligned}$$

so the result is only non-trivial in the limit $N_{\textbf{k}} \rightarrow \infty$. By Lemma 4.1, we can find a radius *R* so that

5.18 $$\begin{aligned} \Lambda _{\textbf{k}}(\textbf{q}') \Lambda _{\textbf{k}}(\textbf{q}')^{\dagger } \succeq \frac{1}{2} \qquad \forall | \textbf{q}' | \le R. \end{aligned}$$

We also assume without loss of generality that *R* is chosen so that $R \le \frac{1}{2} \text {diam}{(\Omega ^{*})}$. Let’s define the set of points ${\mathcal {Q}}$

5.19 $$\begin{aligned} {\mathcal {Q}} = ({\mathcal {K}} + \Gamma ^{*}) \cap \Big \{ | \textbf{q}' | \le R \Big \}. \end{aligned}$$

Since the Monkhorst-Pack grid uses $N_{\textbf{k}}$ points to uniform sample $\Omega ^{*}$ and the set $\{ | \textbf{q}' | \le R \}$ fits within a single reciprocal unit cell, for all $N_{\textbf{k}}$ sufficiently large, the number of points contained in ${\mathcal {Q}}$ can be lower bounded by5.20Hence, we have the lower bound

5.21 $$\begin{aligned} \begin{aligned} \langle \Psi _{\eta } | {\hat{c}}^\dagger {\hat{H}}_{\text {FBI}} {\hat{c}} | \Psi _{\eta }\rangle&\ge \frac{1}{N_{{\textbf {k}}} | \Omega |} \sum _{{\textbf {q}}' \in {\mathcal {Q}}} {\hat{V}}({\textbf {q}}') \sum _{{\textbf {k}}\in \mathcal {K}} \sum _{\eta \ell> 0} \sum _{\eta \ell '> 0} c_{\ell '  {\textbf {k}}} [ \Lambda _{{\textbf {k}}}(-{\textbf {q}}') \Lambda _{{\textbf {k}}}(-{\textbf {q}}')^\dagger ]_{\ell ,\ell '} \overline{c_{\ell {\textbf {k}}}}, \\  &\ge \frac{1}{N_{{\textbf {k}}} | \Omega |} \sum _{{\textbf {q}}' \in {\mathcal {Q}}} {\hat{V}}({\textbf {q}}') \left( \frac{1}{2} \right) \sum _{{\textbf {k}}\in \mathcal {K}} \sum _{\eta \ell> 0} \sum _{\eta \ell ' > 0} \delta _{\ell ,\ell '} c_{\ell '  {\textbf {k}}} \overline{c_{\ell {\textbf {k}}}}, \\  &= \frac{1}{N_{{\textbf {k}}} | \Omega |} \sum _{{\textbf {q}}' \in {\mathcal {Q}}} {\hat{V}}({\textbf {q}}') \left( \frac{1}{2} \right) \\  &\ge \frac{1}{2 N_{{\textbf {k}}} | \Omega |} \left( \min _{|{\textbf {q}}'| \le R} {\hat{V}}({\textbf {q}}') \right) \# | {\mathcal {Q}} | \\  &\ge \frac{\pi R^{2}}{8}\left( \min _{|{\textbf {q}}'| \le R} {\hat{V}}({\textbf {q}}') \right) . \end{aligned} \end{aligned}$$

which proves the result. $\square$

We now proceed to the proof of Proposition 5.3

#### Proof

From the canonical anticommutation relations, we have that for all $\textbf{q}' = \textbf{q}+ \textbf{G}$

5.22 $$\begin{aligned} \begin{aligned} \,[ {\hat{f}}_{m{\textbf {k}}}^\dagger {\hat{f}}_{n({\textbf {k}}+ {\textbf {q}}')}, {\hat{f}}_{\ell ,{\textbf {k}}'}^{\dagger } ]&= \delta _{n\ell } \delta _{{\textbf {k}}',({\textbf {k}}+ {\textbf {q}}')} {\hat{f}}_{m{\textbf {k}}}^{\dagger }, \\ [ {\hat{f}}_{m{\textbf {k}}}^\dagger {\hat{f}}_{n({\textbf {k}}+ {\textbf {q}}')}, {\hat{f}}_{\ell ,{\textbf {k}}'} ]&= -\delta _{m\ell } \delta _{{\textbf {k}}',{\textbf {k}}} {\hat{f}}_{n({\textbf {k}}+{\textbf {q}}')}. \end{aligned} \end{aligned}$$

Therefore,

5.23 $$\begin{aligned} [ {\widehat{\rho }}({\textbf {q}}'), {\hat{f}}_{\ell ,{\textbf {k}}'}^\dagger ]&= \sum _{{\textbf {k}}} \sum _{mn} [\Lambda _{{\textbf {k}}}({\textbf {q}}')]_{mn} \delta _{n \ell } \delta _{{\textbf {k}}',({\textbf {k}}+ {\textbf {q}}')} {\hat{f}}_{m{\textbf {k}}}^\dagger , = \sum _{m} [\Lambda _{{\textbf {k}}' - {\textbf {q}}'}({\textbf {q}}')]_{m \ell } {\hat{f}}_{m ({\textbf {k}}'-{\textbf {q}}')}^\dagger ,\nonumber \\ [ {\widehat{\rho }}({\textbf {q}}'), {\hat{f}}_{\ell ,{\textbf {k}}'} ]&= -\sum _{{\textbf {k}}} \sum _{mn} [\Lambda _{{\textbf {k}}}({\textbf {q}}')]_{mn} \delta _{m \ell } \delta _{{\textbf {k}}',{\textbf {k}}} {\hat{f}}_{n({\textbf {k}}+ {\textbf {q}}')} = - \sum _{n} [\Lambda _{{\textbf {k}}'}({\textbf {q}}')]_{\ell n} {\hat{f}}_{n({\textbf {k}}'+{\textbf {q}}')}. \end{aligned}$$

Since the ferromagnetic Slater determinant state $\vert \Psi _{\eta }\rangle$ fully fills either the positive or negative bands, ${\hat{f}}_{\ell '\textbf{k}''} \vert \Psi _{\eta }\rangle \ne 0$ if and only if $\eta \ell ' > 0$. Therefore, for a fixed $\eta$, we only consider $\ell '$ so that $\eta \ell ' > 0$. For the ferromagnetic Slater determinant state $\vert \Psi _{\eta }\rangle$ for all $\textbf{q}'$ we have

5.24 $$\begin{aligned} \begin{aligned} \langle \Psi _{\eta }\vert&{\hat{f}}_{\ell \textbf{k}'}^\dagger {\widehat{\rho }}(\textbf{q}') {\widehat{\rho }}(-\textbf{q}') {\hat{f}}_{\ell '\textbf{k}''} \vert \Psi _{\eta }\rangle = - \langle \Psi _{\eta } | [ {\widehat{\rho }}(\textbf{q}'), {\hat{f}}_{\ell \textbf{k}'}^\dagger ] [{\widehat{\rho }}(-\textbf{q}'), {\hat{f}}_{\ell '\textbf{k}''} ] | \Psi _{\eta }\rangle \\&= \sum _{mn} [\Lambda _{\textbf{k}'-\textbf{q}'}(\textbf{q}')]_{m\ell } [\Lambda _{\textbf{k}''}(-\textbf{q}')]_{\ell 'n} \langle \Psi _{\eta } | {\hat{f}}_{m(\textbf{k}'-\textbf{q}')}^\dagger {\hat{f}}_{n(\textbf{k}''-\textbf{q}')} | \Psi _{\eta } \rangle \\&= \sum _{\eta m> 0} \sum _{\eta n > 0} [\Lambda _{\textbf{k}'-\textbf{q}'}(\textbf{q}')]_{m\ell } [\Lambda _{\textbf{k}'}(-\textbf{q}')]_{\ell 'n} \delta _{mn} \delta _{\textbf{k}'\textbf{k}''} \\&= [\Lambda _{\textbf{k}'}(-\textbf{q}') \Lambda _{\textbf{k}'}(-\textbf{q}')^\dagger ]_{\ell ',\ell } \delta _{\textbf{k}'\textbf{k}''} \end{aligned} \end{aligned}$$

where in the last line we have used the identity $\Lambda _{\textbf{k}- \textbf{q}'}(\textbf{q}') = \Lambda _{\textbf{k}}(-\textbf{q}')^\dagger$ Eq. (2.21).

Similar calculations show for $\eta \ell ' < 0$

5.25 $$\begin{aligned} \langle \Psi _{\eta }\vert {\hat{f}}_{\ell {\textbf {k}}'} {\widehat{\rho }}({\textbf {q}}') {\widehat{\rho }}({\textbf {q}}') {\hat{f}}_{\ell '  {\textbf {k}}''}^\dagger \vert \Psi _{\eta }\rangle = [\Lambda _{{\textbf {k}}' + {\textbf {q}}'}(-{\textbf {q}}')^\dagger \Lambda _{{\textbf {k}}' + {\textbf {q}}'}(-{\textbf {q}}')]_{\ell ,\ell '} \delta _{{\textbf {k}}'  {\textbf {k}}''}.\end{aligned}$$

Therefore, by linearity

5.26 $$\begin{aligned} \begin{aligned} \langle \Psi _{\eta } | {\hat{c}}^\dagger {\widehat{\rho }}({\textbf {q}}') {\widehat{\rho }}(-{\textbf {q}}') {\hat{c}} | \Psi _{\eta }\rangle&= \sum _{{\textbf {k}}\in \mathcal {K}} \sum _{\eta \ell> 0} \sum _{\eta \ell ' > 0} c_{\ell '  {\textbf {k}}} [ \Lambda _{{\textbf {k}}}(-{\textbf {q}}') \Lambda _{{\textbf {k}}}(-{\textbf {q}}')^\dagger ]_{\ell ,\ell '} \overline{c_{\ell {\textbf {k}}}} \\ \langle \Psi _{\eta } | {\hat{c}} {\widehat{\rho }}({\textbf {q}}') {\widehat{\rho }}(-{\textbf {q}}') {\hat{c}}^\dagger | \Psi _{\eta }\rangle&= \sum _{{\textbf {k}}\in \mathcal {K}} \sum _{\eta \ell< 0} \sum _{\eta \ell ' < 0} \overline{c_{\ell '  {\textbf {k}}}} [ \Lambda _{{\textbf {k}} + {\textbf {q}}'}(-{\textbf {q}}')^\dagger \Lambda _{{\textbf {k}} + {\textbf {q}}'}(-{\textbf {q}}')]_{\ell ' ,\ell } c_{\ell {\textbf {k}}} \end{aligned} \end{aligned}$$

which implies the proposition. $\square$

## The Hartree-Fock Ground States of the Flat-Band Interacting Hamiltonian

### Rigorous statement of result 2

We can now state our main result rigorously:

#### [Style2 Style2]Theorem 5

(Main theorem). Suppose that the single particle Hamiltonian *H* satisfies Assumptions 1, 3. Suppose further that the Monkhorst-Pack grid $\mathcal {K}$ has been chosen to satisfy Assumption 2. If there exists a $\textbf{k}\in \mathcal {K}$ so that

1. For some $$\textbf{G}$$, $$\operatorname {Im}{{\,\textrm{tr}\,}}{(A_{\textbf{k}}(\textbf{G}))} \ne 0$$
2. For all non-trivial orthogonal projectors $\Pi$ (i.e. $\Pi$ is not zero or identity), there exists a $\textbf{G}'$ so that
  6.1 $$\begin{aligned} \Vert (I - \Pi ) A_{\textbf{k}}(\textbf{G}') \Pi \Vert > 0 \end{aligned}$$

then the two ferromagnetic Slater determinants are the unique translation-invariant Hartree-Fock ground states of ${\hat{H}}_{\textrm{FBI}}$ in  Eq. (2.25).

The conditions in Theorem 5 involves checking all orthogonal projectors and are difficult to verify computationally. For the special case of 2 and 4 flat bands, these conditions can be significantly simplified.

#### Corollary 6

(Two Band Case). Suppose that the single particle Hamiltonian *H* satisfies Assumptions 1, 3, and has two flat bands. Suppose further that the Monkhorst-Pack grid $\mathcal {K}$ has been chosen to satisfy Assumption 2. If there exists a $\textbf{k}\in \mathcal {K}$ and $\textbf{G}$ so that $\operatorname {Im}(A_{\textbf{k}}(\textbf{G})) \ne 0$ then the two ferromagnetic Slater determinants are the unique translation-invariant Hartree-Fock ground states of Eq. (2.25).

#### Proof

This follows immediately from Theorem 5 since in this case $A_{\textbf{k}}(\textbf{G})$ is a scalar ($1 \times 1$ matrix) and there are no nontrivial orthogonal projectors for scalars. $\square$

#### Corollary 7

(Four Band Case). Suppose that the single particle Hamiltonian *H* satisfies Assumptions 1, 3, and has four flat bands. Suppose further that the Monkhorst-Pack grid $\mathcal {K}$ has been chosen to satisfy Assumption 2. If there exists a $\textbf{k}\in \mathcal {K}$ so that

1. For some $$\textbf{G}$$, $$\operatorname {Im}{{\,\textrm{tr}\,}}{(A_{\textbf{k}}(\textbf{G}))} \ne 0$$,
2. For some $$\textbf{G}', \textbf{G}''$$, $$[A_{\textbf{k}}(\textbf{G}'), A_{\textbf{k}}(\textbf{G}'')] \ne 0,$$

then the two ferromagnetic Slater determinants are the unique translation-invariant Hartree-Fock ground states of Eq. (2.25).

#### Proof

The first condition is the same as Theorem 5 so we only need to show the commutator condition implies the condition on projectors.

Since for four bands, $A_{\textbf{k}}(\textbf{G})$ is a $2 \times 2$ matrix, the only non-trivial projectors are rank one projectors. Therefore, we only need to show that for all $\vert v\rangle \in {\mathbb {C}}^{2}$ with $\Vert v \Vert = 1$, there exists a $\textbf{G}$ so that $\langle v^{\perp } | A_{\textbf{k}}(\textbf{G}) | v\rangle \ne 0$ where $\vert v^{\perp }\rangle$ is a unit vector orthogonal to $\vert v\rangle$.

Suppose that $\textbf{G}', \textbf{G}''$ are so that $[A_{\textbf{k}}(\textbf{G}'), A_{\textbf{k}}(\textbf{G}'')] \ne 0$ and pick some $\vert v\rangle \in {\mathbb {C}}^{2}$. We have three cases:

Case 1: $$\langle v^{\perp } | A_{\textbf{k}}(\textbf{G}') | v\rangle \ne 0$$ Take $$\textbf{G}= \textbf{G}'$$
Case 2: $\langle v^{\perp } | A_{\textbf{k}}(\textbf{G}') | v\rangle = 0$ but $\langle v | A_{\textbf{k}}(\textbf{G}') | v^{\perp }\rangle \ne 0$ Observe that
  6.2 $$\begin{aligned} \overline{\langle v | A_{\textbf{k}}(\textbf{G}') | v^{\perp }\rangle } = \langle v^{\perp } | A_{\textbf{k}}(\textbf{G}')^{\dagger } | v\rangle \ne 0 \end{aligned}$$
  but by Eq. (2.21) $A_{\textbf{k}}(\textbf{G}')^{\dagger } = A_{\textbf{k}}(-\textbf{G}')$ so we take $\textbf{G}= -\textbf{G}'.$
Case 3: $\langle v^{\perp } | A_{\textbf{k}}(\textbf{G}') | v\rangle = 0$ and $\langle v | A_{\textbf{k}}(\textbf{G}') | v^{\perp }\rangle = 0$ Since $\vert v\rangle$ and $\vert v^{\perp }\rangle$ are an orthogonal basis for ${\mathbb {C}}^{2}$, the assumptions in this case implies that $\vert v\rangle$ and $\vert v^{\perp }\rangle$ are eigenvectors of $A_{\textbf{k}}(\textbf{G}')$. Therefore, we can orthogonally diagonalize $A_{\textbf{k}}(\textbf{G}')$ as $A_{\textbf{k}}(\textbf{G}') = V \Sigma V^{\dagger }$ where *V* is an orthogonal matrix whose columns are $\vert v\rangle$, $\vert v^{\perp }\rangle$ and $\Sigma$ is a diagonal matrix of eigenvalues. Since $[A_{\textbf{k}}(\textbf{G}'), A_{\textbf{k}}(\textbf{G}'')] \ne 0$, using the eigendecomposition of $A_{\textbf{k}}(\textbf{G}')$ we have that $V \Sigma V^{\dagger } A_{\textbf{k}}(\textbf{G}'') - A_{\textbf{k}}(\textbf{G}'') V \Sigma V^{\dagger } \ne 0$ which implies
  6.3 $$\begin{aligned} \Sigma \Big (V^{\dagger } A_{\textbf{k}}(\textbf{G}'') V \big ) - \Big (V^{\dagger } A_{\textbf{k}}(\textbf{G}'') V \Big ) \Sigma \ne 0 \end{aligned}$$
  If $V^{\dagger } A_{\textbf{k}}(\textbf{G}'') V$ were a diagonal matrix then Eq. (6.3) would be zero so it must be that either $\langle v^{\perp } | A_{\textbf{k}}(\textbf{G}'') | v\rangle \ne 0$ or $\langle v | A_{\textbf{k}}(\textbf{G}'') | v^{\perp }\rangle \ne 0$. If $\langle v^{\perp } | A_{\textbf{k}}(\textbf{G}'') | v\rangle \ne 0$ we can take $\textbf{G}= \textbf{G}''$. If $\langle v | A_{\textbf{k}}(\textbf{G}'') | v^{\perp }\rangle \ne 0$, appealing to Eq. (2.21), we can take $\textbf{G}= -\textbf{G}''$.

$$\square $$

## Proof of Theorem 5

As we saw in Sect. 4, with a proper choice of gauge, the sublattice symmetry $\mathcal {Z}$ implies we can partition the set of flat bands $\mathcal {N}$ into two sets $n > 0$ and $n < 0$ whose basis functions are supported on the *A* or *B* sublattices respectively. The composite symmetry $\mathcal {Q}$ further implies these two sets are of equal size and are related by an antiunitary transformation.

To make use of this observation, suppose that we have a 1-RDM $P(\textbf{k})$ for a model with 2*M* flat bands. If this state is uniformly half-filled, then for each $\textbf{k}\in \mathcal {K}$, $P(\textbf{k})$ can be expressed as a $(2 M) \times (2 M)$ projection matrix with rank *M*. Hence, we can write $P(\textbf{k}) = \Phi (\textbf{k}) \Phi (\textbf{k})^{\dagger }$ where $\Phi (\textbf{k})$ is a $(2 M) \times M$ matrix with orthogonal columns.

Since $\Phi (\textbf{k})$ has orthogonal columns, we may apply the cosine-sine (CS) decomposition [44] to decompose $\Phi (\textbf{k})$ so that it respects the decomposition into $n > 0$ and $n < 0$:

7.1 $$\begin{aligned} \Phi (\textbf{k}) = \begin{bmatrix} U_1(\textbf{k}) &  \\ &  U_2(\textbf{k}) \end{bmatrix} \begin{bmatrix} {\tilde{c}}(\textbf{k}) &  -{\tilde{s}}(\textbf{k}) \\ {\tilde{s}}(\textbf{k}) &  {\tilde{c}}(\textbf{k}) \end{bmatrix} \begin{bmatrix} V(\textbf{k})^\dagger \\ 0 \end{bmatrix} \end{aligned}$$

where

7.2 $$\begin{aligned} \begin{aligned}&{\tilde{c}}(\textbf{k}) = \operatorname {diag}( \cos {(\theta _1(\textbf{k}) / 2)}, \cos {(\theta _2(\textbf{k}) / 2)}, \cdots ,\cos {(\theta _{M}(\textbf{k}) / 2)}) \\&{\tilde{s}}(\textbf{k}) = \operatorname {diag}( \sin {(\theta _1(\textbf{k}) / 2)}, \sin {(\theta _2(\textbf{k}) / 2)}, \cdots , \sin {(\theta _{M}(\textbf{k}) / 2)}) \end{aligned} \end{aligned}$$

and $U_1(\textbf{k}), U_2(\textbf{k}), V(\textbf{k})$ are $M \times M$ unitary matrices.

Using this decomposition for $\Phi (\textbf{k})$, we can express the Hartree-Fock energy (Eqs. (5.6), (5.7)) in terms of the quantities $\theta _{i}(\textbf{k}), U_{1}(\textbf{k}), U_{2}(\textbf{k})$ and the blocks of the form factor $A_{\textbf{k}}(\textbf{q}+ \textbf{G})$ (see Lemma 4.4 for the definition of $A_{\textbf{k}}(\textbf{q}+ \textbf{G})$).

Since the Hartree-Fock energy is written in terms of the matrix $Q(\textbf{k})$, we begin writing $Q(\textbf{k})$ in terms of the CS decomposition Eq. (7.1). By definition we have

7.3 $$\begin{aligned} \begin{aligned} P(\textbf{k})&= \Phi (\textbf{k}) \Phi (\textbf{k})^\dagger \\&= \begin{bmatrix} U_1(\textbf{k}) &  \\ &  U_2(\textbf{k}) \end{bmatrix} \begin{bmatrix} {\tilde{c}}(\textbf{k})^2 &  {\tilde{c}}(\textbf{k}){\tilde{s}}(\textbf{k}) \\ {\tilde{c}}(\textbf{k}){\tilde{s}}(\textbf{k}) &  {\tilde{s}}(\textbf{k})^2 \end{bmatrix} \begin{bmatrix} U_1(\textbf{k})^\dagger &  \\ &  U_2(\textbf{k})^\dagger \end{bmatrix}. \end{aligned} \end{aligned}$$

Since $Q(\textbf{k}) = 2 P(\textbf{k}) - I$, we have

7.4 $$\begin{aligned} Q(\textbf{k}) = \begin{bmatrix} U_1(\textbf{k}) &  \\ &  U_2(\textbf{k}) \end{bmatrix} \begin{bmatrix} c(\textbf{k}) &  s(\textbf{k}) \\ s(\textbf{k}) &  -c(\textbf{k}) \end{bmatrix} \begin{bmatrix} U_1(\textbf{k})^\dagger &  \\ &  U_2(\textbf{k})^\dagger \end{bmatrix} \end{aligned}$$

where

7.5 $$\begin{aligned} \begin{aligned}&c(\textbf{k}) = \operatorname {diag}( \cos {(\theta _1(\textbf{k}))}, \cos {(\theta _2(\textbf{k}))}, \cdots , \cos {(\theta _{M}(\textbf{k}))}) \\&s(\textbf{k}) = \operatorname {diag}( \sin {(\theta _1(\textbf{k}))}, \sin {(\theta _2(\textbf{k}))}, \cdots ,\sin {(\theta _{M}(\textbf{k}))}. \end{aligned} \end{aligned}$$

Due to our decomposition of $\Phi (\textbf{k})$, $P(\textbf{k})$ is a ferromagnetic Slater determinant if and only if one of the following holds:

- For all $i \in \{ 1, \cdots , M\}$ and all $\textbf{k}$, $\theta _{i}(\textbf{k}) = 0$, or
- For all $i \in \{ 1, \cdots , M\}$ and all $\textbf{k}$, $\theta _{i}(\textbf{k}) = \pi$

Hence the ferromagnetic Slater states correspond to having $c(\textbf{k}) = \pm I$.

To prove uniqueness of the ferromagnetic Slater determinant states, we will show that they are the unique states in $\mathcal {S}$ which achieve the minimum value for both the Hartree and the Fock energies *simultaneously*. We will first show that ferromagnetic Slater determinants are minimizers of the Hartree energy in Sect. 7.1. Then in Sect. 7.2, we will show that the assumptions of Theorem 5 imply that the ferromagnetic Slater determinant is the unique minimizer of the Fock energy.

### Minimizing the Hartree energy

We recall the expression for the Hartree energy

7.6 $$\begin{aligned} J[P] = \frac{1}{4|\Omega | N_{\textbf{k}}} \sum _{\textbf{G}} {\hat{V}}(\textbf{G}) \left| \sum _{\textbf{k}\in \mathcal {K}} {{\,\textrm{tr}\,}}{(\Lambda _{\textbf{k}}(\textbf{G}) Q(\textbf{k}))} \right| ^{2}. \end{aligned}$$

Note that since ${\hat{V}}(\textbf{G}) > 0$, necessarily $J[P] \ge 0$.

Now recall that, due to the symmetry assumptions on the form factor (Lemma 4.4) we can write

7.7 $$\begin{aligned} \Lambda _{\textbf{k}}(\textbf{q}+ \textbf{G}) = \begin{bmatrix} A_{\textbf{k}}(\textbf{q}+ \textbf{G}) &  \\ &  \overline{A_{\textbf{k}}(\textbf{q}+ \textbf{G})} \end{bmatrix}. \end{aligned}$$

Since we will multiply the form factor by $Q(\textbf{k})$, it will be convenient to define

7.8 $$\begin{aligned} \begin{aligned} B^{(1)}_{\textbf{k}}(\textbf{G})&= U_{1}(\textbf{k}) A_{\textbf{k}}(\textbf{G}) U_{1}(\textbf{k})^{\dagger } \\ B^{(2)}_{\textbf{k}}(\textbf{G})&= U_{2}(\textbf{k}) \overline{A_{\textbf{k}}(\textbf{G})} U_{2}(\textbf{k})^{\dagger }. \end{aligned} \end{aligned}$$

The trace in the Hartree energy can then be written in terms of $B^{(1)}_{\textbf{k}}(\textbf{G})$ and $B^{(2)}_{\textbf{k}}(\textbf{G})$ as follows:

7.9 $$\begin{aligned} \begin{aligned} {{\,\textrm{tr}\,}}{(\Lambda _{\textbf{k}}(\textbf{G}) Q(\textbf{k}) )}&= {{\,\textrm{tr}\,}}{\Big ( \begin{bmatrix} B^{(1)}_{\textbf{k}}(\textbf{G}) &  \\ &  B^{(2)}_{\textbf{k}}(\textbf{G}) \end{bmatrix} \begin{bmatrix} c(\textbf{k}) &  s(\textbf{k}) \\ s(\textbf{k}) &  -c(\textbf{k}) \end{bmatrix} \Big )} \\&= {{\,\textrm{tr}\,}}{\Big ( (B^{(1)}_{\textbf{k}}(\textbf{G}) - B^{(2)}_{\textbf{k}}(\textbf{G}) ) c(\textbf{k}) \Big )}. \end{aligned} \end{aligned}$$

For the ferromagnetic Slater determinant state, $c(\textbf{k}) = \pm I$ and hence

7.10 $$\begin{aligned}  &   {{\,\textrm{tr}\,}}{(\Lambda _{\textbf{k}}(\textbf{G}) Q(\textbf{k}) )} = \pm {{\,\textrm{tr}\,}}{\Big ( (B^{(1)}_{\textbf{k}}(\textbf{G}) - B^{(2)}_{\textbf{k}}(\textbf{G}) ) \Big )} = \pm 2 i \operatorname {Im}{{{\,\textrm{tr}\,}}{( A_{\textbf{k}}(\textbf{G}) )}} \end{aligned}$$

7.11 $$\begin{aligned}  &   J(P) = \frac{1}{2 | \Omega | N_{\textbf{k}}} \sum _{\textbf{G}} V(\textbf{G}) \left| \sum _{\textbf{k}} \operatorname {Im}{{{\,\textrm{tr}\,}}{( A_{\textbf{k}}(\textbf{G}) )}} \right| ^{2} = 0 \end{aligned}$$

where the last equality is due to the sum rule Eq. (4.13). Therefore, the ferromagnetic Slater determinants minimize the Hartree energy.

### Minimizing the Fock energy

For these calculations, we will adopt the shorthand $\textbf{k}':= \textbf{k}+ \textbf{q}$ and $\textbf{q}' = \textbf{q}+ \textbf{G}$ and generalize the definitions of $B^{(1)}_{\textbf{k}}(\textbf{G})$ and $B^{(2)}_{\textbf{k}}(\textbf{G})$ from the previous section:

7.12 $$\begin{aligned} \begin{aligned} B^{(1)}_{\textbf{k}}(\textbf{q}+ \textbf{G})&= B^{(1)}_{\textbf{k}}(\textbf{q}') = U_{1}(\textbf{k}) A_{\textbf{k}}(\textbf{q}') U_{1}(\textbf{k}')^{\dagger } \\ B^{(2)}_{\textbf{k}}(\textbf{q}+ \textbf{G})&= B^{(2)}_{\textbf{k}}(\textbf{q}') = U_{2}(\textbf{k}) \overline{A_{\textbf{k}}(\textbf{q}')} U_{2}(\textbf{k}')^{\dagger }. \end{aligned} \end{aligned}$$

After some lengthy computations (Sect. C) it can be shown that

7.13 $$\begin{aligned} \begin{aligned} K[P] = -\frac{1}{8 | \Omega | N_{\textbf{k}}} \sum _{\textbf{q}'} V(\textbf{q}') \sum _{\textbf{k}}  \sum _{ij} \bigg \{&| [B^{(1)}_{\textbf{k}}(\textbf{q}') + B^{(2)}_{\textbf{k}}(\textbf{q}')]_{ij} |^2 \cos {(\theta _i(\textbf{k}) - \theta _j(\textbf{k}'))} \\&+ | [B^{(1)}_{\textbf{k}}(\textbf{q}') - B^{(2)}_{\textbf{k}}(\textbf{q}')]_{ij} |^2 \cos {(\theta _i(\textbf{k}) + \theta _j(\textbf{k}'))} \bigg \}. \end{aligned} \end{aligned}$$

We will take a closer look at the two terms in the parenthesis given in  Eq. (7.13) by

7.14 $$\begin{aligned} \begin{aligned} |&[B^{(1)}_{\textbf{k}}(\textbf{q}') + B^{(2)}_{\textbf{k}}(\textbf{q}')]_{ij} |^2 \cos {(\theta _i(\textbf{k}) - \theta _j(\textbf{k}'))} \text { and }\\ |&[B^{(1)}_{\textbf{k}}(\textbf{q}') - B^{(2)}_{\textbf{k}}(\textbf{q}')]_{ij} |^2 \cos {(\theta _i(\textbf{k}) + \theta _j(\textbf{k}'))}. \end{aligned} \end{aligned}$$

From Eq. (7.14), we can understand the fundamental mechanism which forces the ground state to be a ferromagnetic Slater determinant. Since ${\hat{V}}(\textbf{q}') > 0$, if we assume that the first factors in (7.14) are non-zero

7.15 $$\begin{aligned} | [B^{(1)}_{\textbf{k}}(\textbf{q}') + B^{(2)}_{\textbf{k}}(\textbf{q}')]_{ij} |> 0 \qquad | [B^{(1)}_{\textbf{k}}(\textbf{q}') - B^{(2)}_{\textbf{k}}(\textbf{q}')]_{ij} | > 0 \end{aligned}$$

then for a state to minimize the Fock energy it must be that

7.16 $$\begin{aligned} \begin{aligned} \cos {(\theta _{i}(\textbf{k}) - \theta _{j}(\textbf{k}') )} = 1&\Rightarrow \theta _{i}(\textbf{k}) = \theta _{j}(\textbf{k}') \pmod {2 \pi } \\ \cos {(\theta _{i}(\textbf{k}) + \theta _{j}(\textbf{k}') )} = 1&\Rightarrow \theta _{i}(\textbf{k}) = -\theta _{j}(\textbf{k}') \pmod {2 \pi }. \end{aligned} \end{aligned}$$

If this weren’t the case then we could decrease the energy further.

The first constraint forces $\theta _{i}(\textbf{k})$ to be constant (independent of *i* and $\textbf{k}$) and the second constraint forces $\theta _{i}(\textbf{k}) \in \{ 0, \pi \}$. These two facts combined show that the ferromagnetic Slater determinants minimize the Fock energy and suggest a strategy for proving these states are the unique Hartree-Fock minimizers.

By definition

$$\begin{aligned} [B^{(1)}_{\textbf{k}}(\textbf{q}') \pm B^{(2)}_{\textbf{k}}(\textbf{q}')]_{ij} = [U_{1}(\textbf{k}) A_{\textbf{k}}(\textbf{q}') U_{1}(\textbf{k}')^{\dagger } \pm U_{2}(\textbf{k}) \overline{A_{\textbf{k}}(\textbf{q}')} U_{2}(\textbf{k}')^{\dagger }]_{ij}.\end{aligned}$$

While generically, it may be true that the above quantity does not vanish, since $U_{1}(\textbf{k})$ and $U_{2}(\textbf{k})$ are arbitrary unitaries, for any *i*, *j* we can always find specific choices of $U_{1}(\textbf{k})$, $U_{2}(\textbf{k})$ so that the above vanishes. The assumptions of Theorem 5 guarantee that enough of these terms do not vanish for every choice of $U_{1}(\textbf{k})$, $U_{2}(\textbf{k})$ to force the ferromagnetic Slater determinant to be the unique minimizer of the Fock energy.

To prove the assumptions of Theorem 5 are sufficient, we proceed in two steps. First we show that for one special $\textbf{k}$-point, $\textbf{k}_{*}$, enough of the entries of $B_{\pm ,\textbf{k}_{*}}(\textbf{G})$ do not vanish to force $\theta _{i}(\textbf{k}_{*}) = \theta _{j}(\textbf{k}_{*}) = 0$ or $\theta _{i}(\textbf{k}_{*}) = \theta _{j}(\textbf{k}_{*}) = \pi$ for all $i,j \in \{ 1, \cdots , M\}$. This implies that the Fock energy maximizing 1-RDM at $\textbf{k}_{*}$ is a ferromagnetic Slater determinant. After showing this, we use the fact that the grid $\mathcal {K}$ has been chosen sufficiently finely so that Assumption 2 holds. Once this is the case, for any $\textbf{k}\in \mathcal {K}$, we can find a path connecting $\textbf{k}_{*}$ and $\textbf{k}$ and we will show that for all momenta along this path, the minimizing 1-RDM must be the same ferromagnetic Slater determinant as at $\textbf{k}_{*}$.

#### Local uniqueness of ground state

We focus on the point $\textbf{k}_{*}$, take $\textbf{q}= \varvec{0}$ and $i = j$. In this case, Eq. (7.14) reduces to

7.17 $$\begin{aligned} \frac{1}{2} | [B^{(1)}_{\textbf{k}_{*}}(\textbf{G}) + B^{(2)}_{\textbf{k}_{*}}(\textbf{G})]_{ii} |^{2} + \frac{1}{2} | [B^{(1)}_{\textbf{k}_{*}}(\textbf{G}) - B^{(2)}_{\textbf{k}_{*}}(\textbf{G})]_{ii} |^2 \cos {(2 \theta _{i}(\textbf{k}_{*}) )}. \end{aligned}$$

Now notice that

7.18 $$\begin{aligned} \operatorname {Im}{{{\,\textrm{tr}\,}}{(A_{\textbf{k}_{*}}(\textbf{G}))}}&= \frac{1}{2i} {{\,\textrm{tr}\,}}{(A_{\textbf{k}_{*}}(\textbf{G}) - \overline{A_{\textbf{k}_{*}}(\textbf{G})})} \nonumber \\&= \frac{1}{2i} {{\,\textrm{tr}\,}}{\Big (U_{1}(\textbf{k}_{*}) A_{\textbf{k}_{*}}(\textbf{G}) U_{1}(\textbf{k}_{*})^{\dagger } - U_{2}(\textbf{k}_{*}) \overline{A_{\textbf{k}_{*}}(\textbf{G})} U_{2}(\textbf{k}_{*})^{\dagger }\Big )} \nonumber \\&= \frac{1}{2i} {{\,\textrm{tr}\,}}{(B^{(1)}_{\textbf{k}_{*}}(\textbf{G}) - B^{(2)}_{\textbf{k}_{*}}(\textbf{G}))}. \end{aligned}$$

Since by assumption $\operatorname {Im}{{{\,\textrm{tr}\,}}{(A_{\textbf{k}_{*}}(\textbf{G}))}} \ne 0$, it must be there exists an *m* so that $[B^{(1)}_{\textbf{k}_{*}}(\textbf{G}) - B^{(2)}_{\textbf{k}_{*}}(\textbf{G})]_{mm} \ne 0$. Therefore, to be an optimizer $\cos {(2 \theta _{m}(\textbf{k}_{*}) )} = 1$ which implies $\theta _{m}(\textbf{k}_{*}) \in \{ 0, \pi \}$. Now we show that $\theta _{j}(\textbf{k}_{*}) = \theta _{m}(\textbf{k}_{*})$ for all *j*.

For this part of the proof, we fix the unitary $U_{1}(\textbf{k}_{*})$ and show that for this choice of $U_{1}(\textbf{k}_{*})$ all of the $\theta _{j}(\textbf{k}_{*})$ must agree. For this let $\{ \vert i\rangle : i \in \{ 1, \cdots , M\} \}$ denote the standard basis for ${\mathbb {C}}^{M}$ so that for any matrix $A_{ij} = \langle i | A |j\rangle$.

We will prove this result by induction. Let $m_{1} = m$ and suppose that we have already shown that the angles $\{ \theta _{m_{i}}(\textbf{k}_{*}): i \in \{ 1, \cdots , n\} \}$ are all equal to $\theta _{m_{1}}(\textbf{k}_{*}) \in \{ 0, \pi \}$. For fixed $U_{1}(\textbf{k}_{*})$, consider the orthogonal projector

7.19 $$\begin{aligned} \Pi = U_{1}(\textbf{k}_{*})^{\dagger } \left( \sum _{i=1}^{n} \vert m_{i}\rangle \langle m_{i}\vert \right) U_{1}(\textbf{k}_{*}). \end{aligned}$$

For any $\textbf{G}$ we have

7.20 $$\begin{aligned} (I&- \Pi ) A_{\textbf{k}_{*}}(\textbf{G}) \Pi \nonumber \\&= \left( I - U_{1}(\textbf{k}_{*})^{\dagger } \left( \sum _{i=1}^{n} \vert m_{i}\rangle \langle m_{i}\vert \right) U_{1}(\textbf{k}_{*})\right) A_{\textbf{k}_{*}}(\textbf{G}) \left( U_{1}(\textbf{k}_{*})^{\dagger } \left( \sum _{i=1}^{n} \vert m_{i}\rangle \langle m_{i}\vert \right) U_{1}(\textbf{k}_{*}) \right) \nonumber \\&= U_{1}(\textbf{k}_{*})^{\dagger } \left( I - \sum _{i=1}^{n} \vert m_{i}\rangle \langle m_{i}\vert \right) U_{1}(\textbf{k}_{*}) A_{\textbf{k}_{*}}(\textbf{G}) U_{1}(\textbf{k}_{*})^{\dagger } \left( \sum _{i=1}^{n} \vert m_{i}\rangle \langle m_{i}\vert \right) U_{1}(\textbf{k}_{*}). \end{aligned}$$

But since the spectral norm is unitarily invariant, the second assumption of Theorem 5 implies that there exists a $\textbf{G}'$ so that

7.21 $$\begin{aligned} \left\| \left( I - \sum _{i=1}^{n} \vert m_{i}\rangle \langle m_{i}\vert \right) U_{1}(\textbf{k}_{*}) A_{\textbf{k}_{*}}(\textbf{G}') U_{1}(\textbf{k}_{*})^{\dagger } \left( \sum _{i=1}^{n} \vert m_{i}\rangle \langle m_{i}\vert \right) \right\| > 0. \end{aligned}$$

Let *v*, *w* be the top right/left singular vectors of the above operator. Since $\sum _{i=1}^{n} \vert m_{i}\rangle \langle m_{i}\vert$ is an orthogonal projection and $\{ \vert i\rangle : i \in \{1, \cdots , M\}\}$ forms a complete basis we can write

7.22 $$\begin{aligned} v = \sum _{i=1}^{n} \alpha _{i} \vert m_{i}\rangle , \qquad w = \sum _{m \not \in \{ m_{i}: i \in \{ 1, \cdots , n \} \}} \beta _{m} \vert m\rangle \end{aligned}$$

for some constants $\alpha _{i}, \beta _{i} \in {\mathbb {C}}$. By definition of *v*, *w* we know that

7.23 $$\begin{aligned} \begin{aligned} 0&< \langle w | U_{1}(\textbf{k}_{*}) A_{\textbf{k}_{*}}(\textbf{G}') U_{1}(\textbf{k}_{*})^{\dagger }| v\rangle \\&= \sum _{i=1}^{n} \sum _{m \not \in \{ m_{i}: i \in \{ 1, \cdots , n \} \}} \alpha _{i} \overline{\beta _{m}} \langle m | U_{1}(\textbf{k}_{*}) A_{\textbf{k}_{*}}(\textbf{G}') U_{1}(\textbf{k}_{*})^{\dagger } | m_{i } \rangle . \end{aligned} \end{aligned}$$

Hence, there must exist an $m' \not \in \{ m_{i}: i \in \{ 1, \cdots , n \} \}$ and an $m_{i}$ so that

7.24 $$\begin{aligned} [U_{1}(\textbf{k}_{*}) A_{\textbf{k}_{*}}(\textbf{G}') U_{1}(\textbf{k}_{*})^{\dagger }]_{m',m_{i}} \ne 0. \end{aligned}$$

Define $$m_{n+1}:= m'$$, since

7.25 $$\begin{aligned} \Big (B^{(1)}_{\textbf{k}_{*}}(\textbf{G}') + B^{(2)}_{\textbf{k}_{*}}(\textbf{G}')\Big ) + \Big (B^{(1)}_{\textbf{k}_{*}}(\textbf{G}') - B^{(2)}_{\textbf{k}_{*}}(\textbf{G}')\Big ) = 2 U_{1}(\textbf{k}_{*}) A_{\textbf{k}_{*}}(\textbf{G}') U_{1}(\textbf{k}_{*})^{\dagger } \end{aligned}$$

Equation 7.24 implies that either $[B^{(1)}_{\textbf{k}_{*}}(\textbf{G}') + B^{(2)}_{\textbf{k}_{*}}(\textbf{G}')]_{m_{n+1},m_{i}} \ne 0$ or $[B^{(1)}_{\textbf{k}_{*}}(\textbf{G}') - B^{(2)}_{\textbf{k}_{*}}(\textbf{G}')]_{m_{n+1},m_{i}} \ne 0$. For simplicity of discussion suppose $[B^{(1)}_{\textbf{k}_{*}}(\textbf{G}') + B^{(2)}_{\textbf{k}_{*}}(\textbf{G}')]_{m_{n+1},m_{i}} \ne 0$, the other case follows similarly. Now recall the terms in the Fock energy:

7.26 $$\begin{aligned} \begin{aligned} | [B^{(1)}_{\textbf{k}_{*}}(\textbf{G}')&+ B^{(2)}_{\textbf{k}_{*}}(\textbf{G}')]_{m_{n+1},m_{i}} |^2 \cos {(\theta _{m_{n+1}}(\textbf{k}_{*}) - \theta _{m_{i}}(\textbf{k}_{*}))} \\&+ | [B^{(1)}_{\textbf{k}_{*}}(\textbf{G}') - B^{(2)}_{\textbf{k}_{*}}(\textbf{G}')]_{m_{n+1},m_{i}} |^2 \cos {(\theta _{m_{n+1}}(\textbf{k}_{*}) + \theta _{m_{i}}(\textbf{k}_{*}))}. \end{aligned} \end{aligned}$$

Since $\theta _{m_{i}}(\textbf{k}_{*}) = \theta _{m_{1}}(\textbf{k}_{*})$, $\theta _{m_1}(\textbf{k}_{*}) \in \{ 0, \pi \}$, and $[B^{(1)}_{{\textbf {k}}_{*}}({\textbf {G}}') + B^{(2)}_{{\textbf {k}}_{*}}({\textbf {G}}')]_{m_{n+1},m_{i}} \ne 0$, to be a minimizer it must be that $\theta _{m_{n+1}}(\textbf{k}_{*}) = \theta _{m_{1}}(\textbf{k}_{*})$. Hence by induction, $\theta _{i}(\textbf{k}_{*}) \in \{ 0, \pi \}$ and $\theta _{i}(\textbf{k}_{*}) = \theta _{j}(\textbf{k}_{*})$ for all *i*, *j*.

#### Global uniqueness of ground state

For simplicity, let us assume that $\theta _{j}(\textbf{k}_{*}) = 0$ for all *j*. The case $\theta _{j}(\textbf{k}_{*}) = \pi$ follows similarly. In this case, for any $\textbf{q}+ \textbf{G}$ we have that the terms corresponding to $\textbf{k}_{*}$ and $\textbf{q} + \textbf{G}$ in Eq. (7.13) are

7.27 $$\begin{aligned} \begin{aligned} \sum _{ij}  \frac{1}{2}&| [B^{(1)}_{\textbf{k}_{*}}(\textbf{q}+ \textbf{G}) + B^{(2)}_{\textbf{k}_{*}}(\textbf{q}+ \textbf{G})]_{ij} |^2 \cos {(\theta _{j}(\textbf{k}_{*} + \textbf{q}))} \\&+ \frac{1}{2} | [B^{(1)}_{\textbf{k}_{*}}(\textbf{q}+ \textbf{G}) - B^{(2)}_{\textbf{k}_{*}}(\textbf{q}+ \textbf{G})]_{ij} |^2 \cos {(\theta _j(\textbf{k}_{*} + \textbf{q}))}. \end{aligned} \end{aligned}$$

It’s clear that this term is maximized (and hence the Fock energy is minimized) if $\theta (\textbf{k}_{*} + \textbf{q}) = 0$. To prove that $\theta _{j}(\textbf{k}_{*} + \textbf{q}) = 0$ is the unique maximizer, it is enough to show that for each $j \in \{1, \cdots , M\}$

7.28 $$\begin{aligned} \text {one of} \quad {\left\{ \begin{array}{ll} \max _{m} |[B^{(1)}_{\textbf{k}_{*}}(\textbf{q}+ \textbf{G}) + B^{(2)}_{\textbf{k}_{*}}(\textbf{q}+ \textbf{G})]_{mj}|> 0 &  \\ \max _{m} |[B^{(1)}_{\textbf{k}_{*}}(\textbf{q}+ \textbf{G}) - B^{(2)}_{\textbf{k}_{*}}(\textbf{q}+ \textbf{G})]_{mj}| > 0 &  \\ \end{array}\right. } \quad \text {holds}. \end{aligned}$$

That is, for each row, we can find a non-zero entry in one of $B^{(1)}_{\textbf{k}_{*}}(\textbf{q}+ \textbf{G}) \pm B^{(2)}_{\textbf{k}_{*}}(\textbf{q}+ \textbf{G})$. Notice that

7.29 $$\begin{aligned} \begin{aligned}&2 U_{1}(\textbf{k}_{*}) A_{\textbf{k}_{*}}(\textbf{q}+ \textbf{G}) U_{1}(\textbf{k}_{*} + \textbf{q})^{\dagger } \\&\hspace{2em} = \Big ( B^{(1)}_{\textbf{k}_{*}}(\textbf{q}+ \textbf{G}) + B^{(2)}_{\textbf{k}_{*}}(\textbf{q}+ \textbf{G}) \Big ) + \Big ( B^{(1)}_{\textbf{k}_{*}}(\textbf{q}+ \textbf{G}) - B^{(2)}_{\textbf{k}_{*}}(\textbf{q}+ \textbf{G}) \Big ) \\ \end{aligned} \end{aligned}$$

By Lemma 4.1, since $A_{\textbf{k}_{*}}(\textbf{q}+ \textbf{G})$ is a principal submatrix of $\Lambda _{\textbf{k}}(\textbf{q} + \textbf{G})$, for all $| \textbf{q} + \textbf{G} | < \frac{1}{L}$, $A_{\textbf{k}_{*}}(\textbf{q} + \textbf{G})$ is of full rank. Since a full rank matrix must have at least one non-zero entry in every row, it follows that Eq. (7.28) holds for all $\textbf{q} + \textbf{G}$ sufficiently small. Hence $\theta _{j}(\textbf{k}_{*} + \textbf{q}) = 0$ for all *j* for all $|\textbf{q}| < \frac{1}{L}$.

Using the above argument, we started with the assumption that $\theta _{j}(\textbf{k}_{*}) = 0$ for some $\textbf{k}_{*}$ and have shown that for a state be a minimizer, it must be that $\theta _{j}(\textbf{k}) = 0$ for all $\textbf{k}$ within a ball of $\textbf{k}_{*}$. Importantly, the radius of this ball is independent of the original point $\textbf{k}_{*}$. Since the set $\Omega ^{*}$ is compact, by using Assumption 2, we can repeat this argument to conclude that $\theta _{j}(\textbf{k}) = 0$ for all $\textbf{k} \in {\mathcal {K}}$ completing the proof.

## Applications of Theorem 5 to TBG and eTTG

We begin by translating the conditions of Theorem 5 from momentum space to real space in Sect. 8.1. We then prove Result 3 by verifying these real space conditions hold for TBG-2 (Sect. 8.2), TBG-4 (Sect. 8.3), and eTTG-4 (Sect. 8.4). This will rely on explicit knowledge of the *k*-dependence of the flat band Bloch functions for these various configurations.

### Conditions of Theorem 5 in Real Space

While thus far we have performed our calculations in momentum space, it is more convenient to verify the assumptions of Theorem 5 in real space. We recall that $A_{\textbf{k}}(\textbf{q} + \textbf{G})$ are the Fourier series coefficients of the pair product, $\rho _{\textbf{k}, \textbf{k}+\textbf{q}}(\textbf{r})$ (Eq. (2.16)).

8.1 $$\begin{aligned} \begin{aligned} \,[A_{\textbf{k}}(\textbf{q}+ \textbf{G})]_{mn}&= \int _{\Omega } e^{-i \textbf{G}\cdot \textbf{r}} \rho _{\textbf{k}, \textbf{k}+\textbf{q}}(\textbf{r}) \,\textrm{d}\textbf{r}\\&= \int _{\Omega } e^{-i \textbf{G}\cdot \textbf{r}} \sum _{\sigma ,j} \overline{u_{m\textbf{k}}(\textbf{r}; \sigma , j)} u_{n(\textbf{k}+ \textbf{q})}(\textbf{r}; \sigma , j) \,\textrm{d}\textbf{r}. \end{aligned} \end{aligned}$$

Since the Fourier transform is isometric up to scaling, we can equivalently state conditions for Theorem 5 in real space.

#### [Style2 Style2]Proposition 8.1

(Theorem 5 in Real Space) . Suppose that the single particle Hamiltonian *H* satisfies Assumptions 1, 3. Suppose further that the Monkhorst-Pack grid $\mathcal {K}$ has been chosen to satisfy Assumption 2. If there exists a $\textbf{k}\in \mathcal {K}$ so that

1. For some $$\textbf{r}$$, $${{\,\textrm{tr}\,}}{(\rho _{\textbf{k},\textbf{k}}(\textbf{r}))} \ne \overline{{{\,\textrm{tr}\,}}{(\rho _{\textbf{k},\textbf{k}}(-\textbf{r}))}}$$
2. For all non-trivial projections $\Pi$, there exists a $\textbf{r}'$ so that
  8.2 $$\begin{aligned} \Vert (I - \Pi ) \rho _{\textbf{k},\textbf{k}}(\textbf{r}') \Pi \Vert > 0 \end{aligned}$$

then the two ferromagnetic Slater determinants are the unique Hartree-Fock ground states of Eq. (2.25).

We also have the following simple corollaries:

#### Corollary 8

(Two Bands Case in Real Space). Suppose that the single particle Hamiltonian *H* satisfies Assumptions 1, 3 and has two flat bands. Suppose further that the Monkhorst-Pack grid $\mathcal {K}$ has been chosen to satisfy Assumption 2. If there exists a $\textbf{k}\in \mathcal {K}$ and $\textbf{r}\in \Omega$ so that $\rho _{\textbf{k},\textbf{k}}(\textbf{r}) \ne \overline{\rho _{\textbf{k},\textbf{k}}(-\textbf{r})}$ then the two ferromagnetic Slater determinants are the unique Hartree-Fock ground states of Eq. (2.25).

#### Corollary 9

(Four Band Case in Real Space). Suppose that the single particle Hamiltonian *H* satisfies Assumptions 1, 3 and has four flat bands. Suppose further that the Monkhorst-Pack grid $\mathcal {K}$ has been chosen to satisfy Assumption 2. If there exists a $\textbf{k}\in \mathcal {K}$ so that

1. For some $$\textbf{r}$$, $${{\,\textrm{tr}\,}}{(\rho _{\textbf{k},\textbf{k}}(\textbf{r}))} \ne \overline{{{\,\textrm{tr}\,}}{(\rho _{\textbf{k},\textbf{k}}(-\textbf{r}))}}$$,
2. For some $$\textbf{r}', \textbf{r}''$$
  8.3 $$\begin{aligned} \det { \begin{bmatrix} \Vert u_{1\textbf{k}}(\textbf{r}')\Vert ^2 - \Vert u_{2\textbf{k}}(\textbf{r}')\Vert ^2 &  \langle u_{1\textbf{k}}(\textbf{r}'), u_{2\textbf{k}}(\textbf{r}')\rangle \\ \Vert u_{1\textbf{k}}(\textbf{r}'')\Vert ^2 - \Vert u_{2\textbf{k}}(\textbf{r}'')\Vert ^2 &  \langle u_{1\textbf{k}}(\textbf{r}''), u_{2\textbf{k}}(\textbf{r}'')\rangle \end{bmatrix}} \ne 0 \end{aligned}$$

then the two ferromagnetic Slater determinants are the unique Hartree-Fock ground states of Eq. (2.25).

For the proofs of Propositions 8.1, 8, 9 we refer the reader to Sect. D.

### Application of main theorem to TBG-2

For our next two propositions, we will use the Jacobi $\theta$ function

8.4 $$\begin{aligned} \begin{gathered} \theta _{1} ( \zeta | \omega ):= - \sum _{ n \in \mathbb {Z} } \exp ( \pi i (n+\tfrac{1}{2}) ^2 \omega + 2 \pi i ( n + \tfrac{1}{2} ) (\zeta + \tfrac{1}{2} ) ), \end{gathered} \end{aligned}$$

which satisfies

$$\begin{aligned} \theta _{1} ( \zeta + m | \omega ) = (-1)^m \theta _{1} ( \zeta | \omega ), \ \ \theta _{1} ( \zeta + n \omega | \omega ) = (-1)^n e^{ - \pi i n^2 \omega - 2 \pi i \zeta n } \theta _{1} ( \zeta |\omega ). \end{aligned}$$

Furthermore, $\theta _1(\bullet \vert \omega )$ has simple zeros at ${\mathbb {Z}}+ \omega {\mathbb {Z}}$ (and no other zeros) – see [45]. In the sequel we shall just write $\theta (\zeta ):=\theta _1(\zeta \vert \omega )$ to simplify the notation.

We also use the $\wp$ function $\wp (z):=\wp ( z; \tfrac{4}{3} \pi i \omega , \tfrac{4}{3} \pi i \omega ^2 )$. Here $\wp ( z; \omega _1, \omega _2 )$ is the Weierstrass $\wp$-function – see [45, SI.6]. It is periodic with respect to ${\mathbb {Z}}\omega _1 + {\mathbb {Z}}\omega _2$ and its derivative has a pole of order 3 at $z = 0$. It has the property that

8.5 $$\begin{aligned} \wp (\omega z) = \omega \wp (z). \end{aligned}$$

We now turn to verifying the conditions of having a unique many-body ground state.

#### [Style2 Style2]Proposition 8.2

(Simple magic angle) . Let $\alpha \in {\mathbb {C}}$ be a simple magic angle of TBG-2, then for all $\textbf{k}\notin \Gamma ^*$ there is $\textbf{G}\in \Gamma ^*$ such that

$$\begin{aligned} \operatorname {Im}{{\,\textrm{tr}\,}}(A_{\textbf{k}}(\textbf{G}))\ne 0\end{aligned}$$

while for $\textbf{k}\in \Gamma ^*$ one has

8.6 $$\begin{aligned} \operatorname {Im}{{\,\textrm{tr}\,}}(A_{\textbf{k}}(\textbf{G}))=0 \text { for all }\textbf{G}\in \Gamma ^*. \end{aligned}$$

#### Proof

Since the magic angle is simple, $A_{\textbf{k}}(\textbf{G})$ is completely determined by the periodic Bloch function $u_{1\textbf{k}}$. For notational simplicity, in this proof we will drop the band index and simply write $u_{\textbf{k}}$. From Corollary 8 we deduce that the existence of some $\textbf{G}\in \Gamma ^*$ such that

$$\begin{aligned} \operatorname {Im}{{\,\textrm{tr}\,}}(A_{\textbf{k}}(\textbf{G}))\ne 0\end{aligned}$$

is equivalent to the existence of some $\textbf{r}$ such that $\Vert \textbf{u}_{\textbf{k}}(\textbf{r})\Vert \ne \Vert \textbf{u}_{\textbf{k}}(-\textbf{r})\Vert$, where we use here the Euclidean norm of the 2-vector $\textbf{u}_{\textbf{k}}$ that is given by

$$\begin{aligned} \textbf{u}_{\textbf{k}} \in \ker _{L^2_2}(D(\alpha )+(k_1+ik_2) \operatorname {id}_{\mathbb {C}^2}) {\setminus }\{0\}.\end{aligned}$$

**Fig. 4:**
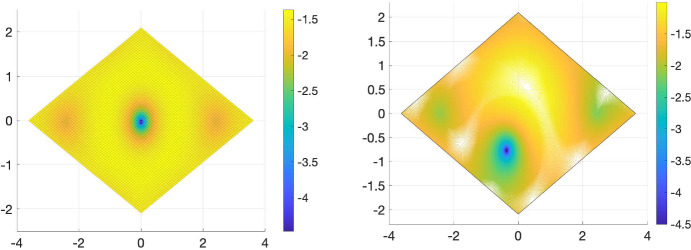
Log of modulus of flat-band wavefunction with $U_0$ as in (3.14) and $\textbf{k}=0$, $\alpha \approx 0.58656$ exhibiting reflection symmetry with zero in the center (left) and $\textbf{k}=(2,0.7)$ with zero away from center (right) showing $\Vert u_{\textbf{k}}(\textbf{r})\Vert \ne \Vert u_{\textbf{k}}(-\textbf{r})\Vert .$

At simple magic angles there is a unique solution $\textbf{u}_{{\textbf{0}}}$ which obey reflection symmetry, see [23, Theo.1] together with [23, (2.12)] (also Fig. 4 for an illustration).

Indeed, using symmetry (3.11), we find

$$\begin{aligned} \Vert \textbf{u}_{{\textbf{0}}}(\textbf{r})\Vert =\Vert {\mathcal {L}} \textbf{u}_{\textbf{0}}(\textbf{r}) \Vert = \Vert \textbf{u}_{{\textbf{0}}}(-\textbf{r})\Vert \end{aligned}$$

showing (8.6) by the equivalence from Corollary 9

$$\begin{aligned} \operatorname {Im}{{\,\textrm{tr}\,}}(A_{\textbf{k}}(\textbf{G})) \ne 0 \text { for some }\textbf{G}\text { if and only if }{{\,\textrm{tr}\,}}(\rho _{\textbf{k},\textbf{k}}(\textbf{r})) \ne {{\,\textrm{tr}\,}}(\rho _{\textbf{k},\textbf{k}}(-\textbf{r}))\text { for some }\textbf{r}.\end{aligned}$$

In [24, Theo. 3], it was shown that the function ${\textbf{u}}_{{\textbf{0}}}$ has a simple zero at ${\textbf{r}}={\textbf{0}}$ and no other zeros in its fundamental domain. Using this knowledge and the special structure of the operator $D(\alpha )$ which is a first order operator with derivatives only depending on ${\bar{z}}$, it is possible to find a relationship between $\textbf{u}_{{\textbf{0}}} \in \ker _{L^2_2}(D(\alpha ))$ and $\textbf{u}_{{\textbf{k}}} \in \ker (D(\alpha )+(k_1+ik_2)\operatorname {id}_{{\mathbb {C}}^2})$. Indeed, from [24, Lemma 3.1],

8.7 $$\begin{aligned} \textbf{u}_{\textbf{k}}(\textbf{r}) = F_{\textbf{k}}(\textbf{r}) \textbf{u}_{{\textbf{0}}}(\textbf{r}) \in \ker (D(\alpha )+(k_1+ik_2)\operatorname {id}_{{\mathbb {C}}^2}), \end{aligned}$$

where

$$\begin{aligned} F_{\textbf{k}}(\textbf{r}):=e^{\frac{(k_1+ik_2)}{2}(-i(1+\omega )x_1+(\omega -1)x_2)} \frac{ \theta ( \frac{3(x_1+ix_2)}{4\pi i \omega } + \frac{k_1+ik_2}{\sqrt{3}\omega } ) }{ \theta \Big ( \frac{3(x_1+ix_2)}{4\pi i \omega }\Big )}.\end{aligned}$$

Since, as mentioned above $\textbf{u}_{{\textbf{0}}}$ has a unique zero at zero that is canceled by the theta function in the denominator of $F_{\textbf{k}}$, it follows that $\textbf{u}_{\textbf{k}}(\textbf{r})$ has a unique zero at $\textbf{r}= \frac{4\pi }{3\sqrt{3}}(k_2,-k_1)^\top$ per unit cell, which is the zero of the theta function in the nominator of $F_{\textbf{k}}$. Thus, due to the unique zero that is not located at the origin, $\Vert \textbf{u}_{\textbf{k}}\Vert$ cannot be an even function for $\textbf{k}\notin \Gamma ^*.$ $\square$

Thus, by (8.7), since $\textbf{k}\mapsto F_{\textbf{k}}$ is holomorphic,

8.8 $$\begin{aligned} \textbf{k}\mapsto \Pi (\textbf{k}):=\frac{[\textbf{u}_{\textbf{k}},0]^{\top } \otimes [\textbf{u}_{\textbf{k}},0]^{\top }}{\Vert \textbf{u}_{\textbf{k}} \Vert _2^2 } + \frac{[0,\mathcal {Q}\textbf{u}_{\textbf{k}}]^{\top } \otimes [0,\mathcal {Q}\textbf{u}_{\textbf{k}}]^{\top }}{\Vert \textbf{u}_{\textbf{k}} \Vert _2^2 } \end{aligned}$$

is a real-analytic projection in ($k_1,k_2$) onto the flat band eigenfuctions. This is a direct way of seeing that Corollary 2.1 holds for the projection of the flat band Bloch functions. Corollary 2.1 can also be applied directly, since at magic angles in TBG-2, the flat bands is gapped from the remaining bands by [24, Theo. 2].

Therefore, by Corollary 8, we have the following result

#### [Style2 Style2]Theorem 10

The ferromagnetic Slater determinant states are the unique ground states of the corresponding flat-band interacting model of TBG-2.

### Application of main theorem to TBG-4

A similar result also holds for two-fold degenerate magic angles in chiral limit TBG which exhibits four flat bands at zero energy at magic angles.

#### [Style2 Style2]Proposition 8.3

(Two-fold degenerate magic angle) . Let $\alpha \in {\mathbb {C}}$ be a two-fold degenerate magic angle of TBG-4, then for $\textbf{k}= \pm \textbf{q}_1$ there are $\textbf{G}\in \Gamma ^*$ such that

8.9 $$\begin{aligned} \operatorname {Im}{{\,\textrm{tr}\,}}(A_{\textbf{k}}(\textbf{G}))\ne 0, \end{aligned}$$

while for $\textbf{k}\in \Gamma ^*$ one has

8.10 $$\begin{aligned} \operatorname {Im}{{\,\textrm{tr}\,}}(A_{\textbf{k}}(\textbf{G}))=0 \text { for all }\textbf{G}\in \Gamma ^*. \end{aligned}$$

#### Proof

For a two-fold degenerate magic angle, there is by [23, Theo.1], a unique element $\textbf{w}_{{\textbf{0}}} \in \ker _{L^2_{2,1}}(D(\alpha ))$ and $\textbf{v}_{\textbf{0}}(\textbf{r}) = \wp (x_1+ix_2)\textbf{w}_{{\textbf{0}}}(\textbf{r}) \in \ker _{L^2_{2,0}}(D(\alpha )).$

Since they belong to $L^2$-orthogonal subspaces, as they obey different rotational constraints by (8.5), we have ${{\,\textrm{tr}\,}}(A_{{\textbf{0}}}(\textbf{r})) = \Vert \textbf{v}_{{\textbf{0}}}(\textbf{r}) \Vert ^2 + \Vert \textbf{w}_{{\textbf{0}}}(\textbf{r}) \Vert ^2.$ Thus, by the symmetry (3.11), we find

8.11 $$\begin{aligned} \begin{aligned} {{\,\textrm{tr}\,}}(A_{{\textbf{0}}}(\textbf{r}))&= \Vert \textbf{w}_{{\textbf{0}}}(\textbf{r}) \Vert ^2 + \Vert \textbf{v}_{{\textbf{0}}}(\textbf{r}) \Vert ^2 \\&= \Vert {\mathcal {L}} \textbf{w}_{{\textbf{0}}}(\textbf{r}) \Vert ^2 + \Vert {\mathcal {L}} \textbf{v}_{{\textbf{0}}}(\textbf{r}) \Vert ^2\\&= \Vert \textbf{w}_{{\textbf{0}}}(-\textbf{r}) \Vert ^2 + \Vert \textbf{v}_{{\textbf{0}}}(-\textbf{r}) \Vert ^2 = {{\,\textrm{tr}\,}}(A_{{\textbf{0}}}(-\textbf{r})). \end{aligned} \end{aligned}$$

We recall from Corollary 9 the equivalence

$$\begin{aligned} \operatorname {Im}{{\,\textrm{tr}\,}}(A_{\textbf{k}}(\textbf{G})) \ne 0 \text { for some }\textbf{G}\text { if and only if }{{\,\textrm{tr}\,}}(\rho _{\textbf{k},\textbf{k}}(\textbf{r})) \ne {{\,\textrm{tr}\,}}(\rho _{\textbf{k},\textbf{k}}(-\textbf{r}))\text { for some }\textbf{r}\end{aligned}$$

showing (8.10), as all $\textbf{k}\in \Gamma ^*$ are equivalent to $\textbf{k}={\varvec{0}}.$

**Fig. 5:**
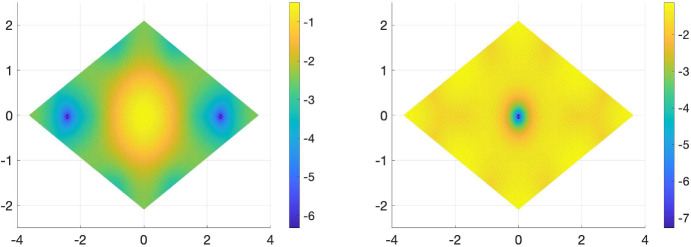
Log of modulus of flat-band wavefunctions two flat bands for $U_{7/8}$ as in (3.14) with $\textbf{k}=0$, $\alpha \approx 0.853799$ with $\textbf{v}_{{\textbf{0}}}$ (left) (with simple zeros at $\pm \textbf{r}_{S}$ and $\textbf{w}_{{\textbf{0}}}$(right) with double zero at $\textbf{r}=0$

We recall from [23, Theo. 3] and [23, Lemm. 7.1] that $\textbf{w}_{{\textbf{0}}}$ has a zero of order 2 at ${\textbf{r}}={\textbf{0}}$ and $\textbf{v}_{\textbf{0}}$ has simple zeros at $\pm \textbf{r}_S$ with $\textbf{r}_S= (\frac{4\pi }{3\sqrt{3}},0)^{\top },$ see Fig. 5.

We then define functions $\textbf{v}_{\textbf{k}}, \textbf{w}_{\textbf{k}} \in \ker (D(\alpha )+(k_1+ik_2)\operatorname {id}_{{\mathbb {C}}^2})$ by

$$\begin{aligned} \textbf{v}_{\textbf{k}}(\textbf{r}) = \alpha _{\textbf{k}} F_{\textbf{k}}(\textbf{r}- \textbf{r}_S) \textbf{v}_{{\textbf{0}}}(\textbf{r}) \text { and }\textbf{w}_{\textbf{k}}(\textbf{r}) = \beta _{\textbf{k}} F_{\textbf{k}}(\textbf{r})\textbf{w}_{{\textbf{0}}}(\textbf{r})\end{aligned}$$

with normalizing constants $\alpha _{\textbf{k}},\beta _{\textbf{k}}>0$ that we allow to change in this proof to simplify the notation and

8.12 $$\begin{aligned} F_{\textbf{k}}(\textbf{r}):=e^{\frac{(k_1+ik_2)}{2}(-i(1+\omega )x_1+(\omega -1)x_2)} \frac{ \theta ( \frac{3(x_1+ix_2)}{4\pi i \omega } + \frac{k_1+ik_2}{\sqrt{3}\omega } ) }{ \theta \Big ( \frac{3(x_1+ix_2)}{4\pi i \omega }\Big )}. \end{aligned}$$

From (8.7), we see that $\textbf{v}_{\textbf{k}_*},\textbf{w}_{\textbf{k}_*} \in \ker (D(\alpha )+i \operatorname {id}_{{\mathbb {C}}^2})$ for $\textbf{k}_*:=(0,1)^{\top }.$ We observe directly from the function $F_{{\textbf{k}}}$ that $\textbf{w}_{\textbf{k}_*}$ vanishes to first order at ${\textbf{r}} =0$ and ${\textbf{r}}=\textbf{r}_S$, but not at any other point. The function $\textbf{v}_{\textbf{k}_*}$ vanishes to second order at $\textbf{r}=-\textbf{r}_S$, i.e. $\textbf{v}_{\textbf{k}_*}(-\textbf{r}_S)=0$ and $\nabla \textbf{v}_{\textbf{k}_*}(-\textbf{r}_S)=0$.

We conclude that $\textbf{v}_{\textbf{k}_*},\textbf{w}_{\textbf{k}_*}$ are $L^2$-orthogonal using (8.5), since we can write for $\gamma \ne 0$

8.13 $$\begin{aligned} \begin{aligned}\textbf{w}_{\textbf{k}_*}(\textbf{r}')=\gamma \wp (x_1'+ix_2'+\vert r_S\vert ) \textbf{v}_{\textbf{k}_*}(\textbf{r}'),\text { where }\\ \gamma ^:=\frac{\textbf{w}_{\textbf{k}_*}(-\textbf{r}_S)}{\Vert \wp (\bullet +\vert \textbf{r}_S\vert ) \textbf{v}_{\textbf{k}_*}(\bullet ) \Vert \lim _{x \rightarrow -\textbf{r}_S}(\wp (x+\vert \textbf{r}_S\vert ) \textbf{v}_{\textbf{k}_*}(x))} \end{aligned} \end{aligned}$$

and thus $\textbf{w}_{\textbf{k}_*}$ and $\textbf{v}_{\textbf{k}_*}$ have different rotational symmetries, i.e. $\textbf{w}_{\textbf{k}_*}(\omega (\textbf{r}-\textbf{r}_S))= \omega \textbf{w}_{\textbf{k}_*}(\textbf{r}-\textbf{r}_S)$ and $\textbf{v}_{\textbf{k}_*}(\omega (\textbf{r}-\textbf{r}_S))= \omega ^2 \textbf{v}_{\textbf{k}_*}(\textbf{r}-\textbf{r}_S).$ We thus have

8.14 $$\begin{aligned} \begin{aligned} {{\,\textrm{tr}\,}}(\rho _{\textbf{k}_*,\textbf{k}_*}(\textbf{r}))&= \Vert \textbf{w}_{\textbf{k}_*}(\textbf{r})\Vert ^2 +\Vert \textbf{v}_{\textbf{k}_*}(\textbf{r})\Vert ^2 \\&= (\alpha _{\textbf{k}_*} \vert F_{\textbf{k}_*}(\textbf{r}- \textbf{r}_S) \wp (x_1+ix_2)\vert ^2+\beta _{\textbf{k}_*} \vert F_{\textbf{k}_*}(\textbf{r}) \vert ^2)\Vert \textbf{w}_{{\textbf{0}}}(\textbf{r})\Vert ^2 \end{aligned} \end{aligned}$$

for some new $\alpha _{\textbf{k}_*},\beta _{\textbf{k}_*}>0.$ In addition, we record that

$$\begin{aligned}F_{\textbf{k}_*}(\textbf{r}-\textbf{r}_S) = e^{-\frac{2\pi }{3\sqrt{3}}(1+\omega )} e^{\frac{i}{2}(-i(1+\omega )x_1+(\omega -1)x_2)} \frac{ \theta ( \frac{3(x_1+ix_2)}{4\pi i \omega }+\frac{2i}{\sqrt{3}\omega } ) }{ \theta \Big ( \frac{3(x_1+ix_2)}{4\pi i \omega }+\frac{i}{\sqrt{3}\omega }\Big )}\end{aligned}$$

and

$$\begin{aligned}F_{\textbf{k}_*}(\textbf{r}) = e^{\frac{i}{2}(-i(1+\omega )x_1+(\omega -1)x_2)} \frac{ \theta ( \frac{3(x_1+ix_2)}{4\pi i \omega }+\frac{i}{\sqrt{3}\omega } ) }{ \theta \Big ( \frac{3(x_1+ix_2)}{4\pi i \omega }\Big )}.\end{aligned}$$

In particular, we have for the modulus of the prefactor of (8.12) as above

$$\begin{aligned}\vert e^{\frac{i}{2}(-i(1+\omega )x_1+(\omega -1)x_2)} \vert ^2 = e^{\frac{x_1-\sqrt{3}x_2}{2}}.\end{aligned}$$

Inserting the above expressions into (8.14), we find

$$\begin{aligned}\begin{aligned} {{\,\textrm{tr}\,}}(\rho _{\textbf{k}_*,\textbf{k}_*}(\textbf{r}))&=e^{\frac{x_1-\sqrt{3}x_2}{2}} \Bigg (\alpha _{\textbf{k}_*} \frac{ \vert \wp (x_1+ix_2) \theta ( \frac{3(x_1+ix_2)}{4\pi i \omega }+\frac{2i}{\sqrt{3}\omega } )\vert ^2 }{ \vert \theta \Big ( \frac{3(x_1+ix_2)}{4\pi i \omega }+\frac{i}{\sqrt{3}\omega }\Big )\vert ^2} + \beta _{\textbf{k}_*} \frac{ \vert \theta ( \frac{3(x_1+ix_2)}{4\pi i \omega }+\frac{i}{\sqrt{3}\omega } )\vert ^2 }{ \vert \theta \Big ( \frac{3(x_1+ix_2)}{4\pi i \omega }\Big )\vert ^2} \Bigg )\Vert \textbf{w}_{{\textbf{0}}}(\textbf{r})\Vert ^2. \end{aligned} \end{aligned}$$

We also notice that $2i/(\sqrt{3}\omega ) = -i/(\sqrt{3}\omega ) +(1-\omega )$ which allows us together with the following identity [46, p.105] for the Weierstrass function

$$\begin{aligned}\wp (x_1+ix_2) = - e^{-\frac{2\pi }{\sqrt{3}\omega }}\Bigg ( \frac{\theta '(0)}{\theta (\frac{i}{\sqrt{3}\omega })} \Bigg )^2 \frac{ \theta ( \tfrac{3(x_1+ix_2)}{4\pi i \omega }-\tfrac{i}{\sqrt{3}\omega } ) \theta ( \tfrac{3(x_1+ix_2)}{4\pi i \omega }+\tfrac{i}{\sqrt{3}\omega } )}{ \theta ( \tfrac{3(x_1+ix_2)}{4\pi i \omega } )^2} \end{aligned}$$

to reduce the above expression to

$$\begin{aligned}\begin{aligned} {{\,\textrm{tr}\,}}(\rho _{\textbf{k}_*,\textbf{k}_*}(\textbf{r}))&=e^{-(x_1-\sqrt{3}x_2)}\Bigg (\alpha _{\textbf{k}_*} \frac{\vert \theta ( \tfrac{3(x_1+ix_2)}{4\pi i \omega }-\tfrac{i}{\sqrt{3}\omega } )\vert ^4}{\vert \theta \Big ( \frac{3(x_1+ix_2)}{4\pi i \omega }\Big )\vert ^2}+ \beta _{\textbf{k}_*} \vert \theta ( \tfrac{3(x_1+ix_2)}{4\pi i \omega }+\tfrac{i}{\sqrt{3}\omega } )\vert ^2 \Bigg )\frac{\Vert \textbf{w}_{{\textbf{0}}}(\textbf{r})\Vert ^2}{\vert \theta \Big ( \tfrac{3(x_1+ix_2)}{4\pi i \omega }\Big )\vert ^2}. \end{aligned} \end{aligned}$$

Recalling that $\Vert \textbf{w}_{0} \Vert$ and $\vert \theta \vert$ are even, since $\Vert \textbf{w}_{0}(\textbf{r}) \Vert = \Vert {\mathcal {L}}\textbf{w}_{0}(\textbf{r}) \Vert = \Vert \textbf{w}_{0}(-\textbf{r}) \Vert$, then in order to study whether ${{\,\textrm{tr}\,}}(\rho _{\textbf{k}_*,\textbf{k}_*}(\textbf{r}))$ is even, we have to analyze whether for $\gamma :=\beta _{\textbf{k}_*}/\alpha _{\textbf{k}_*}>0,$

$$\begin{aligned}\Phi (\textbf{r}) = e^{-(x_1-\sqrt{3}x_2)}\Bigg ( \vert \theta ( \tfrac{3(x_1+ix_2)}{4\pi i \omega }-\tfrac{i}{\sqrt{3}\omega } )\vert ^4+ \gamma \vert \theta ( \tfrac{3(x_1+ix_2)}{4\pi i \omega }+\tfrac{i}{\sqrt{3}\omega } ) \theta \Big ( \tfrac{3(x_1+ix_2)}{4\pi i \omega }\Big )\vert ^2 \Bigg ) \end{aligned}$$

is even. We will show that this is false. Evaluating $\Phi$ on the lattice $\Gamma$ we have by [45, Lemma 4.1]

$$\begin{aligned} \textbf{r}= (x_1,x_2)^{\top } \in \Gamma \Leftrightarrow \theta \Big (\tfrac{3(x_1+ix_2)}{4\pi i \omega }\Big )=0,\end{aligned}$$

we find, assuming $\Phi$ to be even, that

$$\begin{aligned} e^{-(x_1-\sqrt{3}x_2)} \vert \theta ( \tfrac{3(x_1+ix_2)}{4\pi i \omega }-\tfrac{i}{\sqrt{3}\omega } )\vert ^4 = \Phi (\textbf{r}) =\Phi (-\textbf{r}) = e^{(x_1-\sqrt{3}x_2)} \vert \theta ( \tfrac{3(x_1+ix_2)}{4\pi i \omega }+\tfrac{i}{\sqrt{3}\omega } )\vert ^4. \end{aligned}$$

Choosing, explicitly lattice points $x_1 = 2\pi /\sqrt{3}$ and $x_2 = 2\pi /3$, which is just $-{\textbf{v}}_1$, see (2.1), we find

$$\begin{aligned} \vert \theta ( -1-\tfrac{i}{\sqrt{3}\omega } )\vert = \vert \theta ( -1+\tfrac{i}{\sqrt{3}\omega } )\vert \end{aligned}$$

which is false.

We have thus shown that

$$\begin{aligned}{{\,\textrm{tr}\,}}(\rho _{\textbf{k}_*,\textbf{k}_*}(\textbf{r})) \ne {{\,\textrm{tr}\,}}(\rho _{\textbf{k}_*,\textbf{k}_*}(-\textbf{r})). \end{aligned}$$

Either repeating the previous construction for $-\textbf{k}_*$ or observing that using (3.11)

$$\begin{aligned} {{\,\textrm{tr}\,}}(\rho _{-\textbf{k}_*,-\textbf{k}_*}(\textbf{r})) = \Vert {\mathcal {L}} \textbf{w}_{\textbf{k}_*}(r)\Vert ^2 +\Vert {\mathcal {L}} \textbf{v}_{\textbf{k}_*}(\textbf{r})\Vert ^2 = \Vert \textbf{w}_{\textbf{k}_*}(r)\Vert ^2 +\Vert \textbf{v}_{\textbf{k}_*}(\textbf{r})\Vert ^2 = {{\,\textrm{tr}\,}}(\rho _{\textbf{k}_*,\textbf{k}_*}(\textbf{r})), \end{aligned}$$

we conclude, by Corollary 9, that (8.9) holds. $\square$

Using the notation of the previous proof, we can now verify (8.3)

#### [Style2 Style2]Proposition 8.4

Let $\alpha$ be a two-fold degenerate magic angle of TBG, $\textbf{k}_*:=(0,1)^{\top }$, let $\textbf{r}\in \{{\textbf{0}},\pm \textbf{r}_S\}$, and $\textbf{r}' \notin \{{\textbf{0}},\pm \textbf{r}_S\} +\Gamma$. Then the expression

8.15 $$\begin{aligned} \begin{aligned} D(\textbf{r}, \textbf{r}')&:= \det { \begin{bmatrix} \Vert \textbf{w}_{\textbf{k}_*}(\textbf{r})\Vert ^2 - \Vert \textbf{v}_{\textbf{k}_*}(\textbf{r})\Vert ^2 &  \langle \textbf{w}_{\textbf{k}_*}(\textbf{r}), \textbf{v}_{\textbf{k}_*}(\textbf{r})\rangle \\ \Vert \textbf{w}_{\textbf{k}_*}(\textbf{r}')\Vert ^2 - \Vert \textbf{v}_{\textbf{k}_*}(\textbf{r}')\Vert ^2 &  \langle \textbf{w}_{\textbf{k}_*}(\textbf{r}'), \textbf{v}_{\textbf{k}_*}(\textbf{r}')\rangle \end{bmatrix}} \\&= (\Vert \textbf{w}_{\textbf{k}_*}(\textbf{r})\Vert ^2 - \Vert \textbf{v}_{\textbf{k}_*}(\textbf{r})\Vert ^2) \langle \textbf{w}_{\textbf{k}_*}(\textbf{r}'), \textbf{v}_{\textbf{k}_*}(\textbf{r}')\rangle \end{aligned} \end{aligned}$$

is non-zero.

#### Proof

From the proof of Proposition 8.3, we know that $\textbf{v}_{\textbf{k}_*}$ vanishes to second order precisely at $-\textbf{r}_S$ and $\textbf{w}_{\textbf{k}_*}$ vanishes precisely to first order both at 0 and $\textbf{r}_S.$ This already implies the second line in (8.15), since the off-diagonal terms in the matrix vanish, and that $(\Vert \textbf{w}_{\textbf{k}_*}(\textbf{r})\Vert ^2 - \Vert \textbf{v}_{\textbf{k}_*}(\textbf{r})\Vert ^2)$ is non-zero. Thus, it remains to show that $\langle \textbf{w}_{\textbf{k}_*}(\textbf{r}'), \textbf{v}_{\textbf{k}_*}(\textbf{r}')\rangle$ is non-zero. We can write, following (8.13), up to a negligible phase $\textbf{w}_{\textbf{k}_*}(\textbf{r}') = \wp (x_1'+\vert r_S\vert +ix_2') \textbf{v}_{\textbf{k}_*}(\textbf{r}')$ and thus

$$\begin{aligned}\langle \textbf{w}_{\textbf{k}_*}(\textbf{r}'), \textbf{v}_{\textbf{k}_*}(\textbf{r}')\rangle = \wp (x_1' +ix_2'+\vert r_S\vert ) \Vert \textbf{v}_{\textbf{k}_*}(\textbf{r}')\Vert ^2\end{aligned}$$

and since $\wp (x_1' +ix_2'+\vert r_S\vert )$ has the same zeros as $\textbf{w}_{\textbf{k}_*}$ and $\textbf{v}_{\textbf{k}_*}(\textbf{r}') \ne 0$, the claim follows. $\square$

By [23, Theorem 4] the four flat bands are uniformly gapped from the remaining bands in TBG-4. Thus, Corollary 2.1 shows that the spectral projection onto the four flat bands is real-analytic in $(k_x,k_y).$ Therefore, combining Propositions 8.3, 8.4 with Corollary 9, we have the following result

#### [Style2 Style2]Theorem 11

The ferromagnetic Slater determinants states are the unique ground states of the corresponding flat-band interacting model for TBG-4.

### Application of main theorem to eTTG-4

Recall the definition of the $\times$ operation in Eqs. 3.16. If we denote by $\textbf{u}_{{\textbf{0}}}\in L^2_{2,2}$ the flat band eigenfunction associated in the nullspace of $D_{\text {TBG}}(\alpha _0)$, then

$$\begin{aligned} \textbf{w}_{{\textbf{0}}}:= \textbf{u}_{{\textbf{0}}} \times \textbf{u}_{{\textbf{0}}} \in \ker _{L^2_{1,1}}(D_{\text {eTTG}}(\sqrt{2}\alpha _0)).\end{aligned}$$

We then have that, since ${\mathcal {L}}u(\textbf{r})=\overline{u(-\textbf{r})}=\pm u(\textbf{r}),$

$$\begin{aligned} \Vert \textbf{w}_{{\textbf{0}}}(\textbf{r})\Vert = \Vert \textbf{w}_{{\textbf{0}}}(-\textbf{r})\Vert .\end{aligned}$$

In particular, since $\textbf{u}_{{\textbf{0}}}$ has a simple zero at $\textbf{r}=0$, $\textbf{w}_{{\textbf{0}}}$ has a double zero at $\textbf{r}=0$.

The two *A*-sublattice polarized flat band eigenfunctions of eTTG-4 are given by

$$\begin{aligned} \textbf{w}_{\textbf{k}}(\textbf{r})=F_{\textbf{k}}(\textbf{r}) \textbf{w}_0(\textbf{r}) \text { and } \quad \textbf{w}'_{\textbf{k}}(\textbf{r})=F_{\textbf{k}/2}(\textbf{r})F_{\textbf{k}/2}(\textbf{r}) \textbf{w}_0(\textbf{r}) \end{aligned}$$

for all $\textbf{k}$ for which the zeros of $\textbf{w}_{\textbf{k}}$ and $\textbf{w}'_{\textbf{k}}$ do not coincide. We find that $\textbf{w}_{\textbf{k}}$ vanishes in the fundamental domain of the lattice at $\textbf{r}=0$ and also at $\textbf{r}=[x_1,x_2]^{\top }$ with $x_1+ix_2 =- \frac{4\pi i (k_1+ik_2)}{3\sqrt{3}}.$ On the other hand, the function $\textbf{w}_{\textbf{k}}'$ has a double zero at $x_1+ix_2 =- \frac{4\pi i (k_1+ik_2)}{6\sqrt{3}}.$ Consequently, the functions $\textbf{w}_{\textbf{k}}$ and $\textbf{w}_{\textbf{k}}'$ span a two-dimensional vector space for all $\textbf{k}\in \Omega$ for which the zeros do not coalesce. However, for $\textbf{k}={\textbf{0}}$ the two functions coincide. Thus, we may choose in a neighbourhood of $\textbf{k}={\textbf{0}}$ a different representation, by considering

$$\begin{aligned} \textbf{w}''_{\textbf{k}}({\textbf{r}})= F_{\textbf{k}+\textbf{q}_1}(\textbf{r})F_{-\textbf{q}_1}(\textbf{r})\textbf{w}_{{\textbf{0}}}(\textbf{r}),\end{aligned}$$

which for $\textbf{k}={\textbf{0}}$ has zeros at $\pm {\textbf{r}}_S$ and is thus linearly independent of $\textbf{w}_{\textbf{k}}$ in a neighbourhood of $\textbf{k}=0$. It is clear that $\textbf{w}_{\textbf{k}}''$ is independent of $\textbf{w}_{\textbf{k}}$, since they have different zeros. It is also clear that $\textbf{w}_{\textbf{k}}''$ satisfies $(D(\alpha )+\textbf{k})\textbf{w}''_{\textbf{k}}=0$, using that $(2D_{{\bar{z}}}+\textbf{k}_0) F_{\textbf{k}_0}=c(\textbf{k}_0)\delta (\textbf{r}),$ where $c(\textbf{k}_0)$ is a normalizing constant since

$$\begin{aligned} \begin{aligned}(D(\alpha )+\textbf{k})\textbf{w}''_{\textbf{k}}&= (2D_{{\bar{z}}}+\textbf{k}+\textbf{q}_1)(F_{\textbf{k}+\textbf{q}_1}(\textbf{r})) F_{-\textbf{q}_1}(\textbf{r})\textbf{w}_{{\textbf{0}}}(\textbf{r}) \\&\hspace{2em} + (2D_{{\bar{z}}}-\textbf{q}_1)(F_{-\textbf{q}_1}(\textbf{r}))F_{\textbf{k}+\textbf{q}_1}(\textbf{r})\textbf{w}_{{\textbf{0}}}(\textbf{r}) \\&\hspace{2em} + D(\alpha )(\textbf{w}_{{\textbf{0}}}(\textbf{r})) F_{\textbf{k}+\textbf{q}_1}(\textbf{r})F_{-\textbf{q}_1}(\textbf{r}) \\&= c(\textbf{k}+ \textbf{q}_1) \delta (\textbf{r}) F_{-\textbf{q}_1}(\textbf{r})\textbf{w}_{{\textbf{0}}}(\textbf{r}) \\&\hspace{2em} + c(-\textbf{q}_1) \delta (\textbf{r}) F_{\textbf{k}+\textbf{q}_1}(\textbf{r})\textbf{w}_{{\textbf{0}}}(\textbf{r}) \\&\hspace{2em} + D(\alpha )(\textbf{w}_{{\textbf{0}}}(\textbf{r})) F_{\textbf{k}+\textbf{q}_1}(\textbf{r})F_{-\textbf{q}_1}(\textbf{r}) = 0. \end{aligned} \end{aligned}$$

The first two terms vanish since $F_{\textbf{k}_0}$ has a first order pole at $\textbf{r}= \varvec{0}$ but $\textbf{w}_{{\textbf{0}}}$ vanishes at $\textbf{r}= \varvec{0}$ to second order and the last term vanishes by construction.

Thus, we can always find a cover of open neighbourhoods $U_i$, where $\textbf{w}_{\textbf{k}}$ and some other function ${\tilde{\textbf{w}}}_{\textbf{k},i}$ are linearly independent and depend analytically on $\textbf{k}.$ We can associate a spectral projection from these two states defined as $\Pi _{i}(\textbf{k}):=M_i(\textbf{k}) (M_i(\textbf{k})^{\dag } M_i(\textbf{k}))^{-1}M_i(\textbf{k})^{\dag }$ with $M_i(\textbf{k})$ having the following column vectors

$$\begin{aligned} [\textbf{w}_{\textbf{k}},0]^{\top }, [{\tilde{\textbf{w}}}_{\textbf{k},i},0]^{\top },[0, {\mathcal {Q}}\textbf{w}_{\textbf{k}}]^{\top }, [0, \mathcal {Q}{\tilde{\textbf{w}}}_{\textbf{k},i}]^{\top }, \end{aligned}$$

where we have used that $\mathcal {Q}D(\alpha )\mathcal {Q} = D(\alpha )^{\dagger }$. Since $\textbf{w}_{\textbf{k}}$ and ${\tilde{\textbf{w}}}_{\textbf{k},i}$ are analytic on $U_i$ and $\mathcal {Q}$ is antiunitary, $M_{i}(\textbf{k})$ is real analytic on each $U_{i}$. Let $\rho _i$ be a partition of unity with ${{\,\textrm{supp}\,}}(\rho _i) \subset U_i,$ then $\Pi (\textbf{k}):=\sum _i \rho _i \Pi _i(\textbf{k})$ is a smooth projection in $\textbf{k}$ projecting onto the flat band Bloch functions with quasi-momentum $\textbf{k}$ as was claimed.

Since $\textbf{w}_{{\textbf{0}}}$ has therefore the same properties as $\textbf{w}_{{\textbf{0}}}$ in the proof of Proposition 8.3. One can then replicate the proofs of Propositions 8.3 and 8.4 to find that

#### [Style2 Style2]Proposition 8.5

Let $\alpha \in {\mathbb {C}}$ be a two-fold degenerate magic angle of eTTG-4, with $\textbf{k}_*$ as in Proposition 8.4, there are $\textbf{G}\in \Gamma ^*$ such that

8.16 $$\begin{aligned} \operatorname {Im}{{\,\textrm{tr}\,}}(A_{\textbf{k}_*}(\textbf{G}))\ne 0 \end{aligned}$$

while for $\textbf{k}\in \Gamma ^*$ one has

8.17 $$\begin{aligned} \operatorname {Im}{{\,\textrm{tr}\,}}(A_{\textbf{k}}(\textbf{G}))=0 \text { for all }\textbf{G}\in \Gamma ^*. \end{aligned}$$

#### [Style2 Style2]Proposition 8.6

Let $\alpha$ be a two-fold degenerate magic angle of eTTG-4 and $\textbf{k}= \pm e_2$. Moreover, let $\textbf{r}\in \{{\textbf{0}},\pm \textbf{r}_S\}$ and $\textbf{r}' \notin \{{\textbf{0}},\pm \textbf{r}_S\} +\Gamma$, then the expression

8.18 $$\begin{aligned} \begin{aligned} D(\textbf{r}, \textbf{r}')&:= \det { \begin{bmatrix} \Vert \textbf{w}_{\textbf{k}}(\textbf{r})\Vert ^2 - \Vert \textbf{v}_{\textbf{k}}(\textbf{r})\Vert ^2 &  \langle \textbf{w}_{\textbf{k}}(\textbf{r}), \textbf{v}_{\textbf{k}}(\textbf{r})\rangle \\ \Vert \textbf{w}_{\textbf{k}}(\textbf{r}')\Vert ^2 - \Vert \textbf{v}_{\textbf{k}}(\textbf{r}')\Vert ^2 &  \langle \textbf{w}_{\textbf{k}}(\textbf{r}'), \textbf{v}_{\textbf{k}}(\textbf{r}')\rangle \end{bmatrix}} \\&= (\Vert \textbf{w}_{\textbf{k}}(\textbf{r})\Vert ^2 - \Vert \textbf{v}_{\textbf{k}}(\textbf{r})\Vert ^2) \langle \textbf{w}_{\textbf{k}}(\textbf{r}'), \textbf{v}_{\textbf{k}}(\textbf{r}')\rangle \end{aligned} \end{aligned}$$

is non-zero.

Therefore, combining Propositions 8.5, 8.6 with Corollary 9, we have the following result

#### [Style2 Style2]Theorem 12

The ferromagnetic Slater determinants states are the unique ground states of the corresponding flat-band interacting model for eTTG-4.

## Data Availability

All figures in this manuscript were generated in MATLAB. Figures and code are available upon request

## Proof of Lemma 4.1

We start by proving that $\Lambda _{\textbf{k}}(\textbf{0}) = I$ for all $\textbf{k} \in \Omega$. Since $\Lambda _{\textbf{k}}(\textbf{G})$ are the Fourier coefficients of function $\rho _{\textbf{k}, \textbf{k}}(\textbf{r})$ (see Eq. (2.17)) we have that

A.1 $$\begin{aligned} \begin{aligned} \,[\Lambda _{\textbf{k}}(\textbf{0})]_{mn}&= \int _{\Omega } e^{-i \textbf{0} \cdot \textbf{r}} [\rho _{\textbf{k}, \textbf{k}}(\textbf{r})]_{mn} \,\textrm{d}\textbf{r} \\&= \int _{\Omega } \sum _{\sigma , j}  \overline{u_{m \textbf{k}}(\textbf{r}; \sigma , j)} u_{n \textbf{k}}(\textbf{r}; \sigma , j) \,\textrm{d}\textbf{r} \\&= \delta _{mn} \end{aligned} \end{aligned}$$

where the last line is due to the fact $u_{m \textbf{k}}$ are orthogonal eigenfunctions of $H_{\textbf{k}}$.

To prove the lower bound on $\Lambda _{\textbf{k}}(\textbf{q}') \Lambda _{\textbf{k}}(\textbf{q}')^{\dagger }$, we will show there exists a *C* so that for all $\textbf{k}$ and all $\textbf{q}'$

A.2 $$\begin{aligned} \Vert \Lambda _{\textbf{k}}(\textbf{q}') \Lambda _{\textbf{k}}(\textbf{q}')^{\dagger } - \Lambda _{\textbf{k}}(\textbf{0}) \Lambda _{\textbf{k}}(\textbf{0})^{\dagger } \Vert \le C |\textbf{q}'| \end{aligned}$$

this implies the lemma by Weyl’s eigenvalue inequality.

We begin by introducing bra-ket notation on the space $L^{2}(\Gamma ^{*} \otimes {\mathbb {C}}^{2} \otimes {\mathbb {C}}^{N})$. Using bra-ket notation the function ${\hat{u}}_{m \textbf{k}}(\textbf{G}; \sigma , j)$ can be written as

A.3 $$\begin{aligned} \sum _{\textbf{G}, \sigma , j}  {\hat{u}}_{m \textbf{k}}(\textbf{G}; \sigma , j) \vert \textbf{G}, \sigma , j\rangle \end{aligned}$$

where the ket vectors $\vert \textbf{G}, \sigma , j\rangle$ form an orthonormal basis for $L^{2}(\Gamma ^{*} \otimes {\mathbb {C}}^{2} \otimes {\mathbb {C}}^{N})$. We also let $\{ \vert m\rangle : m \in {\mathcal {N}} \}$ denote an orthonormal basis for ${\mathbb {C}}^{\# | {\mathcal {N}} |}$. With this notation, for each any $\textbf{k} \in {\mathbb {R}}^{2}$ we define

A.4 $$\begin{aligned} \Phi _{\textbf{k}}:= \frac{1}{|\Omega |^{\frac{1}{2}}} \sum _{m \in {\mathcal {N}}}  \sum _{\textbf{G}, \sigma , j}  {\hat{u}}_{m \textbf{k}}(\textbf{G}; \sigma , j) \vert \textbf{G}, \sigma , j\rangle \langle m\vert . \end{aligned}$$

In other words, the operator $\Phi _{\textbf{k}}$ is an infinite matrix where the *m*th column is the function ${\hat{u}}_{m \textbf{k}}(\textbf{G}; \sigma , j)$. Recalling the definition of the form factor Eq. (2.18) we can now see the connection with $\Phi _{\textbf{k}}$:

A.5 $$\begin{aligned} [\Lambda _{\textbf{k}}(\textbf{q}')]_{mn} = \frac{1}{|\Omega |} \sum _{\textbf{G}',\sigma ,j} \overline{{\hat{u}}_{m\textbf{k}}(\textbf{G}'; \sigma , j)} {\hat{u}}_{n(\textbf{k}+\textbf{q}')}(\textbf{G}'; \sigma , j) = \Phi _{\textbf{k}}^{\dagger } \Phi _{\textbf{k} + \textbf{q}'}. \end{aligned}$$

Therefore, with this notation

A.6 $$\begin{aligned} \begin{aligned} \Lambda _{\textbf{k}}(\textbf{q}')\Lambda _{\textbf{k}}(\textbf{q}')^{\dagger } - \Lambda _{\textbf{k}}(\textbf{0})\Lambda _{\textbf{k}}(\textbf{0})^{\dagger }&= \Phi _{\textbf{k}}^{\dagger } \Phi _{\textbf{k} + \textbf{q}'} \Phi _{\textbf{k} + \textbf{q}'}^{\dagger } \Phi _{\textbf{k}} - \Phi _{\textbf{k}}^{\dagger } \Phi _{\textbf{k}} \Phi _{\textbf{k}}^{\dagger } \Phi _{\textbf{k}} \\&= \Phi _{\textbf{k}}^{\dagger } \Big ( \Phi _{\textbf{k} + \textbf{q}'} \Phi _{\textbf{k} + \textbf{q}'}^{\dagger } - \Phi _{\textbf{k}} \Phi _{\textbf{k}}^{\dagger } \Big ) \Phi _{\textbf{k}}. \end{aligned} \end{aligned}$$

Since $$\Vert \Phi _{\textbf{k}} \Vert = \Vert \Phi _{\textbf{k}}^{\dagger } \Vert = 1$$

A.7 $$\begin{aligned} \Vert \Lambda _{\textbf{k}}(\textbf{q}')\Lambda _{\textbf{k}}(\textbf{q}')^{\dagger } - \Lambda _{\textbf{k}}(\textbf{0})\Lambda _{\textbf{k}}(\textbf{0})^{\dagger } \Vert \le \Vert \Phi _{\textbf{k} + \textbf{q}'} \Phi _{\textbf{k} + \textbf{q}'}^{\dagger } - \Phi _{\textbf{k}} \Phi _{\textbf{k}}^{\dagger } \Vert . \end{aligned}$$

By the definition of $\Phi _{\textbf{k}}$, we can compute

A.8 $$\begin{aligned} \Phi _{\textbf{k}} \Phi _{\textbf{k}}^{\dagger } = \frac{1}{|\Omega |} \sum _{m \in {\mathcal {N}}}  \sum _{\textbf{G}, \sigma , j}  \sum _{\textbf{G}', \sigma ', j'}  {\hat{u}}_{m \textbf{k}}(\textbf{G}; \sigma , j) \overline{{\hat{u}}_{m \textbf{k}}(\textbf{G}'; \sigma ', j')} \vert \textbf{G}, \sigma , j\rangle \langle \textbf{G}', \sigma ', j'\vert .\nonumber \\ \end{aligned}$$

Comparing this expression to the kernel for the flat band spectral projector $\Pi (\textbf{k})$

A.9 $$\begin{aligned} \Pi (\textbf{k})( (\textbf{r}, \sigma , j), (\textbf{r}', \sigma ', j')) = \sum _{m \in {\mathcal {N}}}  u_{m \textbf{k}}(\textbf{r}; \sigma , j) \overline{u_{m \textbf{k}}(\textbf{r}'; \sigma ', j')} \end{aligned}$$

we see that $\Phi _{\textbf{k}} \Phi _{\textbf{k}}^{\dagger }$ is simply the Fourier transform of $\Pi (\textbf{k})$ with respect to $\textbf{r}$. Explicitly, let ${\mathcal {F}}: L^{2}(\Omega \otimes {\mathbb {C}}^{2} \otimes {\mathbb {C}}^{N}) \rightarrow L^{2}(\Gamma ^{*} \otimes {\mathbb {C}}^{2} \otimes {\mathbb {C}}^{N})$ denote the Fourier transform from $L^{2}(\Omega ) \rightarrow L^{2}(\Gamma ^{*})$ only acting on the first variable:

A.10 $$\begin{aligned} {\mathcal {F}}(f)(\textbf{G}; \sigma , j) = \int _{\Omega } e^{-i \textbf{G} \cdot \textbf{r}} f(\textbf{r}; \sigma , j) \,\textrm{d}\textbf{r}. \end{aligned}$$

From this definition, one easily checks that ${\mathcal {F}} {\mathcal {F}}^{\dagger } = \frac{1}{|\Omega |} I$. For any $f \in L^{2}(\Omega \otimes {\mathbb {C}}^{2} \otimes {\mathbb {C}}^{N})$ we have

A.11 $$\begin{aligned} \begin{aligned} {\mathcal {F}}(\Pi (\textbf{k}) f)(\textbf{G}; \sigma , j)&= \sum _{m \in {\mathcal {N}}}  \langle u_{m \textbf{k}}, f\rangle {\mathcal {F}}(u_{m \textbf{k}})(\textbf{G}; \sigma , j) \\&= \frac{1}{|\Omega |} \sum _{m \in {\mathcal {N}}}  \langle {\mathcal {F}} (u_{m \textbf{k}}), {\mathcal {F}}(f)\rangle {\mathcal {F}}(u_{m \textbf{k}})(\textbf{G}; \sigma , j) \\&= (\Phi _{\textbf{k}} \Phi _{\textbf{k}}^{\dagger })({\mathcal {F}}(f)) \end{aligned} \end{aligned}$$

which proves $\Phi _{\textbf{k}} \Phi _{\textbf{k}}^{\dagger } = {\mathcal {F}} \Pi (\textbf{k}) {\mathcal {F}}^{-1}$. Therefore,

A.12 $$\begin{aligned} \Vert \Phi _{\textbf{k} + \textbf{q}'} \Phi _{\textbf{k} + \textbf{q}'}^{\dagger } - \Phi _{\textbf{k}} \Phi _{\textbf{k}}^{\dagger } \Vert = \Vert {\mathcal {F}} \Big ( \Pi (\textbf{k} + \textbf{q}') - \Pi (\textbf{k}) \Big ) {\mathcal {F}}^{-1} \Vert = \Vert \Pi (\textbf{k} + \textbf{q}') - \Pi (\textbf{k}) \Vert .\nonumber \\ \end{aligned}$$

Since $\Pi (\textbf{k})$ is Lipschitz by assumption, this implies

A.13 $$\begin{aligned} \Vert \Lambda _{\textbf{k}}(\textbf{q}')\Lambda _{\textbf{k}}(\textbf{q}')^{\dagger } - \Lambda _{\textbf{k}}(\textbf{0})\Lambda _{\textbf{k}}(\textbf{0})^{\dagger } \Vert \le L | \textbf{q}' | \end{aligned}$$

where *L* is the Lipschitz constant for $\Pi$. Hence by the previous reasoning, the lemma is proved.

## Derivation of Hartree and Fock Energies for FBI Hamiltonians

To begin our derivation, we start by taking the formula for ${\hat{H}}_{\textrm{FBI}}$ (Eq. (2.25)), multiplying both sides by $N_{\textbf{k}} |\Omega |$, and expanding the product $\rho (\textbf{q}') \rho (-\textbf{q}')$ into a sum of products of creation and annihilation operators. With this expansion, $N_{\textbf{k}} |\Omega | {\hat{H}}_{\textrm{FBI}}$ can be written as a sum of four terms:

B.1 $$\begin{aligned} N_{\textbf{k}} |\Omega | {\hat{H}}_{\textrm{FBI}} = {\hat{H}}_{1} + {\hat{H}}_{2} + {\hat{H}}_{3} + C \end{aligned}$$

where

B.2 $$\begin{aligned} {\hat{H}}_{1}&= \sum _{\textbf{q}'}  \sum _{\textbf{k}, \textbf{k}'}  \sum _{mnm'n'}  {\hat{V}}(\textbf{q}') [\Lambda _{\textbf{k}}(\textbf{q}')]_{mn} [\Lambda _{\textbf{k}'}(-\textbf{q}')]_{m'n'} {\hat{f}}_{m \textbf{k}}^{\dagger } {\hat{f}}_{n (\textbf{k} + \textbf{q}')} {\hat{f}}_{m' \textbf{k}'}^{\dagger } {\hat{f}}_{n' (\textbf{k}' - \textbf{q}')}, \end{aligned}$$

B.3 $$\begin{aligned} {\hat{H}}_{2}&= -\frac{1}{2} \sum _{\textbf{G} \in \Gamma ^{*}}  \sum _{\textbf{k}, \textbf{k}'}  \sum _{m'n'}  {\hat{V}}(\textbf{G}) {{\,\textrm{tr}\,}}{\Big (\Lambda _{\textbf{k}}(\textbf{G})\Big )} [\Lambda _{\textbf{k}'}(-\textbf{G})]_{m'n'} {\hat{f}}_{m' \textbf{k}'}^{\dagger } {\hat{f}}_{n' \textbf{k}'}, \end{aligned}$$

B.4 $$\begin{aligned} {\hat{H}}_{3}&= -\frac{1}{2} \sum _{\textbf{G} \in \Gamma ^{*}}  \sum _{\textbf{k}, \textbf{k}'}  \sum _{mn}  {\hat{V}}(\textbf{G}) [\Lambda _{\textbf{k}}(\textbf{G})]_{mn} {{\,\textrm{tr}\,}}{\Big (\Lambda _{\textbf{k}'}(-\textbf{G})\Big )} {\hat{f}}_{m \textbf{k}}^{\dagger } {\hat{f}}_{n \textbf{k}}, \end{aligned}$$

B.5 $$\begin{aligned} C&= \frac{1}{4} \sum _{\textbf{G} \in \Gamma ^{*}}  \sum _{\textbf{k}, \textbf{k}'}  {\hat{V}}(\textbf{G}) {{\,\textrm{tr}\,}}{\Big (\Lambda _{\textbf{k}}(\textbf{G})\Big )} {{\,\textrm{tr}\,}}{\Big (\Lambda _{\textbf{k}'}(-\textbf{G})\Big )}. \end{aligned}$$

where we have used that that by definition ${\hat{f}}_{m (\textbf{k} + \textbf{G})} = {\hat{f}}_{m \textbf{k}}$ for all $\textbf{G} \in \Gamma ^{*}$.

To apply Wick’s theorem, we normal order the creation and annihilation operators to write ${\hat{H}}_{1}$ as a sum of two terms ${\hat{H}}_{1} = {\hat{H}}_{1a} + {\hat{H}}_{1b}$ where

B.6 $$\begin{aligned} \begin{aligned} {\hat{H}}_{1a}&= \sum _{\textbf{q}'}  \sum _{\textbf{k}, \textbf{k}'}  \sum _{mnm'n'}  {\hat{V}}(\textbf{q}') [\Lambda _{\textbf{k}}(\textbf{q}')]_{mn} [\Lambda _{\textbf{k}'}(-\textbf{q}')]_{m'n'} {\hat{f}}_{m \textbf{k}}^{\dagger } {\hat{f}}_{m' \textbf{k}'}^{\dagger } {\hat{f}}_{n' (\textbf{k}' - \textbf{q}')} {\hat{f}}_{n (\textbf{k} + \textbf{q}')}, \\ {\hat{H}}_{1b}&= \sum _{\textbf{q}'}  \sum _{\textbf{k}}  \sum _{m n}  {\hat{V}}(\textbf{q}') [\Lambda _{\textbf{k}}(\textbf{q}') \Lambda _{\textbf{k} + \textbf{q}'}(-\textbf{q}')]_{m n'} {\hat{f}}_{m \textbf{k}}^{\dagger } {\hat{f}}_{n' \textbf{k}}. \end{aligned} \end{aligned}$$

We recall that any translation invariant many-body state, $\vert \Phi \rangle$, the one-body reduced density matrix (1-RDM) is defined for all $\textbf{k}, \textbf{k}' \in \Omega$ as $[P(\textbf{k}, \textbf{k}')]_{nm}:= \delta _{\textbf{k}, \textbf{k}'} \langle \Psi | {\hat{f}}_{m \textbf{k}'}^{\dagger } {\hat{f}}_{n \textbf{k}} | \Psi \rangle$. Since for translation invariant many-body states the 1-RDM vanishes if $\textbf{k} \ne \textbf{k}'$, we will slightly abuse notation and define $P(\textbf{k}):= P(\textbf{k}, \textbf{k})$. Due to the periodic boundary conditions on the creation and annihilation operators (Eq. (2.15)) the 1-RDM $P(\textbf{k})$ can be defined for $\textbf{k} \in {\mathbb {R}}^{2}$, by satisfying the periodic boundary conditions:

B.7 $$\begin{aligned} P(\textbf{k} + \textbf{G}) = P(\textbf{k}) \quad \text {for any} \quad \textbf{G} \in \Gamma ^{*} \end{aligned}$$

We finally note that by definition, the 1-RDM is a self-adjoint operator for all $\textbf{k}$, $P(\textbf{k})^{\dagger } = P(\textbf{k})$.

For a translation invariant Slater determinant state $\vert \Psi \rangle$, we can easily evaluate the energy of ${\hat{H}}_{1b}$, ${\hat{H}}_{2}$, and ${\hat{H}}_{3}$ in terms of the 1-RDM

B.8 $$\begin{aligned} \langle \Psi | {\hat{H}}_{1b} | \Psi \rangle&= \sum _{\textbf{q}'}  \sum _{\textbf{k}}  {\hat{V}}(\textbf{q}') {{\,\textrm{tr}\,}}{\Big (\Lambda _{\textbf{k}}(\textbf{q}') \Lambda _{\textbf{k} + \textbf{q}'}(-\textbf{q}') P(\textbf{k})\Big )} \end{aligned}$$

B.9 $$\begin{aligned} \langle \Psi | {\hat{H}}_{2} | \Psi \rangle&= -\frac{1}{2}\sum _{\textbf{G} \in \Gamma ^{*}}  \sum _{\textbf{k}, \textbf{k}'}  {\hat{V}}(\textbf{G}) {{\,\textrm{tr}\,}}{\Big (\Lambda _{\textbf{k}}(\textbf{G})\Big )} {{\,\textrm{tr}\,}}{\Big (\Lambda _{\textbf{k}'}(-\textbf{G}) P(\textbf{k}')\Big )} \end{aligned}$$

B.10 $$\begin{aligned} \langle \Psi | {\hat{H}}_{3} | \Psi \rangle&= -\frac{1}{2}\sum _{\textbf{G} \in \Gamma ^{*}}  \sum _{\textbf{k}, \textbf{k}'}  {\hat{V}}(\textbf{G}) {{\,\textrm{tr}\,}}{\Big (\Lambda _{\textbf{k}}(\textbf{G}) P(\textbf{k})\Big )} {{\,\textrm{tr}\,}}{\Big (\Lambda _{\textbf{k}'}(-\textbf{G})\Big )} \end{aligned}$$

To evaluate the remaining contribution to the energy, we appeal to Wick’s theorem [47, Section 3.3]

B.11 $$\begin{aligned} \begin{aligned}&\langle \Psi | {\hat{f}}_{m \textbf{k}}^{\dagger } {\hat{f}}_{m' \textbf{k}'}^{\dagger } {\hat{f}}_{n' (\textbf{k}' - \textbf{q}')} {\hat{f}}_{n (\textbf{k} + \textbf{q}')} | \Psi \rangle = \left( \sum _{\textbf{G} \in \Gamma ^{*}}  \delta _{\textbf{q}', \textbf{G}} \right) [ P(\textbf{k})]_{nm} [P(\textbf{k}') ]_{n' m'} \\&\hspace{2em} - \left( \sum _{\textbf{G} \in \Gamma ^{*}} \delta _{\textbf{k} - \textbf{k}' + \textbf{q}', \textbf{G}} \right) [P(\textbf{k})]_{n' m} [P(\textbf{k} + \textbf{q}')]_{n m'} \end{aligned} \end{aligned}$$

where the additional sums over $\Gamma ^{*}$ are due to the periodic boundary condition Eq. (B.7). Therefore, for a translation invariant Slater determinant state $\vert \Psi \rangle$ we have

B.12 $$\begin{aligned} \begin{aligned} \langle \Psi | {\hat{H}}_{1a} | \Psi \rangle&= \sum _{\textbf{G} \in \Gamma ^{*}}  \sum _{\textbf{k}, \textbf{k}'}  {\hat{V}}(\textbf{G}) {{\,\textrm{tr}\,}}{\Big (\Lambda _{\textbf{k}}(\textbf{G}) P(\textbf{k})\Big )} {{\,\textrm{tr}\,}}{\Big (\Lambda _{\textbf{k}'}(-\textbf{G}) P(\textbf{k}') \Big )} \\&\hspace{2em} - \sum _{\textbf{q}'}  \sum _{\textbf{k}}  {\hat{V}}(\textbf{q}') {{\,\textrm{tr}\,}}{\Big (\Lambda _{\textbf{k}}(\textbf{q}') P(\textbf{k} + \textbf{q}') \Lambda _{\textbf{k} + \textbf{q}'}(-\textbf{q}') P(\textbf{k})\Big )} \end{aligned} \end{aligned}$$

Hence, for a translation invariant Slater determinant, the Hartree-Fock energy of this state is given by the sum of Eq. (B.5),(B.8),(B.9),(B.10),(B.12).

Looking at these sums, we notice that there are 4 terms which sum over $\textbf{G} \in \Gamma ^{*}$ and 2 terms which sum over $\textbf{q}' \in {\mathcal {K}} + \Gamma ^{*}$. By completing the square, the sums over $\textbf{G} \in \Gamma ^{*}$ can be combined into a single expression

B.13 $$\begin{aligned} \sum _{\textbf{G} \in \Gamma ^{*}}  \sum _{\textbf{k}, \textbf{k}'}  {\hat{V}}(\textbf{G}) {{\,\textrm{tr}\,}}{\left( \Lambda _{\textbf{k}}(\textbf{G}) \Big (P(\textbf{k}) - \frac{1}{2} I\Big ) \right) } {{\,\textrm{tr}\,}}{\left( \Lambda _{\textbf{k}'}(-\textbf{G}) \Big ( P(\textbf{k}') - \frac{1}{2} I \Big ) \right) }. \end{aligned}$$

Since $\Lambda _{\textbf{k}}(\textbf{G})^{\dagger } = \Lambda _{\textbf{k}}(-\textbf{G})$ (Eq. (2.21)) and $P(\textbf{k})$ is self-adjoint this can be further simplified

B.14 $$\begin{aligned} \sum _{\textbf{G} \in \Gamma ^{*}}  {\hat{V}}(\textbf{G}) \left| \sum _{\textbf{k}}  {{\,\textrm{tr}\,}}{\left( \Lambda _{\textbf{k}}(\textbf{G}) \Big (P(\textbf{k}) - \frac{1}{2} I\Big ) \right) } \right| ^{2} \end{aligned}$$

which is precisely the Hartree energy (Eq. (5.6)) up to a constant factor after replacing $Q(\textbf{k}) = 2 P(\textbf{k}) - I = 2 (P(\textbf{k}) - \frac{1}{2} I)$.

This leaves us with two additional sums over $\textbf{q}'$

B.15 $$\begin{aligned} \begin{aligned}&- \sum _{\textbf{q}'}  \sum _{\textbf{k}}  {\hat{V}}(\textbf{q}') {{\,\textrm{tr}\,}}{\Big (\Lambda _{\textbf{k}}(\textbf{q}') P(\textbf{k} + \textbf{q}') \Lambda _{\textbf{k} + \textbf{q}'}(-\textbf{q}') P(\textbf{k})\Big )} \\&\hspace{2em} + \sum _{\textbf{q}'}  \sum _{\textbf{k}}  {\hat{V}}(\textbf{q}') {{\,\textrm{tr}\,}}{\Big (\Lambda _{\textbf{k}}(\textbf{q}') \Lambda _{\textbf{k} + \textbf{q}'}(-\textbf{q}') P(\textbf{k})\Big )}. \end{aligned} \end{aligned}$$

Since the first term is quadratic in *P* and the second term is linear in *P* to match the form given in Eq. (5.7), we will once again complete the square. As part of completing the square, we use the following identity which follows from performing the change of variables^3^$(\textbf{k}, \textbf{q}') \mapsto (\textbf{k} + \textbf{q}', -\textbf{q}')$ and the cyclic property of trace:

B.16 $$\begin{aligned} \begin{aligned}&\sum _{\textbf{q}'}  \sum _{\textbf{k}}  {\hat{V}}(\textbf{q}') {{\,\textrm{tr}\,}}{\Big (\Lambda _{\textbf{k}}(\textbf{q}') \Lambda _{\textbf{k} + \textbf{q}'}(-\textbf{q}') P(\textbf{k})\Big )} \\&\hspace{2em} = \sum _{\textbf{q}'}  \sum _{\textbf{k}}  {\hat{V}}(\textbf{q}') {{\,\textrm{tr}\,}}{\Big (\Lambda _{\textbf{k}}(\textbf{q}') P(\textbf{k} + \textbf{q}) \Lambda _{\textbf{k} + \textbf{q}'}(-\textbf{q}') \Big )}. \end{aligned} \end{aligned}$$

Using Eq. (B.16), we can write Eq. (B.15) as follows

B.17 $$\begin{aligned}  &   - \sum _{\textbf{q}'}  \sum _{\textbf{k}}  {\hat{V}}(\textbf{q}') {{\,\textrm{tr}\,}}{\Big (\Lambda _{\textbf{k}}(\textbf{q}') P(\textbf{k} + \textbf{q}') \Lambda _{\textbf{k} + \textbf{q}'}(-\textbf{q}') P(\textbf{k})\Big )} \nonumber \\  &   \hspace{2em} + \frac{1}{2} \sum _{\textbf{q}'}  \sum _{\textbf{k}}  {\hat{V}}(\textbf{q}') {{\,\textrm{tr}\,}}{\Big (\Lambda _{\textbf{k}}(\textbf{q}') \Lambda _{\textbf{k} + \textbf{q}'}(-\textbf{q}') P(\textbf{k})\Big )} \nonumber \\  &   \hspace{2em} + \frac{1}{2} \sum _{\textbf{q}'}  \sum _{\textbf{k}}  {\hat{V}}(\textbf{q}') {{\,\textrm{tr}\,}}{\Big (\Lambda _{\textbf{k}}(\textbf{q}') P(\textbf{k} + \textbf{q}') \Lambda _{\textbf{k} + \textbf{q}'}(-\textbf{q}') \Big )} \nonumber \\  &   = - \sum _{\textbf{q}'}  \sum _{\textbf{k}}  {\hat{V}}(\textbf{q}') {{\,\textrm{tr}\,}}{\left( \Lambda _{\textbf{k}}(\textbf{q}') \Big ( P(\textbf{k} + \textbf{q}') - \frac{1}{2} I \Big ) \Lambda _{\textbf{k} + \textbf{q}'}(-\textbf{q}') \Big ( P(\textbf{k}) - \frac{1}{2} I \Big ) \right) } \nonumber \\  &   \hspace{2em} + \sum _{\textbf{q}'}  \sum _{\textbf{k}}  {{\,\textrm{tr}\,}}{\Big ( \Lambda _{\textbf{k}}(\textbf{q}) \Lambda _{\textbf{k} + \textbf{q}'}(-\textbf{q}') \Big )}. \end{aligned}$$

Combining all these calculations together, we conclude that

B.18 $$\begin{aligned} \begin{aligned} \langle \Psi | {\hat{H}}_{\textrm{FBI}} | \Psi \rangle&= \frac{1}{4 N_{\textbf{k}} |\Omega |} \sum _{\textbf{G} \in \Gamma ^{*}}  {\hat{V}}(\textbf{G}) \left| \sum _{\textbf{k}}  {{\,\textrm{tr}\,}}{\left( \Lambda _{\textbf{k}}(\textbf{G}) Q(\textbf{k}) \right) } \right| ^{2} \\&\hspace{2em} - \frac{1}{4 N_{\textbf{k}} |\Omega |} \sum _{\textbf{q}'}  \sum _{\textbf{k}}  {\hat{V}}(\textbf{q}') {{\,\textrm{tr}\,}}{\Big (\Lambda _{\textbf{k}}(\textbf{q}') Q(\textbf{k} + \textbf{q}') \Lambda _{\textbf{k} + \textbf{q}'}(-\textbf{q}') Q(\textbf{k}) \Big )} \\&\hspace{2em} + \frac{1}{N_{\textbf{k}} |\Omega |} \sum _{\textbf{q}'}  \sum _{\textbf{k}}  {{\,\textrm{tr}\,}}{\Big ( \Lambda _{\textbf{k}}(\textbf{q}) \Lambda _{\textbf{k} + \textbf{q}'}(-\textbf{q}') \Big )}. \end{aligned}\nonumber \\ \end{aligned}$$

Note that the last term is a constant depending only of the form factor (and hence independent of $\vert \Psi \rangle$) completing the proof.

## Reformulation of Fock Energy

Using the notations introduced in Eq. (7.12) and using the cyclic property of trace, we have that

C.1 $$\begin{aligned} \begin{aligned}&{{\,\textrm{tr}\,}}( \Lambda _{\textbf{k}}(\textbf{q}') Q(\textbf{k}') \Lambda _{\textbf{k}}(\textbf{q}')^\dagger Q(\textbf{k})) \\&= {{\,\textrm{tr}\,}}\Big ( \begin{bmatrix} B^{(1)}_{\textbf{k}}(\textbf{q}') &  \\ &  B^{(2)}_{\textbf{k}}(\textbf{q}') \end{bmatrix} \begin{bmatrix} c(\textbf{k}') &  s(\textbf{k}') \\ s(\textbf{k}') &  -c(\textbf{k}') \end{bmatrix} \\&\hspace{5em} \begin{bmatrix} B^{(1)}_{\textbf{k}}(\textbf{q}')^{\dagger } &  \\ &  B^{(2)}_{\textbf{k}}(\textbf{q}')^{\dagger } \end{bmatrix} \begin{bmatrix} c(\textbf{k}) &  s(\textbf{k}) \\ s(\textbf{k}) &  -c(\textbf{k}) \end{bmatrix} \Big ). \end{aligned} \end{aligned}$$

We will now split the sine and cosine matrices into their diagonal and off-diagonal parts

C.2 $$\begin{aligned} \begin{bmatrix} c(\textbf{k}) &  s(\textbf{k}) \\ s(\textbf{k}) &  -c(\textbf{k}) \end{bmatrix} = \begin{bmatrix} c(\textbf{k}) &  \\ &  -c(\textbf{k}) \end{bmatrix} + \begin{bmatrix} &  s(\textbf{k}) \\ s(\textbf{k}) &  \end{bmatrix} \end{aligned}$$

and similarly for the matrix involving $c(\textbf{k}')$ and $s(\textbf{k}')$.

Since the form factor is block diagonal, one can easily verify that after splitting sine and cosine matrix as above, the only non-vanishing contributions to the trace are

C.3 $$\begin{aligned} \begin{aligned}&{{\,\textrm{tr}\,}}\Big ( \begin{bmatrix} B^{(1)}_{\textbf{k}}(\textbf{q}') &  \\ &  B^{(2)}_{\textbf{k}}(\textbf{q}') \end{bmatrix} \begin{bmatrix} c(\textbf{k}') &  \\ &  -c(\textbf{k}') \end{bmatrix} \begin{bmatrix} B^{(1)}_{\textbf{k}}(\textbf{q}')^{\dagger } &  \\ &  B^{(2)}_{\textbf{k}}(\textbf{q}')^{\dagger } \end{bmatrix} \begin{bmatrix} c(\textbf{k}) &  \\ &  -c(\textbf{k}) \end{bmatrix} \Big ) \\&{{\,\textrm{tr}\,}}\Big ( \begin{bmatrix} B^{(1)}_{\textbf{k}}(\textbf{q}') &  \\ &  B^{(2)}_{\textbf{k}}(\textbf{q}') \end{bmatrix} \begin{bmatrix} &  s(\textbf{k}') \\ s(\textbf{k}') &  \end{bmatrix} \begin{bmatrix} B^{(1)}_{\textbf{k}}(\textbf{q}')^{\dagger } &  \\ &  B^{(2)}_{\textbf{k}}(\textbf{q}')^{\dagger } \end{bmatrix} \begin{bmatrix} &  s(\textbf{k}) \\ s(\textbf{k}) &  \end{bmatrix} \Big ). \end{aligned} \end{aligned}$$

Therefore,

C.4 $$\begin{aligned} \begin{aligned}&{{\,\textrm{tr}\,}}( \Lambda _{\textbf{k}}(\textbf{q}') Q(\textbf{k}') \Lambda _{\textbf{k}}(\textbf{q}')^\dagger Q(\textbf{k})) \\&= {{\,\textrm{tr}\,}}\Big (\Big [ B^{(1)}_{\textbf{k}}(\textbf{q}') c(\textbf{k}') B^{(1)}_{\textbf{k}}(\textbf{q}')^{\dagger } c(\textbf{k}) + B^{(2)}_{\textbf{k}}(\textbf{q}') c(\textbf{k}') B^{(2)}_{\textbf{k}}(\textbf{q}')^{\dagger } c(\textbf{k}) \Big ] \\&\hspace{3em} + \Big [ B^{(1)}_{\textbf{k}}(\textbf{q}') s(\textbf{k}') B^{(2)}_{\textbf{k}}(\textbf{q}')^{\dagger } s(\textbf{k}) + B^{(2)}_{\textbf{k}}(\textbf{q}') s(\textbf{k}') B^{(1)}_{\textbf{k}}(\textbf{q}')^{\dagger } s(\textbf{k}) \Big ] \Big ). \end{aligned} \end{aligned}$$

The last two terms can be slightly simplified by observing that

C.5 $$\begin{aligned} \Big (B^{(1)}_{{\textbf {k}}}({\textbf {q}}') s({\textbf {k}}') B^{(2)}_{{\textbf {k}}}({\textbf {q}}')^{\dagger } s({\textbf {k}})\Big )^{\dagger } = s({\textbf {k}}) B^{(2)}_{{\textbf {k}}}({\textbf {q}}') s({\textbf {k}}') B^{(1)}_{{\textbf {k}}}({\textbf {q}}')^{\dagger }. \end{aligned}$$

Therefore, using the cyclic property of trace

C.6 $$\begin{aligned} \overline{{{\,\textrm{tr}\,}}{\Big ( B^{(1)}_{\textbf{k}}(\textbf{q}') s(\textbf{k}') B^{(2)}_{\textbf{k}}(\textbf{q}')^{\dagger } s(\textbf{k}) \Big )}} = {{\,\textrm{tr}\,}}{\Big (B^{(2)}_{\textbf{k}}(\textbf{q}') s(\textbf{k}') B^{(1)}_{\textbf{k}}(\textbf{q}')^{\dagger } s(\textbf{k}) \Big )}. \end{aligned}$$

From these calculations Fock energy can be written as

C.7 $$\begin{aligned} \begin{aligned} K[P] = -\frac{1}{8 | \Omega | N_{\textbf{k}}} \sum _{\textbf{k},\textbf{q}} \sum _{\textbf{G}} V(\textbf{q}') \Big [&{{\,\textrm{tr}\,}}\Big (B^{(1)}_{\textbf{k}}(\textbf{q}') c(\textbf{k}') B^{(1)}_{\textbf{k}}(\textbf{q}')^\dagger c(\textbf{k}) \Big ) \\&+ {{\,\textrm{tr}\,}}\Big (B^{(2)}_{\textbf{k}}(\textbf{q}') c(\textbf{k}') B^{(2)}_{\textbf{k}}(\textbf{q}')^\dagger c(\textbf{k}) \Big ) \\&+ 2 \operatorname {Re}\Big ({{\,\textrm{tr}\,}}\Big (B^{(1)}_{\textbf{k}}(\textbf{q}') s(\textbf{k}') B^{(2)}_{\textbf{k}}(\textbf{q}')^\dagger s(\textbf{k}) \Big ) \Big )\Big ]. \end{aligned} \end{aligned}$$

We fix $\textbf{k}, \textbf{q}, \textbf{G}$ and define:

C.8 $$\begin{aligned} \begin{aligned}&c_i:= [c(\textbf{k})]_{ii} \qquad \qquad \qquad c_i':= [c(\textbf{k}')]_{ii} \\&s_{i}:= [s(\textbf{k})]_{ii} \qquad \qquad \qquad s_i':= [s(\textbf{k}')]_{ii} \\&b^{(1)}_{ij}:= [B^{(1)}_{\textbf{k}}(\textbf{q}')]_{ij} \\&b^{(2)}_{ij}:= [B^{(2)}_{\textbf{k}}(\textbf{q}')]_{ij}. \end{aligned} \end{aligned}$$

With these definition, the Fock energy becomes

C.9 $$\begin{aligned} \begin{aligned} \sum _{ij}&b^{(1)}_{ij} c_{j}' \overline{b^{(1)}_{ij}} c_{i} + \sum _{ij} b^{(2)}_{ij} c_{j}' \overline{b^{(2)}_{ij}} c_{i} + 2 \operatorname {Re}\Big [\sum _{ij} b^{(1)}_{ij} s_{j}' \overline{b^{(2)}_{ij}} s_{i} \Big ] \\&= \sum _{ij} (|b^{(1)}_{ij}|^{2} + |b^{(2)}_{ij}|^{2}) c_{j}' c_{i} + 2 \operatorname {Re}[ b^{(1)}_{ij} \overline{b^{(2)}_{ij}}] s_{j}' s_{i}. \end{aligned} \end{aligned}$$

Recalling the trigonometric product-to-sum rules we have

C.10 $$\begin{aligned} c_i c_j'  &   = \cos {(\theta _i(\textbf{k}))} \cos {(\theta _j(\textbf{k}'))} = \frac{1}{2} \bigg ( \cos {(\theta _i(\textbf{k}) - \theta _j(\textbf{k}'))} + \cos {(\theta _i(\textbf{k}) + \theta _j(\textbf{k}'))} \bigg ) \nonumber \\[1ex] s_i s_j'  &   = \sin {(\theta _i(\textbf{k}))} \sin {(\theta _j(\textbf{k}'))} = \frac{1}{2} \bigg ( \cos {(\theta _i(\textbf{k}) - \theta _j(\textbf{k}'))} - \cos {(\theta _i(\textbf{k}) + \theta _j(\textbf{k}'))} \bigg ).\nonumber \\ \end{aligned}$$

Therefore, for fixed *i*, *j* we have

C.11 $$\begin{aligned} \begin{aligned} (|b^{(1)}_{ij}|^{2} + |b^{(2)}_{ij}|^{2}) c_{i} c_{j}' + 2 \operatorname {Re}[&b^{(1)}_{ij} \overline{b^{(2)}_{ij}}] s_{i} s_{j}' \\ = \frac{1}{2} (|b^{(1)}_{ij}|^{2} + |b^{(2)}_{ij}|^{2})&\bigg ( \cos {(\theta _i(\textbf{k}) - \theta _j(\textbf{k}'))} + \cos {(\theta _i(\textbf{k}) + \theta _j(\textbf{k}'))} \bigg ) \\ + \operatorname {Re}[ b^{(1)}_{ij} \overline{b^{(2)}_{ij}}]&\bigg ( \cos {(\theta _i(\textbf{k}) - \theta _j(\textbf{k}'))} - \cos {(\theta _i(\textbf{k}) - \theta _j(\textbf{k}'))} \bigg ). \end{aligned} \end{aligned}$$

But since

C.12 $$\begin{aligned} |b^{(1)}_{ij}|^{2} + |b^{(2)}_{ij}|^{2} \pm 2 \operatorname {Re}[ b^{(1)}_{ij} \overline{b^{(2)}_{ij}}] = | b^{(1)}_{ij} \pm b^{(2)}_{ij} |^2 \end{aligned}$$

this simplifies to

C.13 $$\begin{aligned} \frac{1}{2} | b^{(1)}_{ij} + b^{(2)}_{ij} |^2 \cos {(\theta _i(\textbf{k}) - \theta _j(\textbf{k}'))} + \frac{1}{2} | b^{(1)}_{ij} - b^{(2)}_{ij} |^2 \cos {(\theta _i(\textbf{k}) + \theta _j(\textbf{k}'))}. \end{aligned}$$

Combining these calculations together we finally find that

C.14 $$\begin{aligned} K[P]  &   = \frac{1}{8 | \Omega | N_{\textbf{k}}} \sum _{\textbf{k},\textbf{q}} \sum _{\textbf{G}} V(\textbf{q}') \sum _{ij} \bigg \{ | b^{(1)}_{ij} + b^{(2)}_{ij} |^2 \cos {(\theta _i(\textbf{k}) - \theta _j(\textbf{k}'))}\nonumber \\  &   + | b^{(1)}_{ij} - b^{(2)}_{ij} |^2 \cos {(\theta _i(\textbf{k}) + \theta _j(\textbf{k}'))} \bigg \}.\nonumber \\ \end{aligned}$$

## Real Space Conditions for Theorem 5

### Lemma D.1

For all orthogonal projectors $\Pi$, the following statements are equivalent:

1. There exists $\textbf{G}'$, so that $\Vert (I - \Pi ) A_{\textbf{k}}(\textbf{G}') \Pi \Vert > 0$.
2. There exists $\textbf{r}$, so that $\Vert (I - \Pi ) \rho _{\textbf{k},\textbf{k}}(\textbf{r}) \Pi \Vert > 0$.

This lemma connects the second condition of Theorem 5 to a property of the real space functions $u_{n\textbf{k}}(\textbf{r})$ which is easier to check in practice. For the special case that the system has four bands, similar to Corollary 7, can derive the following simpler condition:

### Lemma D.2

Suppose that the system has four flat bands then the following are equivalent for all $\textbf{k}$

1. For all non-trivial orthogonal projectors, $\Pi$ exists an $\textbf{r}$ so that
  $$\begin{aligned} \Vert (I - \Pi ) \rho _{\textbf{k},\textbf{k}}(\textbf{r}) \Pi \Vert > 0 \end{aligned}$$
2. There exist $\textbf{r}, \textbf{r}'$ so that $[ \rho _{\textbf{k},\textbf{k}}(\textbf{r}), \rho _{\textbf{k},\textbf{k}}(\textbf{r}') ] \ne 0$.

Furthermore, to prove $[ \rho _{\textbf{k},\textbf{k}}(\textbf{r}), \rho _{\textbf{k},\textbf{k}}(\textbf{r}') ] \ne 0$ it suffices to show there exist $\textbf{r}, \textbf{r}'$ so that

D.1 $$\begin{aligned} \det { \begin{bmatrix} \Vert u_{1\textbf{k}}(\textbf{r})\Vert ^2 - \Vert u_{2\textbf{k}}(\textbf{r})\Vert ^2 &  \langle u_{1\textbf{k}}(\textbf{r}), u_{2\textbf{k}}(\textbf{r})\rangle \\ \Vert u_{1\textbf{k}}(\textbf{r}')\Vert ^2 - \Vert u_{2\textbf{k}}(\textbf{r}')\Vert ^2 &  \langle u_{1\textbf{k}}(\textbf{r}'), u_{2\textbf{k}}(\textbf{r}')\rangle \end{bmatrix}} \ne 0 \end{aligned}$$

### Proof

One may easily verify that $\rho _{\textbf{k},\textbf{k}}(\textbf{r})$ is a $2 \times 2$ Hermitian matrix for all $\textbf{r}$. As a consequence, since $\Pi$ is a non-trivial projection, $\Vert (I - \Pi ) \rho _{\textbf{k},\textbf{k}}(\textbf{r}) \Pi \Vert = 0$ if and only if $\Pi = \vert v\rangle \langle v\vert$ and $(I - \Pi ) = \vert v^\perp \rangle \langle v^\perp \vert$ where $\vert v\rangle$ and $\vert v^\perp \rangle$ are a complete basis of eigenvectors for $\rho _{\textbf{k},\textbf{k}}(\textbf{r})$. Therefore, $\Vert (I - \Pi ) \rho _{\textbf{k},\textbf{k}}(\textbf{r}) \Pi \Vert = 0$ for all $\textbf{r}$ if and only if the set $\{ \rho _{\textbf{k},\textbf{k}}(\textbf{r}): \textbf{r}\in \Omega \}$ are mutually commuting. Hence, $\Vert (I - \Pi ) \rho _{\textbf{k},\textbf{k}}(\textbf{r}) \Pi \Vert > 0$ for all non-trivial $\Pi$ if and only if there exist $\rho _{\textbf{k},\textbf{k}}(\textbf{r})$ and $\rho _{\textbf{k},\textbf{k}}(\textbf{r}')$ which do not commute.

For the second part of the lemma, we begin by calculating the off-diagonal entries of $\rho _{\textbf{k},\textbf{k}}(\textbf{r}) \rho _{\textbf{k},\textbf{k}}(\textbf{r}')$:

D.2 $$\begin{aligned} \begin{aligned}&\rho _{\textbf{k},\textbf{k}}(\textbf{r}) \rho _{\textbf{k},\textbf{k}}(\textbf{r}') \\&= \begin{bmatrix} \Vert u_{1\textbf{k}}(\textbf{r}) \Vert ^2 &  \langle u_{1\textbf{k}}(\textbf{r}), u_{2\textbf{k}}(\textbf{r})\rangle \\ \langle u_{1\textbf{k}}(\textbf{r}), u_{2\textbf{k}}(\textbf{r})\rangle ^* &  \Vert u_{2\textbf{k}}(\textbf{r}) \Vert ^2 \end{bmatrix} \begin{bmatrix} \Vert u_{1\textbf{k}}(\textbf{r}') \Vert ^2 &  \langle u_{1\textbf{k}}(\textbf{r}'), u_{2\textbf{k}}(\textbf{r}')\rangle \\ \langle u_{1\textbf{k}}(\textbf{r}'), u_{2\textbf{k}}(\textbf{r}')\rangle ^* &  \Vert u_{2\textbf{k}}(\textbf{r}') \Vert ^2 \end{bmatrix} \\&= \begin{bmatrix} * &  d(\textbf{r},\textbf{r}') \\ \overline{d(\textbf{r}',\textbf{r})} &  * \end{bmatrix} \end{aligned}\nonumber \\ \end{aligned}$$

where

D.3 $$\begin{aligned} d(\textbf{r},\textbf{r}') = \Vert u_{1\textbf{k}}(\textbf{r}) \Vert ^2 \langle u_{1\textbf{k}}(\textbf{r}'), u_{2\textbf{k}}(\textbf{r}')\rangle + \Vert u_{2\textbf{k}}(\textbf{r}') \Vert ^2 \langle u_{1\textbf{k}}(\textbf{r}), u_{2\textbf{k}}(\textbf{r})\rangle \end{aligned}$$

Therefore, using that $(\rho _{\textbf{k},\textbf{k}}(\textbf{r}) \rho _{\textbf{k},\textbf{k}}(\textbf{r}'))^\dagger = \rho _{\textbf{k},\textbf{k}}(\textbf{r}') \rho _{\textbf{k},\textbf{k}}(\textbf{r})$ we have

D.4 $$\begin{aligned} [ \rho _{\textbf{k},\textbf{k}}(\textbf{r}), \rho _{\textbf{k},\textbf{k}}(\textbf{r}') ] = \begin{bmatrix} * &  d(\textbf{r},\textbf{r}') - d(\textbf{r}',\textbf{r}) \\ \overline{d(\textbf{r}',\textbf{r})} - \overline{d(\textbf{r},\textbf{r}')} &  * \end{bmatrix}. \end{aligned}$$

Now we calculate

D.5 $$\begin{aligned} \begin{aligned} d(\textbf{r},\textbf{r}') - d(\textbf{r}',\textbf{r}) =&\Big ( \Vert u_{1\textbf{k}}(\textbf{r}) \Vert ^2 - \Vert u_{2\textbf{k}}(\textbf{r}) \Vert ^2 \Big ) \langle u_{1\textbf{k}}(\textbf{r}'), u_{2\textbf{k}}(\textbf{r}')\rangle \\&- \Big ( \Vert u_{1\textbf{k}}(\textbf{r}') \Vert ^2 - \Vert u_{2\textbf{k}}(\textbf{r}') \Vert ^2 \Big ) \langle u_{1\textbf{k}}(\textbf{r}), u_{2\textbf{k}}(\textbf{r})\rangle \\ =&\det { \begin{bmatrix} \Vert u_{1\textbf{k}}(\textbf{r})\Vert ^2 - \Vert u_{2\textbf{k}}(\textbf{r})\Vert ^2 &  \langle u_{1\textbf{k}}(\textbf{r}), u_{2\textbf{k}}(\textbf{r})\rangle \\ \Vert u_{1\textbf{k}}(\textbf{r}')\Vert ^2 - \Vert u_{2\textbf{k}}(\textbf{r}')\Vert ^2 &  \langle u_{1\textbf{k}}(\textbf{r}'), u_{2\textbf{k}}(\textbf{r}')\rangle \end{bmatrix}} \end{aligned} \end{aligned}$$

which completes the proof. $\square$

As for the first condition of Theorem 5, we observe that

D.6 $$\begin{aligned} {{\,\textrm{tr}\,}}{(A_{\textbf{k}}(\textbf{G}))} = \int _{\Omega } e^{-i \textbf{G}\cdot \textbf{r}} \sum _{m} \Vert u_{m\textbf{k}}(\textbf{r}) \Vert ^{2} \,\textrm{d}\textbf{r}\end{aligned}$$

where the norm is understood to sum over $\sigma , j$. And we recall the following simple result:

### Lemma D.3

Let $\Gamma$ be a d-dimensional lattice in $\mathbb {R}^d$ and $f:{\mathbb {R}}^d/\Gamma \rightarrow {\mathbb {R}}$ a continuous function, then *f* is even, i.e. $\overline{f(-{\textbf{r}})}=f({\textbf{r}})$ if and only if

$$\begin{aligned} \operatorname {Im}{\hat{f}}(\textbf{G}) =0 \text { for all }{\textbf{G}} \in \Gamma ^*,\end{aligned}$$

where ${\hat{f}}$ is the Fourier transform of *f*.

### Proof

Indeed, assuming $\overline{f(-{\textbf{r}})}=f({\textbf{r}})$, we have using (2.19)

$$\begin{aligned}\begin{aligned} \overline{{\hat{f}}(\textbf{G})}&= \overline{\int _{{\mathbb {R}}^d/\Gamma } f(\textbf{r}) e^{-i\textbf{G}\cdot \textbf{r}} \ d\textbf{r}}= \int _{{\mathbb {R}}^d/\Gamma } \overline{f(\textbf{r})} e^{i\textbf{G}\cdot \textbf{r}} \ d\textbf{r}\\&=\int _{{\mathbb {R}}^d/\Gamma } \overline{f(-\textbf{r})} e^{-i\textbf{G}\cdot \textbf{r}} \ d\textbf{r}= \int _{{\mathbb {R}}^d/\Gamma } f(\textbf{r}) e^{-i\textbf{G}\cdot \textbf{r}} \ d\textbf{r}= {\hat{f}}(\textbf{G}). \end{aligned} \end{aligned}$$

The converse implication is easily observed from the Fourier series. $\square$

By Lemma D.3, we conclude that $\operatorname {Im}{{\,\textrm{tr}\,}}{(A_{\textbf{k}}(\textbf{G}))} \ne 0$ if and only if the function

D.7 $$\begin{aligned} \textbf{r}\mapsto \sum _{m} \Vert u_{m\textbf{k}}(\textbf{r}) \Vert ^{2} \end{aligned}$$

is not an even function.

## Footnotes

1 With slight abuse of language, we refer to the statement that the ferromagnetic Slater determinant states are the only elements of $\mathcal {S}$ which are exact ground states of ${\hat{H}}$ as “unique states”, even though technically there are two of them.

2 In the sense that $\det (\sum _{\vert \alpha \vert = s}a_{\alpha }(x)\xi ^{\alpha }) \ne 0$ for every $\xi \ne 0$.
